# Resilience to autosomal dominant Alzheimer’s disease in a Reelin-COLBOS heterozygous man

**DOI:** 10.1038/s41591-023-02318-3

**Published:** 2023-05-15

**Authors:** Francisco Lopera, Claudia Marino, Anita S. Chandrahas, Michael O’Hare, Nelson David Villalba-Moreno, David Aguillon, Ana Baena, Justin S. Sanchez, Clara Vila-Castelar, Liliana Ramirez Gomez, Natalia Chmielewska, Gabriel M. Oliveira, Jessica Lisa Littau, Kristin Hartmann, Kyungeun Park, Susanne Krasemann, Markus Glatzel, Dorothee Schoemaker, Lucia Gonzalez-Buendia, Santiago Delgado-Tirado, Said Arevalo-Alquichire, Kahira L. Saez-Torres, Dhanesh Amarnani, Leo A. Kim, Randall C. Mazzarino, Harper Gordon, Yamile Bocanegra, Andres Villegas, Xiaowu Gai, Moiz Bootwalla, Jianling Ji, Lishuang Shen, Kenneth S. Kosik, Yi Su, Yinghua Chen, Aaron Schultz, Reisa A. Sperling, Keith Johnson, Eric M. Reiman, Diego Sepulveda-Falla, Joseph F. Arboleda-Velasquez, Yakeel T. Quiroz

**Affiliations:** 1 Neuroscience Group of Antioquia, Medicine School, University of Antioquia, Medellín, Colombia; 2 Medicine School, University of Antioquia, Medellín, Colombia; 3 Schepens Eye Research Institute of Mass Eye and Ear and Department of Ophthalmology at Harvard Medical School, Boston, MA USA; 4 Institute of Neuropathology, University Medical Center Hamburg-Eppendorf, Hamburg, Germany; 5 Department of Neurology at Harvard Medical School, Massachusetts General Hospital, Boston, MA USA; 6 Department of Psychiatry at Harvard Medical School, Massachusetts General Hospital, Boston, MA USA; 7 Center for Personalized Medicine, Department of Pathology and Laboratory Medicine, Children’s Hospital Los Angeles, Los Angeles, CA USA; 8 Department of Pathology, Keck School of Medicine of University of Southern California, Los Angeles, CA USA; 9 Neuroscience Research Institute, Department of Molecular Cellular Developmental Biology, University of California, Santa Barbara, CA USA; 10 The Banner Alzheimer’s Institute, Phoenix, AZ USA; 11 Department of Neurology at Harvard Medical School, Brigham and Women’s Hospital, Boston, MA USA; 12 Department of Radiology at Harvard Medical School, Massachusetts General Hospital, Boston, MA USA; 13 University of Arizona, Tucson, AZ USA; 14 Arizona State University, Tucson, AZ USA; 15 Neurogenomics Division, Translational Genomics Research Institute, Phoenix, AZ USA

**Keywords:** Molecular neuroscience, Alzheimer's disease

## Abstract

We characterized the world’s second case with ascertained extreme resilience to autosomal dominant Alzheimer’s disease (ADAD). Side-by-side comparisons of this male case and the previously reported female case with ADAD homozygote for the *APOE3* Christchurch (*APOECh*) variant allowed us to discern common features. The male remained cognitively intact until 67 years of age despite carrying a *PSEN1*-E280A mutation. Like the *APOECh* carrier, he had extremely elevated amyloid plaque burden and limited entorhinal Tau tangle burden. He did not carry the *APOECh* variant but was heterozygous for a rare variant in *RELN* (H3447R, termed *COLBOS* after the Colombia–Boston biomarker research study), a ligand that like apolipoprotein E binds to the VLDLr and APOEr2 receptors. *RELN-COLBOS* is a gain-of-function variant showing stronger ability to activate its canonical protein target Dab1 and reduce human Tau phosphorylation in a knockin mouse. A genetic variant in a case protected from ADAD suggests a role for *RELN* signaling in resilience to dementia.

## Abstract (web summary)

Case report of an individual heterozygous for a rare *RELN-COLBOS* variant that confers resilience, via a gain-of-function mechanism, to Alzheimer’s disease.

## Main

We have characterized about 1,200 individuals carrying the presenilin 1 (*PSEN1*) E280A mutation from the world’s largest known kindred with autosomal dominant Alzheimer’s disease (ADAD). Carriers of the *PSEN1-*E280A mutation develop mild cognitive impairment (MCI) by the median age of 44 years (95% confidence interval (CI) = 43–45) and dementia by 49 years (95% CI = 49–50)^1^, with rare exceptions^2^. We previously reported a female carrying the *PSEN1*-E280A mutation with two copies of the *APOE3* Christchurch (*APOECh*) (R136S) gene variant who remained cognitively unimpaired for nearly 30 years after the expected age at clinical onset^2^.

In this article, we report the clinical, in vivo neuroimaging, genetic and neuropathological characteristics of a male case with the *PSEN1*-E280A mutation from the same population also presenting with an extreme phenotype of delayed age at clinical onset of ADAD.

## Results

### Case report

We identified a male carrier of the *PSEN1*-E280A mutation who remained cognitively intact until age 67. He completed 5 years of formal education in his home country (Colombia) and worked until he retired in his early 60s. He was married and had two children. First cognitive assessment at age 67 revealed limited verbal learning skills and language difficulties in the context of functional independence. The patient was diagnosed with MCI, characterized by short-term memory and verbal fluency decline at age 70.

At age 72, his language had deteriorated further, progressing to mild dementia (Supplementary Table 1). Cognitive decline was preceded by a urinary tract infection-related episode of septic shock. At age 73, he required assistance with basic and instrumental activities of daily living, and met criteria for moderate dementia. He died at the age of 74 years from aspiration pneumonia; his relatives agreed to a brain donation for neuropathological study.

His sister also carried the *PSEN1*-E280A mutation, had severe dementia when she was first evaluated at age 64 and progressed to end-stage dementia at age 72 (see the pedigree in Supplementary Fig. 1). According to the family, she had hypothyroidism, hypertension, depression and cognitive decline at age 58 and developed dementia at age 61. Although less protected than her brother, her MCI began 14 years and her dementia 12 years later than expected for this population. Dementia was preceded by ocular trauma and tibial fracture after a fall, which required surgery under general anesthesia. She died at age 73 of sepsis of pulmonary origin. Additional clinical details about the cases can be found in the supplementary results section of the Supplementary Information.

**Fig. 1:**
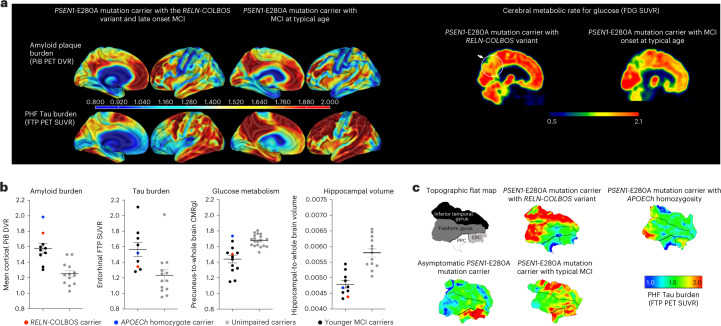
PET imaging of the *RELN-COLBOS* (H3447R) carrier. **a**, Representative PiB PET amyloid and FTP Tau PET imaging of the male case with *RELN-COLBOS* (left) compared to a *PSEN1-*E280A mutation carrier with MCI at a typical age (right). For both measurements, specific binding of the tracer is represented using a color-coded scale with blue being the lowest (DVR or SUVR = 0.8) and red being the highest (DVR or SUVR = 2.00) degree of binding. Right, representative FDG PET precuneus cerebral metabolic rate for glucose (CMRgI) of the male case with *RELN-COLBOS* (left) compared to a *PSEN1*-E280A carrier with MCI at a typical age (right). Binding affinity of the dye is represented using a color-coded scale with blue being the lowest (SUVR = 0.5) and red being the highest (SUVR = 2.1) degree of binding. PHF, paired helical filament. **b**, Dot plot analysis of the imaging measurements shown in **a** for amyloid and Tau burden, glucose metabolism and hippocampal volume. Brain imaging measurements of the male case with *RELN-COLBOS* (red dot) compared to the previously published *APOECh* homozygote female (blue dot), unimpaired *PSEN1*-E280A carriers (gray dots, *n* = 18 for the glucose metabolism panel, *n* = 13 for all other panels) and younger carriers of the MCI *PSEN1*-E280A mutation (black dots, *n* = 7 for the Tau burden plot, *n* = 8 for the amyloid burden and hippocampal volume plots, *n* = 11 for glucose metabolism)^2^. Some previously published data points are included in the figures because they are the only available data for comparison^2^. Data are expressed as individual values with the mean ± s.e.m. **c**, Anatomical details of Tau burden in the temporal cortex. Flat map representations of the right hemisphere temporal lobe cortex for regions of interest (ROIs) (top left, ERC), with Tau PET (FTP) overlay for four cases. The asymptomatic *PSEN1*-E280A carrier was 38 years old; the *PSEN1*-E280A carrier with typical MCI was 44 years old. The male carrier of *RELN-COLBOS* was notable for having relatively lower Tau burden in the medial temporal regions (ERC and PPC), compared to typical *PSEN1*-E280A mutation carriers.

**Extended Data Fig. 1:**
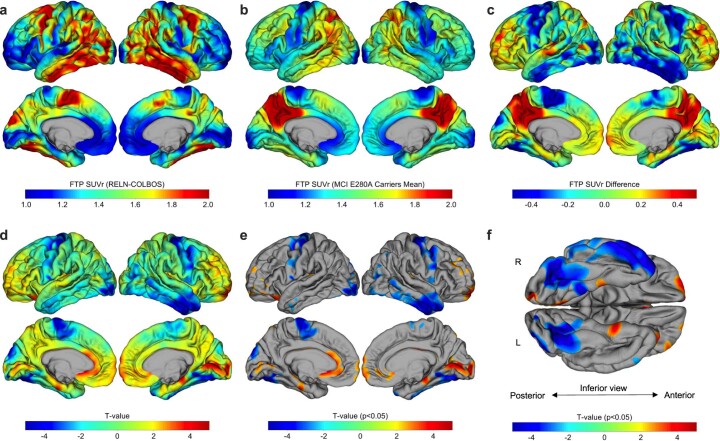
Whole-cortex (vertex-wise) comparison of *RELN-COLBOS* carrier to typical MCI *PSEN1 E280A* carriers’ tau PET. This analysis confirmed that compared to other *PSEN1* E280A carriers who developed MCI at younger ages, the *RELN-COLBOS* carrier had relatively spared tau PET signal in the medial temporal lobe (entorhinal cortex / parahippocampal gyrus), but not in other areas including temporal neocortex. **a**, Cortical surface projection of vertex-wise tau PET (Flortaucipir, FTP) standardized uptake value ratio (SUVr) in the *RELN-COLBOS* carrier. **b**, Mean tau PET image of typical MCI *PSEN1* E280A carriers (n = 7). **c**, difference analysis between images (**b**) and (**a**): negative values indicate areas where *RELN-COLBOS* carrier had higher tau PET signal compared to other carriers, and vice-versa. **d**, T-value for one-sample 2-sided t-tests comparing other *PSEN1* E280A carriers to the *RELN-COLBOS* patient. Directionality of effect size is the same as (**c**). Panel **e** shows T-values as in (**d**) masked by p value < 0.05 (uncorrected). **f**, Inferior / close-up view of surfaces shown in (**e**).

**Extended Data Fig. 2:**
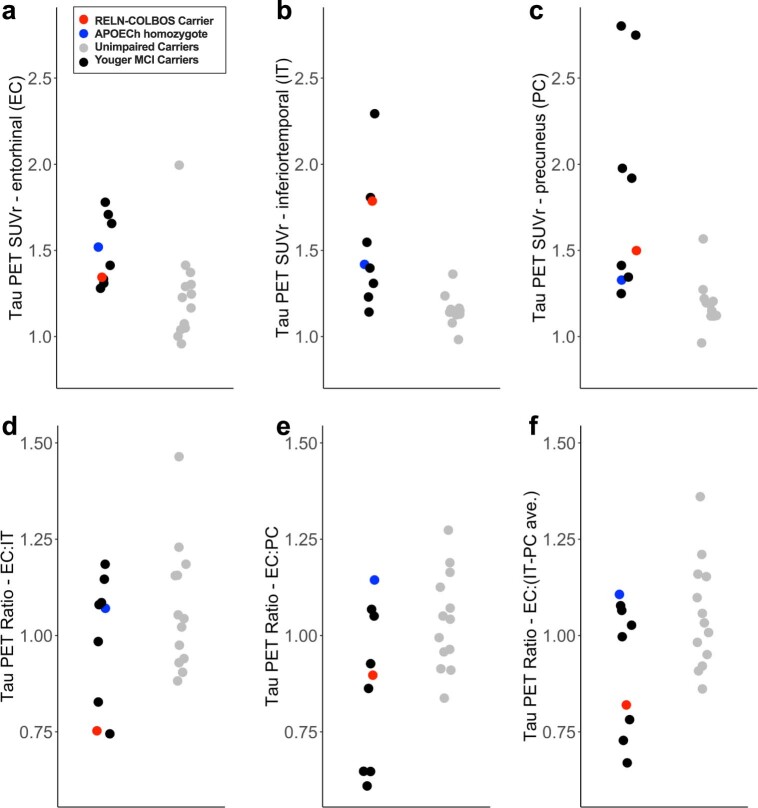
Regional comparison of tau PET burden in *RELN-COLBOS* carrier versus other *PSEN1* E280A carriers. This analysis confirmed that compared to other *PSEN1* E280A carriers who developed MCI at younger ages, the *RELN-COLBOS* carrier had relatively spared tau pathology in the medial temporal lobe (entorhinal cortex / parahippocampal gyrus), but not in other areas including temporal neocortex (inferior temporal). **a-c**, Regional tau PET signal (Flortaucipir, FTP) standardized uptake value ratio (SUVr) in three regions of interest: entorhinal (EC, **a**), inferior temporal (IT, **b**), and precuneus (PC, **C**). **d-f**, Ratios of tau PET uptake in medial temporal (EC) and neocortical (IT, PC) regions. **d**, EC:IT ratio, **e**, EC:PC ratio, **f**, ratio of EC to average of IT and PC (neocortical average). The tau pattern of the *RELN-COLBOS* patient was notable for the relative involvement of medial temporal versus temporal neocortex, particularly given the older age of the *RELN-COLBOS* patient compared to other carriers.

The male patient was enrolled in the Colombia–Boston biomarker research study (COLBOS) and underwent neuroimaging examinations at the Massachusetts General Hospital (MGH) when he was 73 years old (see Supplementary Table 2 for the demographic information). Amyloid positron emission tomography (PET), measured using cortical-to-cerebellar Pittsburgh compound B (PiB), revealed that the individual’s levels of cortical amyloid beta (Aβ) plaque burden were higher (distribution volume ratio (DVR) = 1.77) compared to that of younger impaired carriers from this kindred with a typical age at onset (mean DVR = 1.51 ± 0.13; Fig. 1a,b). Tau tangle burden in the inferior temporal lobe, measured by flortaucipir (FTP), was similar to that seen in younger *PSEN1-*E280A impaired carriers with typical age at onset (standardized uptake value ratio (SUVR) = 1.78). However, he had relatively limited Tau pathology in the entorhinal cortex (ERC) (SUVR = 1.34; Fig. 1a,c) and in other neocortical regions, such as the posterior cingulate cortex (PCC) and precuneus (SUVR PCC = 1.51; SUVR precuneus = 1.49; Fig. 1a), which usually show greater levels of Tau pathology in *PSEN1*-E280A carriers who develop MCI and dementia at a typical age^3^ (Fig. 1, Supplementary Fig. 2 and Extended Data Figs. 1 and 2). Sparing of the ERC from Tau pathology is a salient feature in the case with *RELN-COLBOS* that could be critical for the protection phenotype.

Measurements of metabolic rate for glucose in the precuneus and whole brain region using ^18^F-fluorodeoxyglucose (FDG) PET showed a slightly higher level of glucose metabolism compared to the mean levels of typical MCI carriers from the kindred, who were much younger (Fig.1a,b). He had brain atrophy, measured by magnetic resonance imaging (MRI)-based hippocampal and whole brain volume typical of MCI carriers. These imaging findings suggest that in this patient and in the *APOECh* homozygote case^2^, protection against ADAD dementia occurred even in the face of high amyloid burden (Fig. 1a,b). Additional imaging and biomarker analyses are reported in the supplementary results of the Supplementary Information.

Our genetic analyses confirmed that the individual was a heterozygote carrier of the *PSEN1*-E280A mutation (confirmed by single-cell RNA sequencing (scRNA-seq); Supplementary Tables 3 and 4), ruled out the presence of the *APOECh* mutation (the individual was *APOE3/APOE3* and had a normal blood lipid profile; Supplementary Table 5) and identified a heterozygous variant in *RELN* (chromosome 7:g.103113302T>C, H3447R; Supplementary Fig. 1b), which we named ‘*RELN-COLBOS*’, as the most promising missense variant potentially contributing to the phenotype in the protected individual. The *RELN-COLBOS* variant was only found in the individual and his sister (Supplementary Fig. 1), who also had late-onset cognitive decline. Both were *APOE3/APOE3*. We focused on the *RELN-COLBOS* variant because it ranked in the top three candidate genes in the Genomizer priority score analysis and because the encoded protein RELN modulates Tau phosphorylation^4^ and is functionally closely related to *APOE*, the gene mutated in the other case with extreme protection against ADAD^2^. A more detailed description of the genetic analysis is reported in the genetic analysis section of the Supplementary Information.

Our genetic analyses also identified other variants of potential interest in the male individual including: (1) a noncoding variant in the amyloid beta precursor protein (*APP*) gene (3′-untranslated region, chromosome 21:g.27253263T>C; and (2) a noncoding variant in the calmodulin 2 (*CALM2*) gene (intronic, chromosome 2:g.47394764C>G). The levels of expression of *APP* in peripheral blood mononuclear cells from *RELN* H3447R carrier and noncarriers were very low and not substantially different. Therefore, the significance of the *APP* variant is uncertain, although it is unlikely to be implicated considering the robust amyloid pathology observed in the individual (Fig.1a,b). This *APP* variant (g.27253263T>C) was not seen in the *APOECh* case based on whole-genome sequencing (WGS). The *CALM2* variant is predicted as intronic for the canonical transcript encoding a protein with 149 amino acids (P0DP24) and it would result in a Cys9Ser in a predicted longer transcript encoding for a protein with 196 amino acids (E7EMB3). This *CALM2* variant has not been reported in the ClinVar database and has low in silico pathogenicity scores. We also could not find a per-variant regulatory score in the Encode database for this variant^5^. The significance of this variant is uncertain, although it is unlikely to be implicated because there is no proteomic evidence of the region of the variant producing any detectable protein in the PeptideAtlas^6^. Further studies may be conducted to examine the extent to which these or other variants contribute to the observed phenotype.

**Fig. 2:**
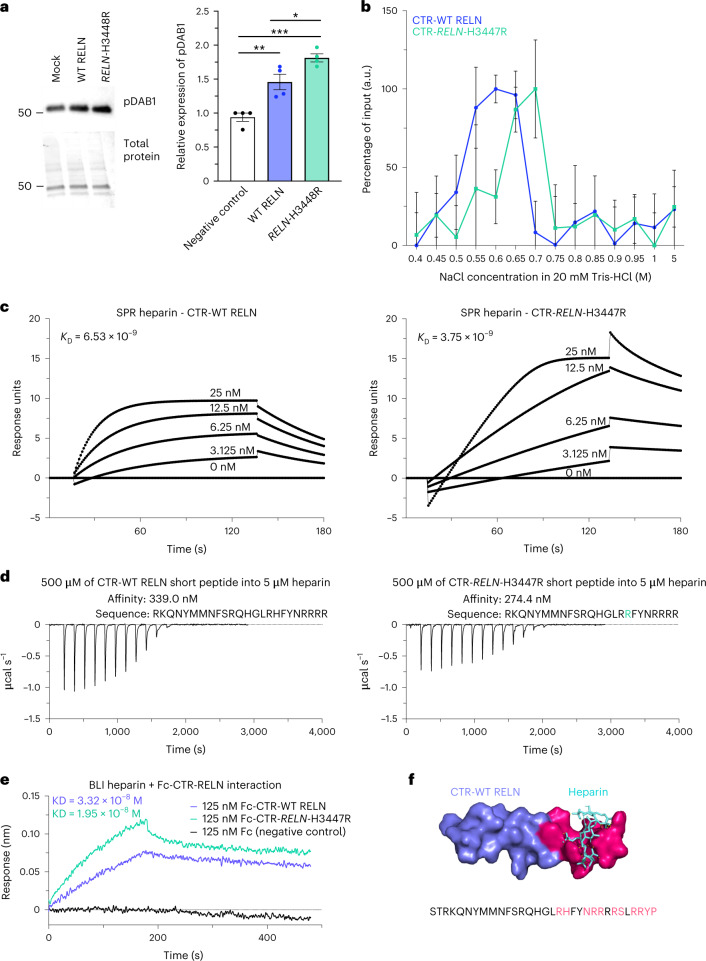
The *RELN*-H3448R variant enhances Dab1 signaling and the affinity of CTR-RELN to heparin. **a**, Representative western blotting of pDab1 levels (top) and total protein staining (bottom) levels in primary mouse cortical neurons treated with full-length WT RELN or *RELN*-H3448R, mouse ortholog of *RELN*-H3447R (mock, *P* < 0.0029 and WT RELN, *P* = 0.0246). Data are presented as the mean ± s.e.m. and were analyzed using a Kruskal–Wallis test with Dunn post hoc analysis for multiple comparisons of *n* = 4 independent biological experiments. **b**, Spectroscopic analysis of heparin chromatography fractions of WT CTR-RELN (blue plot) and the *CTR-RELN*-H3447R mutant (green plot) eluted at increasing gradients of NaCl (0.05 M NaCl step gradient). Data are expressed as the percentage of input over 0.4–5 M NaCl gradient fractions. Data show that 0.55 M NaCl can displace WT CTR-RELN binding from a heparin column. The affinity for heparin of CTR-RELN increases in the presence of the H3447R mutation, as suggested by the shift of the peak with maximum height of the eluted fraction from 0.55 to 0.7 M NaCl. *n* = 3 independent chromatography experiments. The error bars represent the s.e.m. **c**, Representative sensorgrams of the binding analysis between chip sensors coated with heparin and 0–25 nM increasing concentrations of *CTR-RELN* variants. Data are expressed as response units per second. The equilibrium disassociation constant (K_D_) for each SPR analysis are shown inside the graph and support the difference in affinity binding between heparin and the *CTR-RELN* variants: H3447R (right plot, K_D_ = 3.75 × 10^−9^ M^-1^ s^-1^) > H3347 (left plot, K_D_ = 6.53 × 10^−9^ M^-1^ s^-1^). The sensorgrams of CTR-RELN with the H3447K and H3447D control variants are reported in Supplementary Fig. 6 for comparison. **d**, Isothermal calorimetry measurements of short-variant *WT CTR-RELN* (left) and *CTR-RELN*-H3447R (right) titrated with 5 μM heparin. Affinity calculations are reported above each plot. **e**, Binding analysis via BLI between Fc-fusion WT CTR-RELN and H3447R and a heparin-coated biosensor. Association (k_a_) and dissociation constants (k_d_) were used to calculate the K_D_ that is displayed in the plot. **f**, Docking of WT CTR-RELN (purple) with a representative heparin molecule (cyan). Amino acids in CTR-RELN that have polar contacts with heparin are highlighted in magenta. Source data

To strengthen our understanding of the genetic implications of the *RELN-COLBOS* variant, we conducted both in vitro and in vivo molecular genetic studies. These studies aimed to provide further evidence to support our initial findings and increase confidence in our attribution of the variant’s genetic effects. The binding of RELN results in the clustering and activation of VLDLr and APOEr2, initiating a signaling pathway that leads to the activation of Dab1 (refs. ^4,7–9^). Our studies found that RELN-COLBOS significantly increased Dab1 phosphorylation compared to wild-type (WT) RELN (Fig. 2a, *P* = 0.0246) in primary culture mouse cortical neurons (Supplementary Figs. 3 and 4). We also examined RELN-COLBOS binding to its receptors to better understand the potential mechanisms for the observed enhanced signaling. Our findings from cell-free binding assays suggest that RELN-COLBOS did not directly impact binding of RELN to VLDLr or APOEr2, suggesting that other mechanisms were involved (Supplementary Fig. 5).

The C-terminal region of RELN (CTR-RELN), where the H3447R variant is located, modulates signaling indirectly via interactions with previously unidentified coreceptors on cell membranes^10^. Recently, neuropilin 1 (NRP1) was identified as a coreceptor via binding to a fragment of CTR-RELN consisting of the last six amino acids. This fragment is removed by furin protease. The uncleaved ‘long’ CTR-RELN interacts with NRP1 and the cleaved ‘short’ CTR-RELN does not^11^. CTR-RELN has many basic amino acids that are extremely well conserved across species^10^, which we hypothesized could also mediate interactions with glycosaminoglycans (GAGs) on cell membranes. Interactions with GAGs are a rate-limiting step in the interaction of the apolipoprotein E (APOE) protein with some receptors^12^, whereas the role of GAGs in RELN activity has not been fully resolved^10,13^. We used affinity chromatography to examine heparin (a type of GAG) binding of recombinant CTR-RELN peptides. WT CTR-RELN and H3447R CTR-RELN bound to heparin. H3447R CTR-RELN required higher NaCl concentrations to be released from the heparin column, suggesting increased binding affinity (Fig. 2b). Surface plasmon resonance (SPR) measures of kinetic constants showed that the affinity of CTR-RELN H3447R is about twice that observed in the WT CTR-RELN (Fig. 2c and Supplementary Fig. 6).

**Fig. 3:**
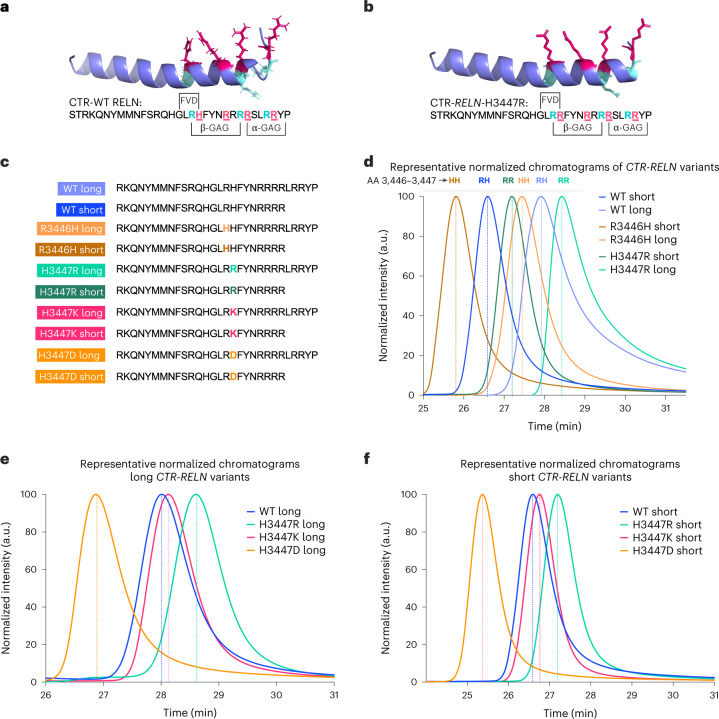
Binding profiles of CTR-RELN variants with heparin. **a**,**b**, Representative in silico models depicting the orientation of basic amino acids in the heparin-binding motif (highlighted with colors) using the WT CTR-RELN NMR structure (**a**). For the H3447R CTR-RELN variant (**b**) the model was determined by a homology-based model of WT CTR-RELN that was calculated by Swiss-Model. Position 3,447 orients in the same direction as most other arginines (magenta). Arginines in positions 3,446, 3,453 and 3,457 (cyan) may also interact with heparin as part of the heparin-binding motif but are oriented differently from most basic amino acids. **c**, RELN peptide variant sequences used for the HPLC analysis. **d**, Representative chromatographic profiles normalized to the maximum of each eluted peak of short or long CTR-RELN peptides with zero (R3446H, orange), one (WT, blue) or two (H3447R, green) arginines in positions 3,446–3,447, which are predicted to contribute to increased interaction with heparin and are indicated by later peak retention time in the isocratic 1 M KCl elution. **e**,**f**, Long (**e**) or short (**f**) CTR-RELN peptides with zero (R3446H, orange), one (WT, blue) or two (H3447R, green) arginines in positions 3,446–3,447 showing increased interaction with heparin for the long variants, as indicated by later peak retention time in the isocratic 1 M KCl elution. Conversely, short RELN variants have earlier peak retention times compared to long *RELN* variants (**e**). *n* = 2 independent chromatographic repeats within <0.5 min of representative peaks. Data are expressed as normalized to the maximum emission wavelength for each peak.

**Extended Data Fig. 3:**
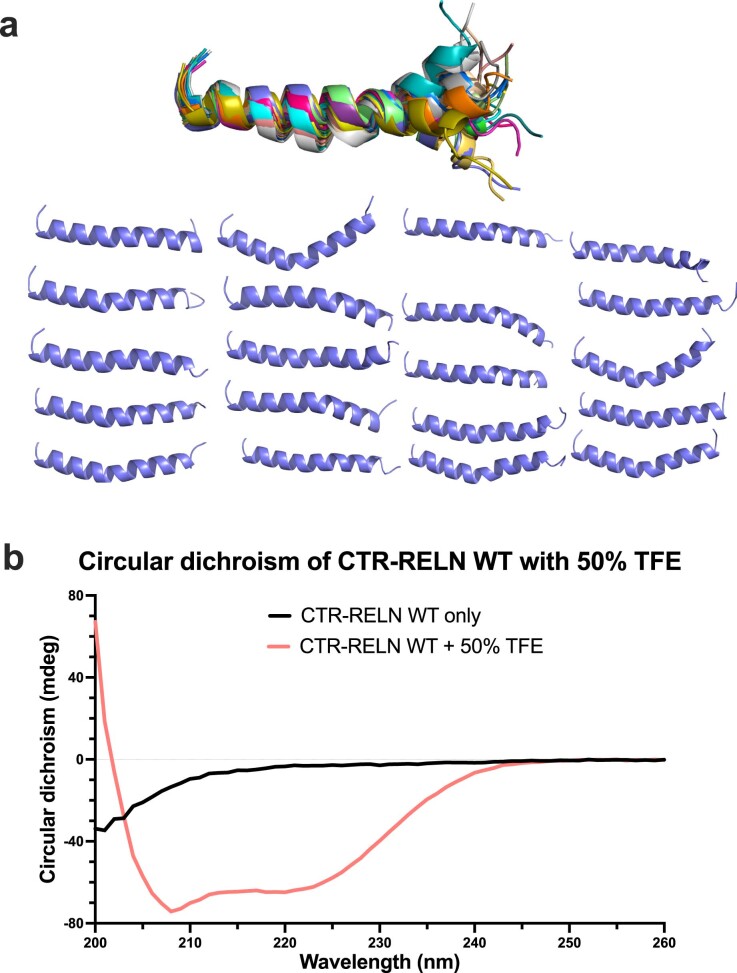
RELN CTR structural determination. **a**, *In silico* models for the 20 lowest energy structures of CTR-RELN produced by 2D NMR. **b**, Representative CD spectra of CTR-RELN without heating showing that the CTR-RELN has primarily an alpha helical structure while the peptide without 50% 2, 2, 2-Trifluoroethanol has a spectrum closest to a random coil. Data is presented as the average of four spectra. Secondary structure analysis is reported in Supplementary Table 7.

We confirmed enhanced interactions of H3447R CTR-RELN with heparin through the use of isothermal titration calorimetry (ITC) (Fig. 2d and Supplementary Table 6) and biolayer interferometry (BLI) (Fig. 2e). The association constant (K_a_) was the main contributor to the difference in equilibrium dissociation constant (K_D_) values, and the mutant CTR-RELN had a more negative Gibbs free energy in comparison to WT, suggesting that the *COLBOS* mutation enables spontaneous CTR-RELN reactions with heparin (Supplementary Table 6). Our nuclear magnetic resonance (NMR) (Figs. 2f and 3a) study revealed that CTR-RELN may have an α-helix structure including a flexible region with a domain we named ‘flexibility vertex’ (Extended Data Fig. 3a) in the presence of trifluoroethanol, although it may be unstructured under native conditions as revealed by circular dichroism (Extended Data Fig. 3b and Supplementary Table 7). Heparin-binding analyses of a library of mutant CTR-RELN peptides uncovered two binding sites for GAGs, which we named ‘α-GAG binding site’ and ‘β-GAG binding site’ (Fig. 3a). The α-GAG binding site is located in the last six amino acids and overlaps with a previously identified binding site for NRP1, which is released by furin. The β-GAG binding site is located upstream of the furin cleavage site and spans amino acids 3,446–3,451. Our research also found that CTR-RELN-COLBOS has a tenfold higher affinity for NRP1 compared to the WT version of CTR-RELN (Supplementary Table 8) due to the optimization of the β-GAG binding site. We conducted extensive studies of mutant CTR-RELN interactions with heparin by high-performance liquid chromatography (HPLC) (Fig. 3c–f), BLI (Fig. 2e) and NMR structure (Figs. 2f and 3a,b, Extended Data Fig. 3a and Supplementary Videos 1–3) to support these assertions.

**Fig. 4:**
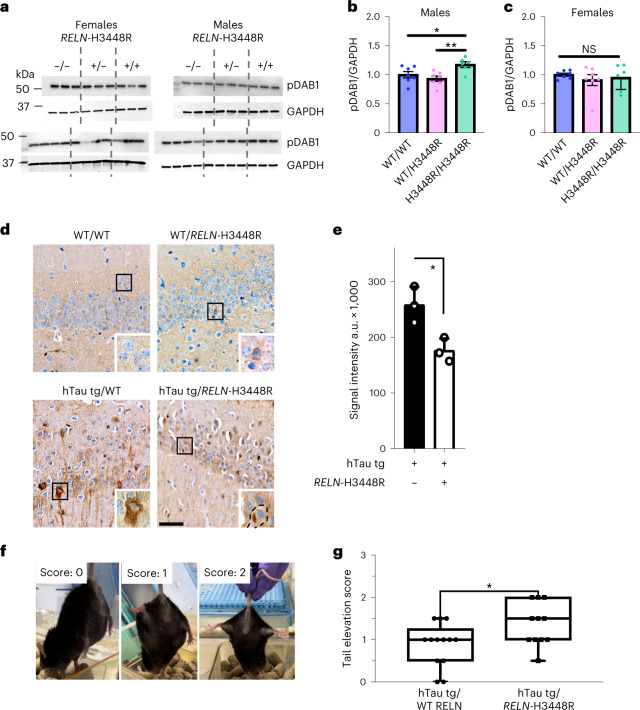
*RELN*-H3448R homozygosity promotes pDab1 signaling, reduces Tau hyperphosphorylation and preserves motor functions in mice. **a**, Representative western blots of pDab1 (top) and GAPDH levels (bottom) detected in the CB of both female (left) and male (right) mice either WT (WT/WT *RELN*), heterozygous (WT *RELN*/H3448R) or homozygous (*RELN*-H3448R/H3448R) for the *mRELN*-H3448R mutation. Levels of pDab1 were detected in 6–12-month-old mice. **b**,**c**, Quantifications of pDab1 levels normalized to GAPDH and expressed as the fold change of WT *RELN* showing a genotype effect in pDab1 levels in male mice (**b**) but not female mice (**c**). *P = 0.0284 for WT/WT, *n* = 7 mice versus H3448R/H3448R, *n* = 6 mice, *t* = 1.001, d.f. = 17; **P = 0.0037 for WT/H3448R, *n* = 7 mice versus H3448R/H3448R, *n* = 6 mice, *t* = 3.356, d.f. = 17, one-way analysis of variance (ANOVA). Data are expressed as the average ± s.e.m. Analyses of pDab1 levels in male mice at 3 months of age and in other brain regions are shown in Extended Data Fig. 5. Validation of the anti-pDab1 antibody used in **a** and **e** is reported in Supplementary Figs. 5 and 6. **d**, Representative immunohistochemistry (IHC) images from the HIC of WT/WT, WT/*RELN*-H3448R, hTau tg/WT and hTau tg/*RELN*-H3448R mice stained with hyperphosphorylated Tau (pTau) T205 antibody. hTau tg/WT mice showed neurofibrillary tangles and neuropil threads in the first region of the hippocampal circuit (CA1) and dentate gyrus, while hTau tg/*RELN*-H3448R showed Tau pathology to a lesser degree (soma of an affected neuron depicted with a dotted line). Bar scale, 100 μm. **e**, Bar graph for pTau T205 signal intensity values in hTau tg/WT (*n* = 3 mice) and hTau tg/*RELN*-H3448R mice (*n* = 3 mice). The latter showed significantly lower signal intensity. *P = 0.022, two-sided Student’s *t*-test. The error bars represent the s.d. from the mean. **f**, Representative phenotype observed during the tail elevation test and relative score (0 = severely impaired, 1 = 50% impaired, 2 = normal). **g**, Tail elevation recorded on WT *RELN*/Tau-P301L (*n* = 13 male mice) and *RELN*-H3448R/Tau-P301L crossed male mice (*n* = 11 male mice) showed a significantly improved tail elevation score in the presence of the *RELN*-H3448R variant compared to Tau-P301L mice expressing WT *RELN* (*P = 0.0305, two-tailed unpaired *t*-test, *t* = 2.313, d.f. = 22). Box plots are expressed as minimum to maximum values around the average. Source data

**Extended Data Fig. 4:**
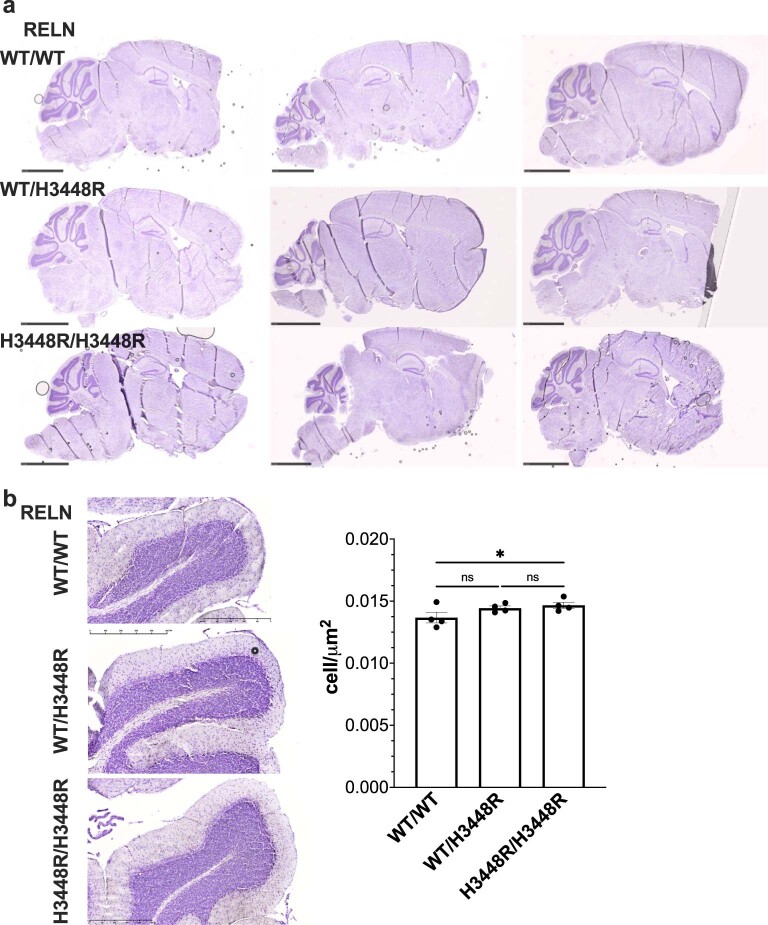
Increased neuronal density in the cerebellum’s granular layer in the presence of the H3448R *RELN* mice. presence of the H3448R *RELN* mice. **a**, Representative Cresyl violet staining of n = 3 midsagittal sections from male *RELN* mice (WT/WT, WT/H3448R, H3448R/H3448R; 5–6-month-old mice). Data is showing that the H3448R variant does not affect qualitatively the gross anatomy, nor neuronal distribution in the whole brain. Scale bar, 2.5 mm. **b**, Cresyl violet staining of the granular region of the cerebellum, indicating an increased neuronal density in the homozygotes (H3448R/H3448R) as compared to wild type (WT/WT) and heterozygote (WT/H3448R) mice. Scale bar, 500 μm. **c**, Neuron density analysis of the granular layer of the *RELN*-H3448R cerebellum in comparisons to wild type. Data indicates a significantly increased neuron counting (*p = 0.0470, n1 = n2 = 4, t = 2.300, DF = 9) in homozygotes (H3448R/H3448R) as compared with wild type (WT/WT). One-way ANOVA, followed by Fisher’s LSD test post-hoc analysis for multiple comparison of n = 4 specimens for each genotype. Data is presented as mean ± S.D. At least 2 sections and 5 fields per section were analyzed for each specimen.

Furthermore, we generated a knockin mouse model carrying the equivalent of the *RELN-COLBOS* variant (H3448R, *mRELN-H3448R* or *mRELN-COLBOS*^10^; Supplementary Fig. 7 and Supplementary Table 9) to increase confidence in our imputation of genetic implication, a common practice to study rare variants. This mouse model is viable, fertile and lacks overt structural and phenotypic brain abnormalities of RELN loss-of-function mutants (for example, cortex lamination defects, abnormal neuronal migration and cerebellar hypotrophy; Extended Data Fig. 4a)^14,15^. Our molecular analyses of cerebellum (CB) from mice with the *mRELN-COLBOS* confirmed our observation of a gain-of-function (GOF) in 6–12-month old mice for *RELN*-H3448R as determined by enhanced phosphorylation of Dab1 in males (Fig. 4a–c, *P* = 0.0284) and revealed a propensity for the formation of higher molecular protein oligomers for *RELN-COLBOS* (Supplementary Figs. 8 and 9), a feature that may be critical for enhanced activity^16^. Morphological analysis revealed a mild, yet statistically significant increase in the number of cerebellar neurons in male mice with the *RELN-COLBOS* variant. This supports the hypothesis of a GOF mechanism (Extended Data Fig. 4a,b), although the neuronal density phenotype was not observed in other brain regions (Supplementary Fig. 10).

**Extended Data Fig. 5:**
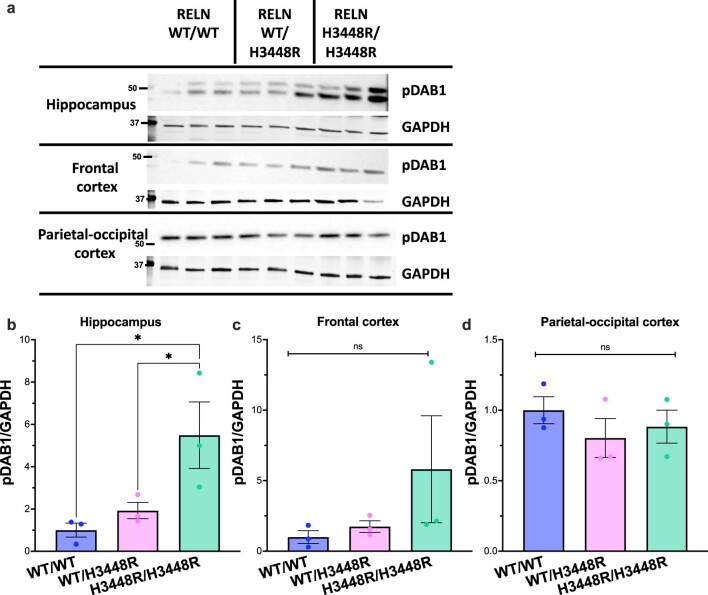
pDab1 levels in different brain regions of the novel H3448R-*RELN* transgenic mouse model. **a**, Representative blots of pDab1 and GAPDH levels in hippocampus, frontal cortex and parietal-occipital cortex of total homogenate obtained from *RELN* WT/WT, *RELN* WT/H3448R and *RELN* H3448R/H3448R brains (n = 3). **b-c**, Quantification of normalized to GAPDH levels of pDab1 positive bands detected in the hippocampus (**b**), frontal cortex (**c**), and parietal-occipital cortex (**d**) from RELN WT/WT, *RELN* WT/H3448R and *RELN* H3448R/H3448R male mice at 16 weeks of age, n = 3 mice. Data is showing that there are significantly increased pDab1 levels in the presence of the homozygous H3448R mutation compared to heterozygous and WT hippocampus (**b**, *p = 0.03866, WT/WT vs. WT/H3448R, n1 = n2 = 3, t = 3.32, DF = 6; *p = 0.01596, WT/WT vs. H3448R/H3448R, n1 = n2 = 3, t = 2.63773, DF = 6). For all quantification, we used one-way ANOVA, followed by Fisher’s Least Significant difference (LSD) test for multiple comparisons. All data is presented as average ± s. e. m. Source data

This mouse model allowed us to examine sexually dimorphic effects of the *RELN-COLBOS* variant, a feature that has been described for conditions linked to genetic variation in *RELN*, including schizophrenia, bipolar disease, autism and Alzheimer’s disease^17–22^. Increased Dab1 phosphorylation and enhanced oligomerization of RELN were observed only in male mice (Fig. 4a–c, Extended Data Fig. 5 and Supplementary Figs. 8 and 9). This finding was consistent with our observation of optimal association of *RELN-COLBOS* with protection against ADAD in a male versus a female case. Homozygosity was required to detect changes in Dab1 and in GSK3β activity (another downstream target of RELN signaling) associated with the *RELN-COLBOS* variant (Supplementary Figs. 11 and 12). Whether this homozygosity requirement is a species-specific effect or whether it depends on other factors such as age is to be examined. Altogether, these data indicate that *RELN*-H3447R is a GOF (hypermorph) variant.

To attempt to correlate the phenotypes of *RELN-COLBOS* in mice and humans, we used a crossbreeding strategy, using our knockin mouse model and a tauopathy mouse model, specifically the STOCK Tg(Prnp-MAPT*P301L)JNPL3Hlmc mouse from the Hutton’s laboratory, distributed by Taconic Biosciences. This mouse model expresses a mutation in the Tau gene, leading to accumulation of Tau tangles and neuronal loss in specific brain regions, commonly used to study tauopathies^23^. The decision to use this mouse model was based on the known effects of RELN signaling on Tau phosphorylation^24^ and our clinical observations of a relative reduction of tauopathy in certain brain regions from postmortem human brain samples of the protected case. Our study found that male P301L mice expressing the *RELN-COLBOS* allele had a significant reduction of human Tau phosphorylation (pTau205) in the hippocampus (HIC) (Fig. 4d,e) and medulla oblongata (MO) (Supplementary Fig. 13) compared to controls (Fig. 4d). We also observed that the abnormal limb-clasping response, which is a common consequence of tauopathy in mice, was significantly rescued in *RELN-COLBOS* mice with the Tau transgene (Fig. 4f,g). Although additional studies of this model are necessary, our findings strongly support our hypothesis that *RELN-COLBOS* is a GOF mutation and it is probably genetically implicated in the resilience to tauopathy.

**Fig. 5:**
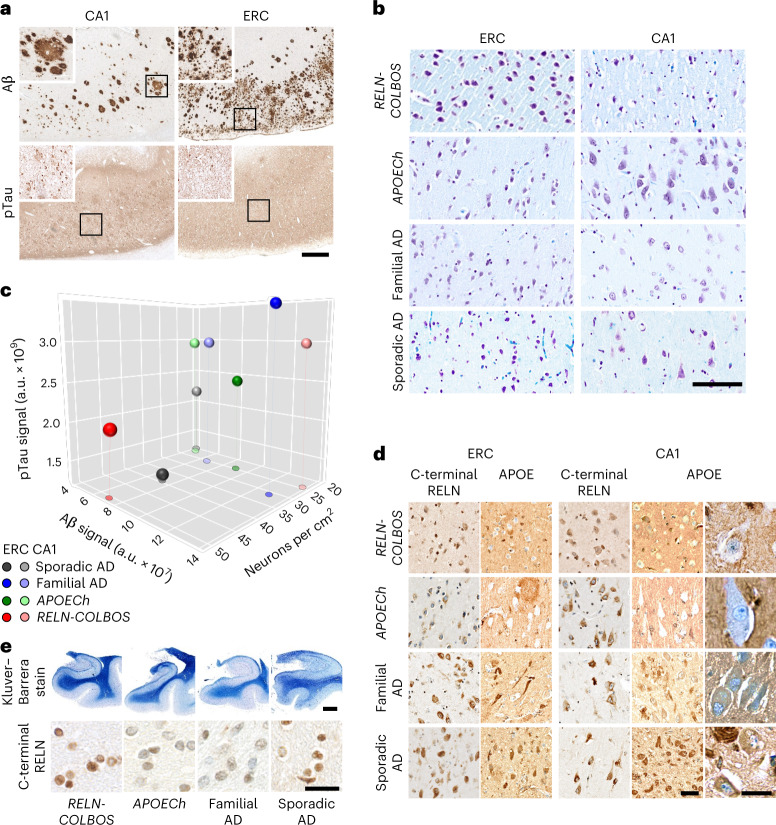
Neuropathological characterization of the case with *PSEN1*-E280A;*RELN-*H3447R. **a**, Aβ and pTau pathologies in the CA1 and ERC. Both pathologies present wide distribution and intensity. Aβ pathology shows diffuse plaques with varied distribution and size (panels and insets). pTau pathology shows varied density of neurofibrillary tangles and diffuse Tau pathology. Scale bar, 500 μm. **b**, Neurons stained with Klüver–Barrera stain in the CA1 and ERC of the case with *PSEN1*-E280A/*RELN-COLBOS*, the case with *PSEN1*-E280A/*APOECh*, a case with average-onset *PSEN1*-E280A familial AD and a case with sporadic AD. Scale bar, 125 μm. **c**, Three-dimensional scatter plot for Aβ, pTau and neuronal density for ERC and CA1 from cases with *RELN-COLBOS*, *APOECh*, familial AD (*n* = 5) and sporadic AD (*n* = 4). The ERC in the case with *RELN-COLBOS* shows the highest neuronal density, with low Aβ and pTau pathologies. **d**, C-terminal RELN and APOE staining of the cases with *RELN-COLBOS*, *APOECh*, familial AD and sporadic AD in the ERC and CA1. The case with *RELN-COLBOS* shows a stronger background signal in both structures with lower intraneuronal signal for C-terminal RELN in the ERC. Similarly, the case with *APOECh* shows lower intraneuronal signal in ERC with the C-terminal RELN antibody and very low intraneuronal signal in both structures with the APOE antibody (magnified right). Finally, APOE staining shows noticeable plaque- and tangle-like signals in cases with familial and sporadic AD in both structures, the ERC and CA1. Scale bars, 100 μm and 25 μm in the magnified panel. **e**, Klüver–Barrera staining of whole hippocampal and parahippocampal sections (top), together with representative magnified images of parahippocampal subcortical white matter stained with C-terminal RELN antibody in the cases with *RELN-COLBOS*, *APOECh*, familial and sporadic AD (bottom). The case with *RELN-COLBOS* showed increased white matter Luxol Fast Blue signal intensity, while the cases with *RELN-COLBOS* and sporadic AD showed increased intracellular C-terminal RELN signal in white matter. Scale bars, 2.5 mm for the top panel and 25 μm for the bottom panel.

**Fig. 6:**
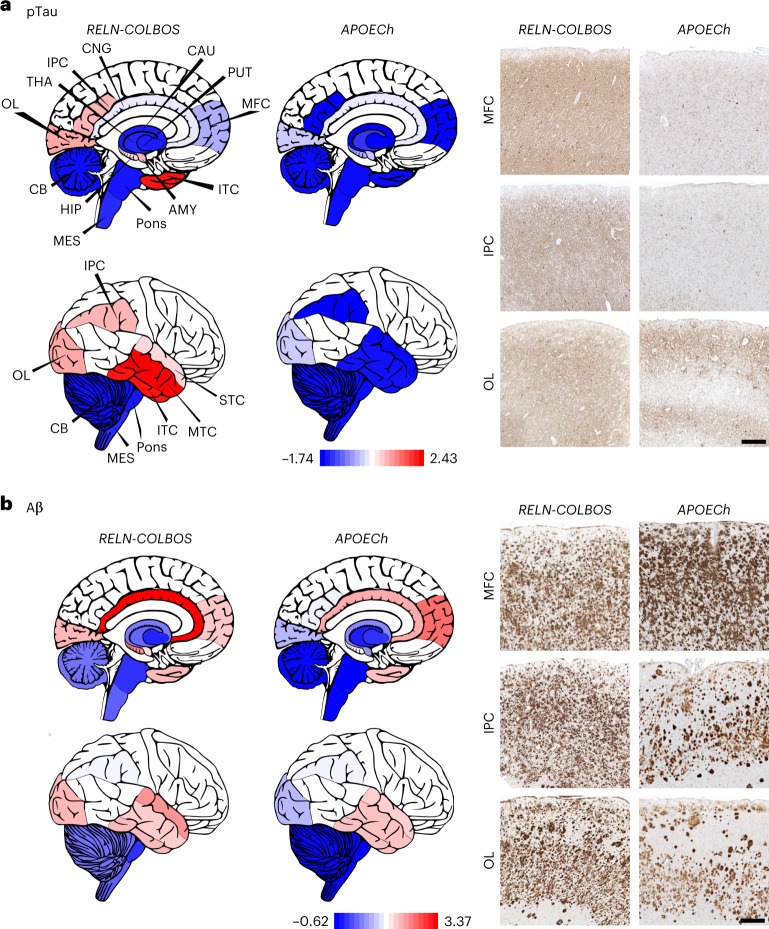
Brain distribution of AD hallmarks in the cases with *RELN-COLBOS* and *APOECh*. **a**,**b**, Graphic representation and representative images of the distribution and intensity of pTau (**a**) and Aβ (**b**) pathology signals with normalized minimum and maximum values shown in red and blue, respectively in the cases with *RELN-COLBOS* and *APOECh*. The case with *APOECh* showed distinct decreased pTau pathological profiles in all cortices compared to the case with *RELN-COLBOS*. Despite some distribution differences, the Aβ pathology profile was similarly severe in both cases. AMY, amygdala; CAU, caudate; CNG, cingulate cortex; IPC, inferior parietal cortex; ITC, inferior temporal cortex; MES, mesencephalon; MFC, medial frontal cortex; MTC, middle temporal cortex; OL, occipital lobe; PUT, putamen; STC, superior temporal cortex; THA, thalamus. Scale bars, 250 μm.

**Extended Data Fig. 6:**
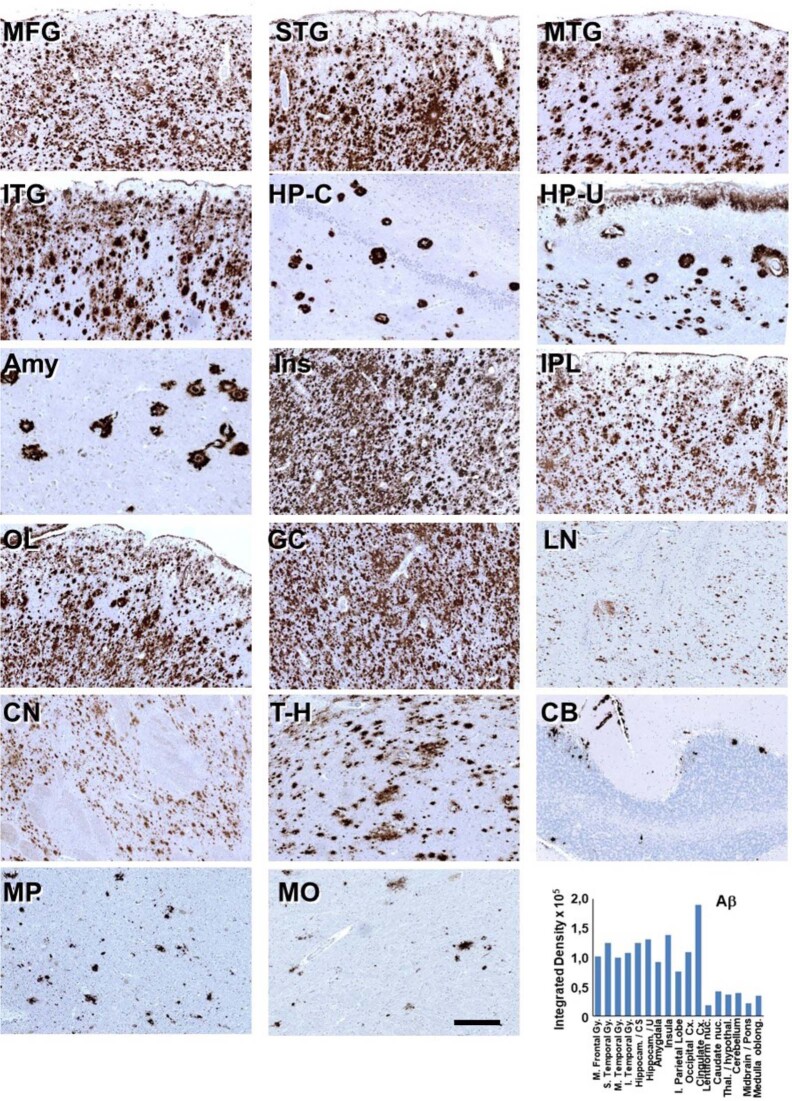
IHC staining for Amyloid β. MFG = Medial frontal gyrus, STG = Superior temporal gyrus, MTG = Medial temporal gyrus, ITG = Inferior temporal gyrus, HP-C = Hippocampus/collateral sulcus, HPUncus = Hippocampus/uncus, Amy = Amygdala, Ins = Insula, IPL = Inferior parietal lobe, OL = Occipital lobe, GC = Gyrus cinguli, LN = Lentiform nucleus, CN = Caudate nucleus, T-H = Thalamus/Hypothalamus, CB = Cerebellum, MP = Midbrain/pons, MO = Medulla oblongata. Scale bar = 100 𝜇m.

**Extended Data Fig. 7:**
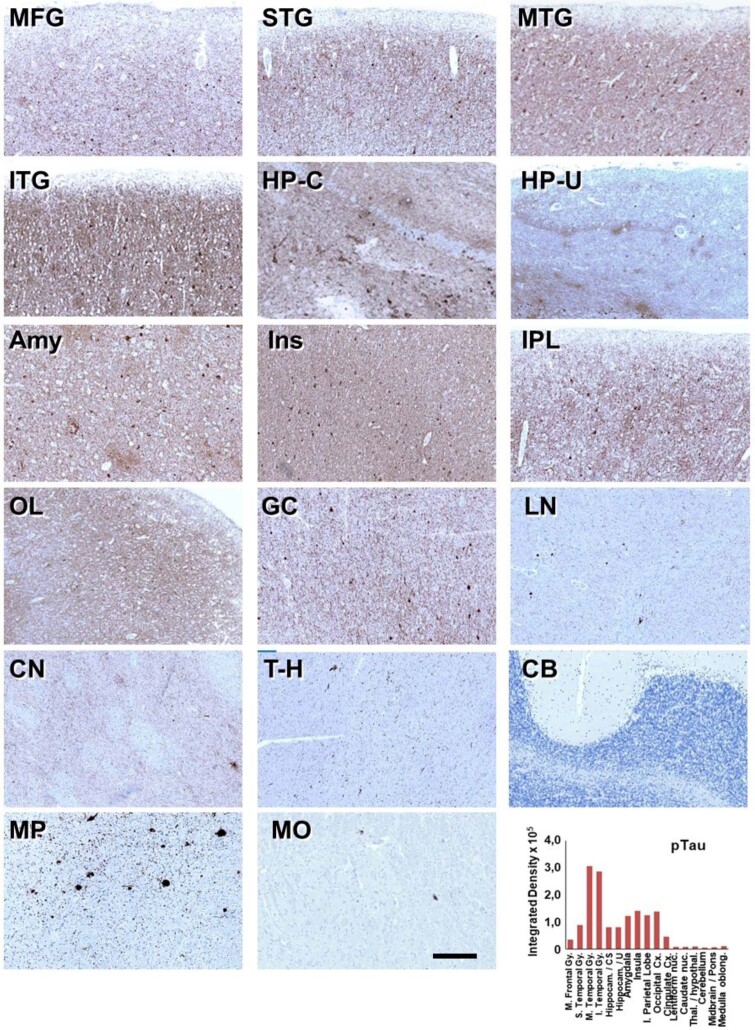
IHC staining for ptau. MFG = Medial frontal gyrus, STG = Superior temporal gyrus, MTG = Medial temporal gyrus, ITG = Inferior temporal gyrus, HP-C = Hippocampus / collateral sulcus, HP-Uncus = Hippocampus/uncus, Amy = Amygdala, Ins = Insula, IPL = Inferior parietal lobe, OL = Occipital lobe, GC = Gyrus cinguli, L = Lentiform nucleus, CN = Caudate nucleus, T-H = Thalamus/Hypothalamus, CB = Cerebellum, MP = Midbrain/pons, MO = Medulla oblongata. Scale bar = 100 𝜇m.

**Extended Data Fig. 8:**
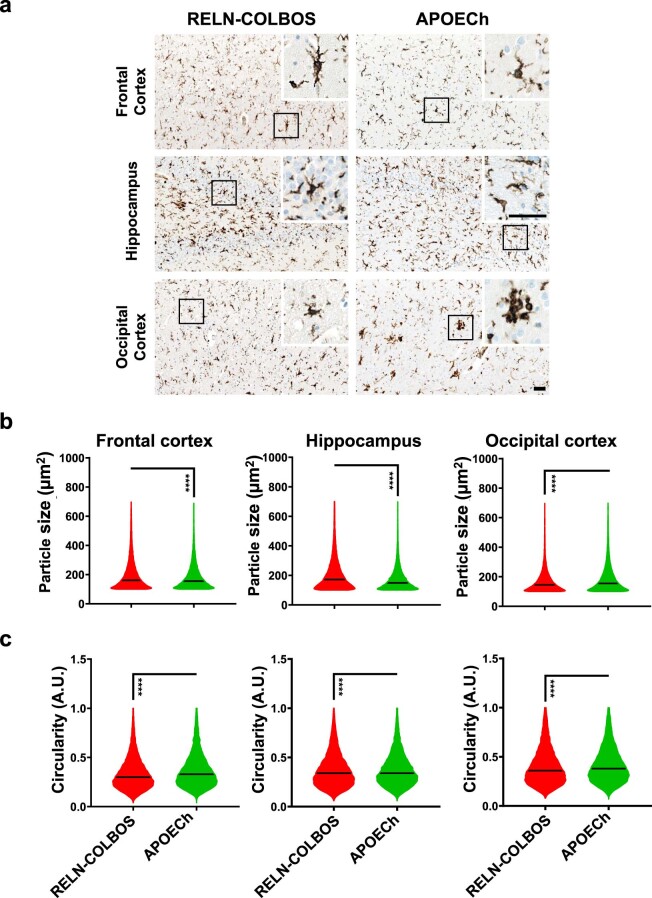
Morphological assessment of microglia in *RELN-COLBOS* and *APOECh* protected cases. **a**, Representative pictures of ionized calcium binding adaptor molecule1 (IBA1) stained microglia in frontal cortex, hippocampus, and occipital cortex from both, *PSEN1* E280A *RELN-COLBOS* case and the *PSEN1* E280A *APOECh* homozygous case. Bar, 50 μm. **b, c**, Violin plots for size and circularity assessment in *RELN-COLBOS* and *APOECh* cases. The *RELN-COLBOS* showed significantly larger microglia in frontal cortex (FC, number of particles, np = 45679) and hippocampus (Hip, np = 21407), and smaller microglia in the occipital cortex (OC, np = 22365). Microglial circularity was higher in the *APOECh* case in all areas (np values for *APOECh:* FC = 18537, Hip = 14265, OC = 23845). P ≤ 0.001 ****. Two-sided Student’s test was used for analysis.

Postmortem examination of the case with *RELN-COLBOS* indicated neuropathological evidence of severe AD (brain weight = 745.4 g, classified as CERAD C, Braak VI stage and Thal phase 5) with extensive amyloid and Tau pathology, while showing some hippocampal formation-specific findings (Fig. 5, Extended Data Figs. 6 and 7, Supplementary Figs. 14–16 and extended description in the supplementary results). Recently, we reported the neuropathological profile of the *PSEN1*-E280A carrier homozygous for the *APOECh* mutation. The Christchurch case showed a unique pathological phenotype among cases with *PSEN1*-E280A, with remarkably low pTau pathology in most brain regions except in the visual primary cortex^25^. In contrast, side-by-side comparisons showed that the case with *RELN-COLBOS* had more pTau pathology relative to the case with *APOECh* except in specific regions (Fig. 6a and Supplementary Fig. 17). Both cases showed extensive Aβ pathology in all evaluated areas, albeit with some individual differences (Fig. 6b and Supplementary Fig. 17). Microglial morphological assessment of the protected cases indicated that the *APOECh* microglia were significantly more active in the studied areas (Extended Data Fig. 8).

**Extended Data Fig. 9:**
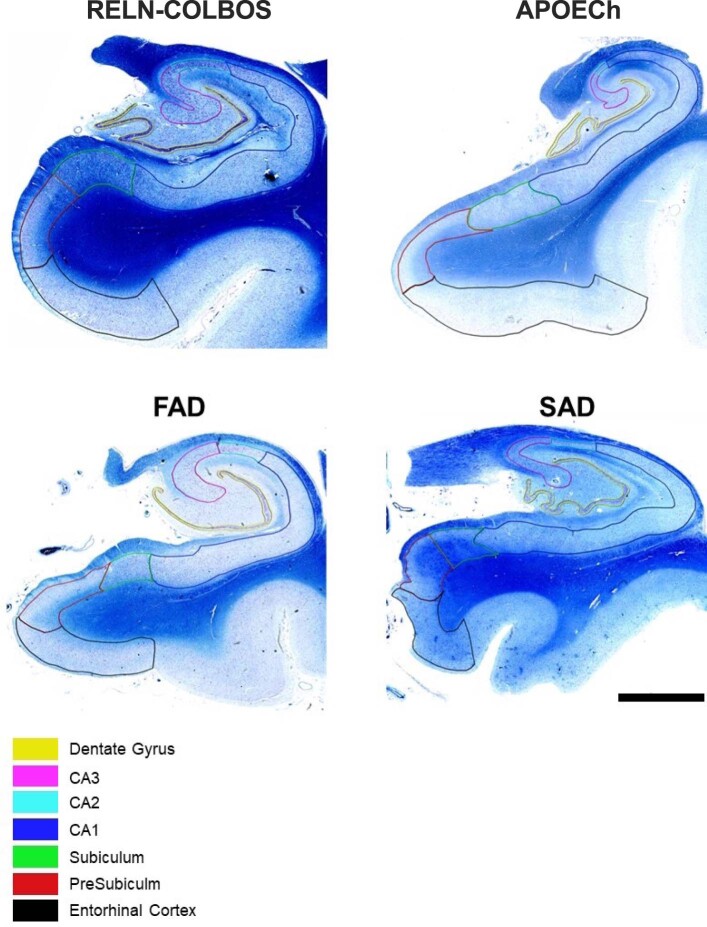
Klüver Barrera staining of the *RELN-COLBOS* case, the *APOECh* case, a representative *PSEN1* E280A FAD case and a representative SAD case. Klüver-Barrera staining using luxol fast blue staining for myelin and cresyl violet staining for neuronal perikaryal in selected brain regions. Code-colored lines, regions of interest for neuronal density measurements in *Cornu Ammonis* (CA) structures CA1, CA2, and CA3, together with the Dentate Gyrus, *Subiculum*, *Presubiculum* and Entorhinal cortex. Scale bar, 3 mm.

**Extended Data Fig. 10:**
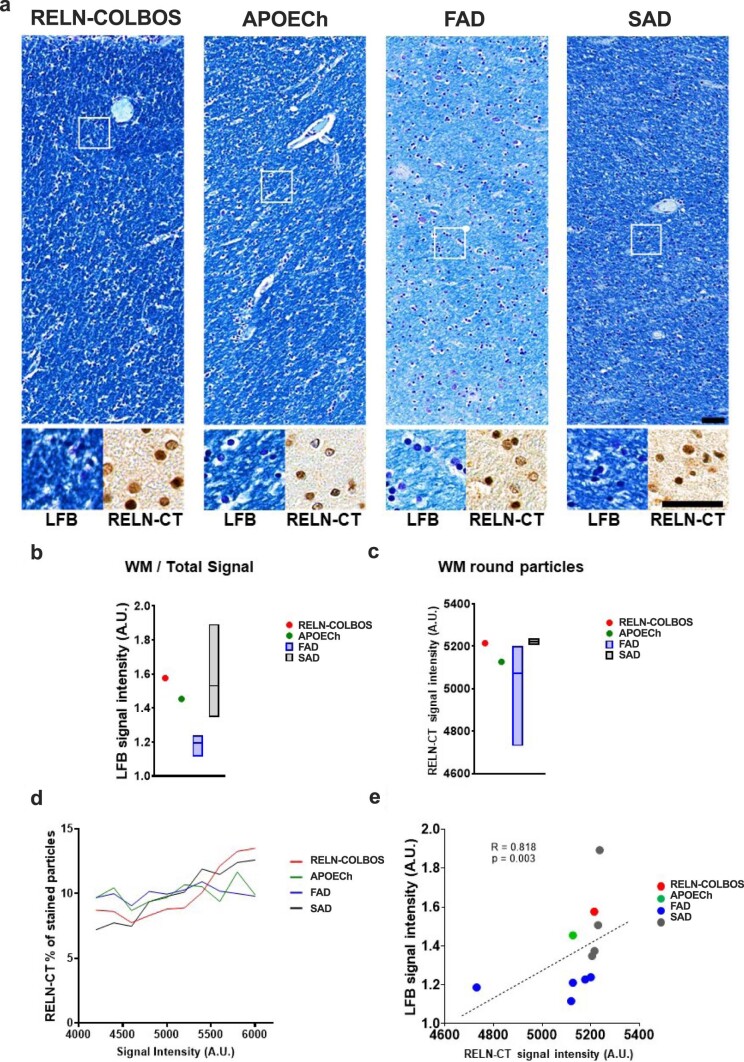
Analysis of the white matter signal in the *RELN-COLBOS* in comparisons to other AD cases. **a**, Representative images for luxol fast blue (LFB) staining (large panel and small left panels) and RELN-CT (small right panel) of the sub hippocampal white matter of the *RELN-COLBOS* case, the *APOECh* case, a representative *PSEN1* E280A FAD case and a representative SAD case. Scale bars, 50 𝜇m. **b**, Bar graph for normalized measurement of white matter relative to total LFB signal of the *RELN-COLBOS* case, the *APOECh* case, *PSEN1* E280A FAD cases (n = 5) and SAD cases (n = 4). Both the *RELN-COLBOS* case and the *APOECh* case show relative higher white matter signal than the other FAD cases. **c**, Bar graph for the quantification of RELN-CT signal in round particles in the sub-hippocampal white matter of the *RELN-COLBOS* case, the *APOECh* case, *PSEN1* E280A FAD cases (n = 5) and SAD cases (n = 4). The *RELN-COLBOS* case shows higher RELN-CT signal intensity than all FAD cases, including the *APOECh* one. **d**, Line graph for RELN-CT positive round particles signal intensity distribution in the *RELN-COLBOS* case, the *APOECh* case, *PSEN1* E280A FAD cases (n = 5) and SAD cases (n = 4). The *RELN-COLBOS* case showed a larger percentage of particles with higher RELN-CT signal intensity when compared with the other AD cases. **e**, Scatter plot of Spearman’s Rho correlation analysis for LFB signal intensity and RELN-CT round particles signal intensity in all analyzed AD cases. A statistically significant positive correlation was identified (p = 0.003, R = 0.818).

We focused our analysis on the HIC and associated cortices because these structures are known to be affected early in AD^26^. Neurons in layer II of the ERC and ERC neurons in general are particularly vulnerable to aging and AD^27^. We measured neuronal density in hippocampal and parahippocampal areas of the cases with *RELN-COLBOS*, the case with AD-resistant *APOECh*, cases with typical *PSEN1*-E280A and cases with typical sporadic AD (Fig. 5b, Extended Data Figs. 9 and 10, Supplementary Figs. 18 and 19 and Supplementary Table 10). We found that lower AD pathology was associated with high neuronal density in the ERC of the case with *RELN-COLBOS* compared to the case with *APOECh* or the controls with familial and sporadic AD (Fig. 5c).

This association was not apparent in other subregions such as the CA1 (Fig. 5c and Supplementary Figs. 18 and 19). The cases with *RELN-COLBOS* and *APOECh* showed distinctively lower intraneuronal APOE signal than the controls with familial and sporadic AD (Fig. 5d and Supplementary Table 10) whereas the case with *RELN-COLBOS* showed higher Reelin intracellular signal in the white matter (Fig. 5e and Extended Data Fig. 10).

Neuropathological findings were consistent with our in vivo neuroimaging observations and confirmed a potential role for the ERC as a target of *RELN*-mediated mechanisms critical for the resilience to ADAD.

## Discussion

We characterized a male heterozygous for the *RELN-COLBOS* variant who was resilient to the cognitive impairment associated with the *PSEN1*-E280A mutation. The observation of low Tau pathology and increased neuronal density in the entorhinal cortex compared to other cases with AD implicates this brain region in *RELN*-mediated mechanisms relevant to protection against AD (Supplementary Table 1 and Figs. 1 and 5b,c). Because the comparative neuropathology was conducted in a relatively low number of cases, the results should not be considered definitive and they are only helpful as informative to generate hypotheses.

A female sibling carrier of the *RELN-COLBOS* and *PSEN1*-E280A variants also presented with delayed age at onset of cognitive decline, although with less optimal protection compared to her brother and prolonged end-stage disease. The female sibling had a history of a severe head injury, which required reconstructive surgery, as well as a history of depression and hypothyroidism. These factors may have had an impact on her clinical profile and should be taken into consideration when evaluating her phenotype^2^. In addition, *RELN*-specific sexual dimorphism may have contributed to her distinct features. We cannot rule out the possibility that other factors, including additional variants, may have contributed to the AD resilience phenotype in *RELN-COLBOS* carriers. Notwithstanding these potential limitations, others identified *RELN* as a candidate gene associated with AD pathology in cognitively healthy individuals^28^ and *DAB1* variants were linked to AD risk in *APOE4* homozygotes, further linking the *RELN*/*DAB1* pathway to Alzheimer’s^29^.

We previously reported a female case homozygous for *APOECh* who was resistant to ADAD-related dementia, had widespread amyloid pathology and low Tau pathology in the ERC^2^. Tauopathy was more extensive in the case with *RELN-COLBOS* compared to the *APOECh* homozygote, except for the ERC, which was largely spared in both, suggesting resilience in the case with *RELN-COLBOS*.

The hypermorphic effect of *RELN* is mild. This is the first known report of a *RELN* hypermorph and a stronger effect may not support proper development in this critical signaling process. The experimental evidence of a GOF mechanism for the *RELN-COLBOS* variant, and the fact that a patient with extreme protection against ADAD has it, establish a rationale for genetic implication in the observed phenotypes. Our hypothesis is that *RELN-COLBOS* is not a neutral variant and probably contributes to the phenotype of the individual. It is possible that other genetic variants may also contribute; if so, these variants and their effects will have to be interpreted and compared to the effect of *RELN-COLBOS* using the data we have provided.

The *APOECh* mutation impairs APOE binding to GAG and the APOE receptors^2,30^. Conversely, the *RELN-COLBOS* variant enhances RELN binding to GAG and NRP1, possibly giving it a competitive advantage for binding to its receptors^7^. Interactions with GAG-containing proteins like heparan sulfate proteoglycans may enhance local concentration of RELN and amplify its signaling effect. Our analyses of the case with *RELN-COLBOS* revealed a mechanism potentially linking RELN interactions via GAG or other receptors to the protection against AD. Regulation of this RELN-protective pathway, particularly in the ERC, may have a profound therapeutic impact on the resistance to Tau pathology and neurodegeneration, and resilience against cognitive decline and dementia in AD.

## Methods

### Clinical assessment

This research complies with all relevant ethical regulations. Written informed consent was obtained from all participants and the local institutional review boards for human research approved the study. This includes the institutional human research ethics committee of the University of Antioquia in Colombia and the Partners Human Research Committee in Boston.

### In vivo neuroimaging

We used PiB and FTP PET to image cerebral Aβ and Tau burden, respectively. Structural MRI and both PET scans were conducted at MGH. The FDG PET was performed at the University of Antioquia and the procedures and data analyses were performed as described previously^2^. Briefly, MRI images were processed with FreeSurfer (v.6.0) to identify surface boundaries and standard ROIs. PET data were acquired and processed according to previously published protocols^31,32^, whereby PiB data were expressed as DVR (Logan, 0–60 min) and FTP as SUVR (80–100 min), both using cerebellar gray matter as the reference region. PET images were affine coregistered to each individual’s T1 images and visualized using FreeSurfer surface projections (sampled at the gray matter midpoint, surface-smoothed 8 mm). No partial volume correction was applied to PET images for the purposes of this study.

### Genetic and molecular studies

We conducted whole-exome (WES), WGS and Genomizer (v.10.10) analysis of the individual to obtain a ranking of AD-related potential risk factors as shown previously^2^ and as described in detail in the additional genome sequencing section of the Supplementary Information.

### Cell culture

Plasmid encoding for the full-length murine recombinant RELN was a gift from T. Curran via Addgene (plasmid no. 122444 (ref. ^14^)). The plasmid was subsequently mutagenized to obtain the H3448R mutation homologous of human *RELN*-H3447R as fee-for-service by Custom DNA Constructs. Primary CD1 brain cortex mouse neurons (catalog no. M-CX-400, Lonza) were cultured in neurobasal medium (Gibco) supplemented with B-27 (Thermo Fisher Scientific), glutaMAX (Gibco) and normocin (Invivogen). Cells were plated on poly-L-lysine-coated (Sigma-Aldrich) wells and treated on day 6 after liquid nitrogen recovery. Treatments with recombinant RELN (WT RELN or *RELN*-H3448R, 4 µg ml^−1^, produced by Innovagen) were incubated for either 5 min or 1 h at 37 °C, 5% CO_2_ in the presence of 10 µM MG-132 proteasome inhibitor (catalog no. ab141003, Abcam). Cells were washed in ice-cold PBS (Gibco) and lysed in radioimmunoprecipitation assay (RIPA) (catalog no. 9806, Cell Signaling Technology) supplemented with 10 µM Mg-132, Triton-X100 (Sigma-Aldrich), cOmplete, Mini, EDTA-free protease inhibitor cocktail (catalog no. 4693159001, Merck Millipore) and phosphatase inhibitors (catalog no. 4906837001, Sigma-Aldrich; catalog no. P0044, Merck Millipore). Protein concentration was determined by Pierce bicinchoninic acid (BCA) protein assay kit (catalog no. 23227, Thermo Fisher Scientific) according to the manufacturer’s instructions. Samples were prepared containing 10 µl Laemmli buffer (Boston Bioproducts) and 4 µl of 1 M dithiothreitol (DTT) (Sigma-Aldrich) and diluted to a final volume of 40 µl with water and denatured 5 min at 90 °C.

### Western blotting

A total of 20 µg of cell lysates were prepared in 4 µl of 1 M DTT and 10 µl Laemmli buffer to a final volume of 40 µl and denatured with heat for 5 min at 90 °C. Samples were separated electrophoretically for 1 h at 90 V using 4–20% precast gradient gels (Mini-PROTEAN TGX, Bio-Rad Laboratories) and Tris-Glycine-SDS buffer (Bio-Rad Laboratories). Proteins were transferred to 0.45-μm nitrocellulose membranes for 1 h at 90 V in ice-cold 20% Tris-glycine-methanol buffer (Bio-Rad Laboratories). To detect pDab1 levels, proteins were transferred onto polyvinylidene fluoride membranes using the iBlot 2 dry transfer system (catalog no. IB21002S, Thermo Fisher Scientific). Total protein levels were detected using LI-COR membranes were blocked for 1 h with Odyssey Blocking Buffer (LI-COR Biosciences) or for 2 h with 5% nonfat dry milk (catalog no. M17200-100.0, Research Products International); both protease and phosphatase inhibitor cocktails where blocked for anti-pDab1 western blotting. β-Tubulin (ms host; 1:2,000 dilution, catalog no. 86298S, Cell Signaling Technology), anti-pDab1 (recombinant; 1:7,500 dilution, catalog no. MBS8511213, MyBiorsorce), anti-pGSK3β-Ser9 (1:1,000 dilution, catalog no. D85E12, Cell Signaling Technology), anti-pGSK3β-Tyr216/279 (1:1,000 dilution, catalog no. 05-413, Merck Millipore), anti-GSK3β (1,1,000 dilution, catalog no. 5558, Cell Signaling Technology), anti-GAPDH (1:5,000 dilution, catalog no. ab8245, Abcam) and anti-RELN (ms host, 1:1,000 dilution, clone CR-50, catalog no. D223-3, MBL) were used as primary antibodies and incubated in blocking buffer for either 2 h at room temperature or 18 h at 4 °C. After washing the blots three times with Tris-buffered saline with Tween 20 buffer (Pierce, Thermo Fisher Scientific); secondary antibodies were incubated either 1 h or 45 min at room temperature (IRDye 800CW donkey anti-mouse, catalog no. 925-32212 or IRDye 680CW donkey anti-rabbit; 1:10,000 dilution, catalog no. 925-68073, LI-COR Biosciences). Immunoreactive bands were detected using the Odyssey Infrared Imaging System and visualized on the Image Studio Acquisition Software (v.2.1, LI-COR Biosciences). Detection of pDab1 was obtained with anti-rabbit-horseradish peroxidase-conjugated antibody (catalog no. HAF008, R&D Systems) followed by a 5-min incubation with SuperSignal West Pico PLUS Chemiluminescent Substrate and acquisition on the Syngene G:BOX digital ECL detection system using the Genesys (v.1.5.3.0) software. Validation of DAB1-positive bands was conducted via immunoprecipitation (Supplementary Fig. 3) and mass spectrometry (Supplementary Fig. 4). All other western blot images were acquired using the Odyssey Infrared Imaging System and visualized with Image Studio v.2.1 (LI-COR Biosciences), PowerPoint 365 for macOS (v.16.69.1), Prism 9 (v.9.4.1) (GraphPad Software) and ImageJ v.2.3.0/1.53q. The blots are presented in Figs. 3a and 4a. Supplementary Figs. 10 and 11 were acquired using Genesys v.1.5.3.0.

### Heparin-sepharose affinity chromatography

We tested changes in binding to heparin of *RELN* variants chromatographically using an optimized version of a protocol previously published by our laboratory^2^. Briefly, after equilibration of the heparin column (catalog no. 6554-1, BioVision) at room temperature, columns were washed with five volumes of degassed 20 mM Tris-HCl buffer (pH 7.5). Recombinant C-terminal RELN peptides were produced and purified as fee-for-service by Innovagen: WT RKQNYMMNFSRQHGLRHFYNRRRRSLRRYP and H3447R RKQNYMMNFSRQHGLRRFYNRRRRSLRRYP. All synthetic peptides listed in Fig. 3 were also produced by Innovagen. One milliliter of 50 μg ml^−1^ peptide (H3447 or WT and H3447R) was recycled through the column five times; the last flow through was collected for further analysis. The column was washed five times with the same buffer and the protein was eluted using a 0.05 M step gradient of NaCl in 20 mM Tris-HCl (0–1 M, 1 ml per fraction). To ensure the complete release of the protein, the column was washed with 5 M NaCl 20 mM Tris-HCl. Three independent experiments were conducted for C-terminal WT RELN and H3447R. All eluted fractions were analyzed spectroscopically by reading the absorbance at 280 nm with a NanoDrop 2000 Spectrophotometer. Blank-corrected fractions were subsequently analyzed with Prism 8.

### HPLC

Fifty microliters of 0.3 µg µl^−1^ Reelin peptides were injected at 0.3 ml min^−1^ into a POROS Heparin 50 µm Column (4.6 × 50 mm, 0.8 ml; Thermo Fisher Scientific) using 10 mM PBS and 0.15 M KCl as the mobile phase using a Shimadzu SCL-40 instrument. Loading of the sample to the column was performed for 5 min. Affinity of the samples to heparin was tested using the following gradient steps: 0–13.5 min: at 0.15 M KCl to load the sample; 13.5–14.5 min: at isocratic conditions at 0.5 M KCl; 14.5–24.5 min: at a gradient of 0.5–1 M KCl (ramp); 24.5–45 min: at 1 M KCl (isocratic elution); 45–55 min: 1 M KCl at 0.6 ml min^−1^ (wash); 55.0–56.0 min: ramp to 0.15 M KCl at 0.3 ml min^−1^; 56.0–59.0 min: 0.15 M KCl (reset column). Fluorescence intensities were measured at an excitation wavelength of 260 nm and emission wavelength of 290 nm, based on the fluorescence properties of aromatic amino acids. Retention times were analyzed with LabSolutions v.5.106. Chromatogram intensities were normalized to the maximum peak intensity in Prism 9.

### BLI

We used Fc-fusion Reelin CTR peptides because peptides alone would be smaller than the limit of detection in this experimental design. The fusion proteins were obtained by cloning DNA sequences encoding amino acids 3,429–3,460 of RELN-CTR into the pFUSEN-hG2Fc plasmid from InvivoGen. To assess the heparin-Fc-Reelin interaction, the Octet system was used to assess heparin-protein kinetics as a fee-for-service by Precision Antibody. A total of 50 µg ml^−1^ biotinylated heparin (catalog no. 375054, Merck Millipore) was immobilized on the biosensor tip surface for 300 s on preconditioned biosensors. This was followed by quenching with biocytin at 50 µg ml^−1^, baseline buffer dilution for 120 s, 200 nM of analyte (Fc-fusion proteins) for 120 s and disassociation in assay buffer for 120 s. BLI was additionally used to assess NRP1 protein kinetics at 30 °C and 1,000 rpm agitation; 1 mg ml^−1^ NRP1 (catalog no. 3870-N1-025, R&D Systems) was biotinylated at a 1:2 molar ratio, desalted and immobilized on the streptavidin biosensor tip (Pall ForteBio) surface. This was followed by (1) 180 s of baseline buffer dilution, (2) loading of the ligand (NRP1), (3) 240 s association (analyte) and (4) 300 s disassociation in assay buffer. The assay buffer consisted of an SD Buffer (PBS, 0.05% Tween 20, 0.01% BSA, pH 7.4). The experimental data were fitted with the 1:1 binding model and analyzed with global fitting using the Octet Data Analysis software (v.9) to calculate KD.

### ITC

Heparin and peptides were dissolved in Dulbecco’s PBS buffer (pH 7.22) and spun for 5 min at 10,000 rpm. Then, 2 μl of the 500 μM peptide was successively injected into the cell containing 5 μM heparin at 25 °C with 180 s between injections and at 1,000 rpm stirring speed (MicroCal iTC200). Data were evaluated using the MicroCal iTC200 Evaluation software (Malvern).

### Mouse model and in vivo analyses

We generated the *RELN*-H3448R-Tg knockin mouse model carrying the *RELN-COLBOS* variant via homologous recombination as fee-for-service (Taconic-Cyagen) by introducing the H3448R (CAC>CGT) mutation into exon 64 in the 3′ homology arm of the *RELN* gene. Mouse and human *RELN* have very high homology with 95.2% identity. Human *RELN* is missing a valine residue at position 15 resulting in it having 3,460 instead of 3,461 amino acids like its mouse counterpart. Structurally, the two proteins have similar domain structure consisting of a signal peptide, an F-spondin-like domain, eight Reelin repeats (RR1–8) and a positively charged sequence at the C terminus. The last 105 amino acids including the region impacted by *RELN-COLBOS* are identical between mice and human. Gene targeting was obtained using C57BL/6 embryonic stem (ES) cells. Knockin mice were generated by injecting targeted ES cells into blastocysts that were introduced into the foster mothers used to generate the mouse crossings (Supplementary Fig. 7). Mice were killed by placing them in chambers saturated with CO_2_. HICs, midbrains, frontal cortices, parieto-occipital regions and CBs were dissected and stored at −80 °C upon cervical dislocation, ensuring a postmortem interval of less than 3 min. Brain homogenates were obtained in modified RIPA buffer (Cell Signaling Technology) supplemented with protease (Roche) and phosphatase (Sigma-Aldrich) inhibitors, using a tissue homogenizer (two times, 15 s pulses). Homogenized tissue was then vortexed for 20 s every 10 min for 1 h and centrifuged for 10 min at 10,000 rpm and 4 °C. The soluble protein fraction was then analyzed using a BCA assay (Pierce). Using western blotting, we measured the levels of RELN (clone CR-50), Dab1 (clone G-5, catalog no. sc-271136, Santa Cruz Biotechnology), pDab1 (Tyr232) in the CB of adult male and female mice (6–12 months, *n* = 3 mice for 6-month-old (m.o.) and 12-m.o. homozygous, *n* = 4 mice for 12-m.o. WT and heterozygous) either WT, heterozygous or homozygous for the *RELN*-H3448R mutation. This knockin model was crossed with the Tau P301L model from Taconic Biosciences. Littermates were included for analyses throughout. Additional details about mouse model design and clone selection are reported in Supplementary Table 9 and Supplementary Fig. 7. Eighteen-month-old WT and *RELN-COLBOS* knockin mice, human Tau P301L transgenic mice and crossbred Tau P301L/*RELN-COLBOS* mice were tested for the behavioral analyses and their brains were collected and prepared for morphological and immunohistochemical studies (Cresyl violet, Klüver–Barrera and pTau T205 IHC, 1:10,000; catalog no. EPR23505-13, Abcam). Animal study protocols were approved by the Schepens Eye Research Institute and the Institutional Animal Care and Use Committee.

### Behavioral studies

We used limb-clasping scoring to assess motor deficits in mice according to a previously published protocol^23^. Briefly, we habituated the animals to the user for 3 days and assessed the escape response when we elevated each mouse by the tail to promote the limb-clasping reflex while standing on a metal grid. We scored from 0 (observed legs in crossed position) to 2 (observed complete opening of the hind limbs).

### Neuropathology characterization

The postmortem interval was 210 min after death. The brain presented frontal lobe-predominant atrophy; the weight of the brain and associated structures was 745.4 g and the interuncal distance was 2.3 cm. After 5 days of fixation in 4% paraformaldehyde and sample preparation in paraffin, 3-μm-thick sections from the medial frontal gyrus, superior temporal gyrus, medial temporal gyrus, inferior temporal gyrus, HIC/collateral sulcus, HIC/uncus, AMY, insula, inferior parietal lobe, OL, cingulate gyrus, lentiform nucleus, caudate nucleus, thalamus/hypothalamus, CB, midbrain/pons and MO were cut, deparaffinized and stained with H&E or processed for IHC staining for Aβ (1:100; BAM-10, catalog no. Mob410, DBS Emergo Europe), pTau (1:100; AT8, catalog no. MN1020, Thermo Fisher Scientific), ionized calcium binding adapter molecule 1 (Iba1) (1:500; catalog no. 019-19741, Wako), glial fibrillary acidic protein (1:200; catalog no. M0761, DAKO), C-terminal Reelin (1:200; clone E-5, catalog no. sc-25346, Santa Cruz Biotechnology) and APOE (1:100; goat polyclonal, catalog no. AB947, Merck Millipore) and specific secondary antibodies: anti-mouse and anti-rabbit (P0260 and P0447, respectively, DAKO); and anti-neuronal nuclei antibody (clone A60; ms host, 1:100; catalog no. MAB377, Merck Millipore). Visualization was achieved with 3,3′-diaminobenzidine (DAB) (Ventana, Roche) and the Ultraview Universal Detection Kit (Roche) according to the manufacturer’s instructions. Automatic immunostaining was performed with a Ventana BenchMark XT system (Roche) according to the manufacturer’s instructions. Selected brain areas were also stained with Luxol Fast Blue (for myelin staining) and Klüver–Barrera staining. Cresyl violet staining was used for neuronal perikarya. The neuropathological workup was performed by experienced morphologists blinded to the origin of the sample (M.G. and D.S-F.). Sections were scanned using a Hamamatsu NanoZoomer automatic digital slide scanner (Hamamatsu Photonics) and obtained images and ROIs (cortex for cortical areas, whole stained sections for non-cortical areas) at a resolution of at least 1 px μm^−1^. Signal intensity, together with particles and total area, were assessed after performing color deconvolution and thresholding in the brown (DAB) color channel using ImageJ (v.1.52p, NIH)^33^. Neuronal counting was performed manually and normalized by area in selected ROIs in the hippocampal and parahippocampal structures. Information on the statistical analysis is reported in the dedicated section.

### Statistical analysis

All data presented are expressed as averaged values and errors are expressed either as s.e.m. or s.d. Statistical analyses were performed using Prism 8 or 9. *P* < 0.05 and an α of 0.05 were considered statistically significant. We used a Kruskal–Wallis test with Dunn post hoc analysis for multiple comparisons of four independent experiments to compare changes between primary cortical neurons treated with either mock, WT RELN or *RELN*-H3448R and presented data as the mean ± s.e.m. For the SPR data (Fig. 3c and Supplementary Fig. 4), we verified the accuracy of the results with a chi-squared test and compared the sensorgrams obtained experimentally with the sensorgrams generated mathematically by the BIAnalysis software (black line). Values ranging from 1 to 2 were interpreted as significant (accurate), and those below 1 as highly significant (highly accurate). Western blot analyses or neuronal counting of Cresyl violet-stained slides were done using a one-way ANOVA followed by Fisher’s least significant difference test for multiple comparisons using Prism 9 (v.9.4.1) as reported in the figure legends. The neuropathological data (Fig. 2) were analyzed and graphs were generated with Prism 8 (v.8.1.1) and the R (v.3.6.3) statistical software (R Foundation for Statistical Computing). Distribution and correlation analyses were performed using a Spearman correlation test. The brain color maps were created with the cerebroViz package for R. The statistical significance of all analyses was determined with *P ≤ 0.05, **P ≤ 0.01 and ***P ≤ 0.001. Statistical comparisons of two groups were performed using a two-sided Student’s *t*-test.

### Inclusion and ethics in global research

The study received institutional review board approval from the MGH, the Mass Eye and Ear and the local ethics committee at the School of Medicine at the Universidad the Antioquia, Medellín, Colombia. This work involved a collaboration between scientists in multiple countries including Colombia, the United States of America and Germany. Contributors from all sites are included as coauthors or in acknowledgements according to their contributions. Researchers residing in Colombia have been involved in study design, study implementation, data ownership and intellectual property as appropriate. The research is locally relevant due to the high prevalence of ADAD. Roles and responsibilities were agreed among collaborators ahead of the research. Local ethics committees approved all research involving human participants. To prevent any stigmatization, any and all identifying information has been removed to preserve the privacy of individuals. The Colombian team has retained ownership of any and all human biological material shared for research purposes.

### Reporting summary

Further information on research design is available in the Nature Portfolio Reporting Summary linked to this article.

## Online content

Any methods, additional references, Nature Portfolio reporting summaries, source data, extended data, supplementary information, acknowledgements, peer review information; details of author contributions and competing interests; and statements of data and code availability are available at 10.1038/s41591-023-02318-3.

## Supplementary information

Supplementary Information Supplementary Results, Figs. 1–19, Tables 1–10, Methods, References and unprocessed blots for the supplementary figures.

Reporting Summary

Supplementary Video 1 The 20 lowest-energy structures produced by NMR shown sequentially. Each of the 20 structures were shown for 5 frames each at a rate of 75 frames per second (FPS).

Supplementary Video 2 The 20 lowest-energy structures produced by NMR shown in rotation. The 20 lowest-energy structures shown in *x* axis and *y* axis rotation at 40 FPS.

Supplementary Video 3 The 20 lowest-energy structures produced by NMR shown layered on top of previously shown structures. Each structure was added to the previous structures for 5 s at a rate of 15 FPS.

## Data Availability

Anonymized clinical, genetic and imaging data are available upon request during working hours, subject to an internal review by F.L., J.F.A.-V. and Y.T.Q. to ensure that participant confidentiality and *PSEN1*-E280A carrier or noncarrier status are protected, completion of a data sharing agreement and in accordance with the University of Antioquia’s and MGH’s institutional review board and institutional guidelines. Experimental data are available upon request, subject to the MGH and Schepens Eye Research Institute of Mass, Eye and Ear institutional guidelines. Material and data requests will be considered based on a proposal review, completion of a material transfer agreement or a data use agreement (or both) and in accordance with the MGH and Schepens Eye Research Institute of Mass, Eye and Ear institutional guidelines. Please submit requests for participant-related clinical and imaging data and samples to Y.T.Q. (yquiroz@mgh.harvard.edu); requests for experimental data and materials, genetic and scRNA-seq data should be sent to J.F.A.-V. (joseph_arboleda@meei.harvard.edu); and requests for neuropathology specimens should be forwarded to F.L. (francisco.lopera@gna.org.co). The RELN-COLBOS mouse model will be made freely available to the community via the Mutant Mouse Resource and Research Centers repository. The CTR-RELN NMR structure is available via the Protein Data Bank (PDB) platform (10.2210/pdb8g21/pdb). WES and WGS data were analyzed with the following resources: hs37d5 genome (ftp://ftp.1000genomes.ebi.ac.uk/vol1/ftp/technical/reference/phase2_reference_assembly_sequence/hs37d5.fa.gz); SW Edico Genome DRAGEN Pipeline v.01.011.231.02.05.01.40152; HW Edico Genome DRAGEN Pipeline v.01.011.231; BCFtools v.1.9 (http://samtools.github.io/bcftools/); Ensembl Variant Eeffector Predictor v.94 (https://uswest.ensembl.org/info/docs/tools/vep/index.html); gnomAD v.2.0.1 (http://gnomad.broadinstitute.org/downloads); bcbio-nextgen suite v.1.1.2 (https://github.com/bcbio/bcbio-nextgen); Exomiser v.10.1.0; Cartagenia v.5.0 (https://www.genomeweb.com/resources/new-product/cartagenia-bench-lab-50); QIAGEN HGMD Professional Database v.2018.2; OMIM latest version at the time of the data analysis; ExAC release 0.3 and gnomAD latest online version http://gnomad.broadinstitute.org/. Source data are provided with this paper.

## Source data

Source Data Fig. 2 Unprocessed western blots.
Source Data Fig. 4 Unprocessed western blots.
Source Data Extended Data Fig. 5 Unprocessed western blots.
