# Supplementary material for: Resilience to autosomal dominant Alzheimer’s disease in a Reelin-COLBOS heterozygous man
Source: Nat Med. 2023 May 15;29(5):1243–52. doi: 10.1038/s41591-023-02318-3 (PMC10202812; doi:10.1038/s41591-023-02318-3)
Supplement: Supplementary file 1 — Supplementary Results, Figs. 1–19, Tables 1–10, Methods, References and unprocessed blots for the supplementary figures. [file 41591_2023_2318_MOESM1_ESM.pdf]

nature medicine

Article

https://doi.org/10.1038/s41591-023-02318-3

Resilience to autosomal dominant Alzheimer's disease in a Reelin-COLBOS heterozygous man

In the format provided by the authors and unedited

SUPPLEMENTARY INFORMATION

SUPPLEMENTARY RESULTS

Clinical data

The male subject with *RELN-COLBOS* past medical history was significant for hypertension diagnosed at age 63 and treated with losartan (100 mg per day) and hydrochlorothiazide (25 mg per day) and benign prostate hypertrophy. His neurological exam at that age was normal except for hyperreflexia in bilateral triceps and Achilles, while his gait, balance, strength, muscle tone, and deep tendon reflexes were normal. The neuropsychological performance of the participant was adjusted for age and education using previously published normative data,[34] as reported in Table S1. His TSH, B12, folate and fasting glucose were all within normal limits. Neurological symptoms were treated with sertraline (50 mg per day) and rivastigmine (9.5 mg per day) with partial control of symptoms. The subject's mother died at a young age and, according to family reports, she had developed early-onset dementia, and his father died at age 57 from complications related to diabetes (see patient's genealogy in Supplementary Fig. 1a).

Imaging and biomarker analyses

To examine if there was a correlation between tau pathology in other brain regions and decreased cortical volume, we systematically analyze tau PET images for this individual displayed on cortical thickness as vertex-wise z-score relative to COLBOS *PSEN1* E280A non-carriers. Dice coefficient for binary maps corresponding to suprathreshold tau PET (z-score > 3.5) and subthreshold thickness (z-score < -2) was 0.49, suggesting moderate spatial overlap. We found a negative correlation between increased tau signal and cortical volume ($p = 0.0002$). These data indicate that as expected, increased regional tau burden is associated with increased cortical degeneration in the case, as observed in ADAD (Supplementary Fig. 2).

We did not identify any heterotopia or cortical developmental abnormalities previously associated with *RELN* mutations[35,36] after careful examination of the brain of the male case in the MRI. His levels of plasma neurofilament light change (NfL), a marker of neurodegeneration, were 34.7 pg/mL, which fall within the range for typical MCI carriers (41.83 ± 83.41 pg/mL[37]).

Complementary measures of RELN CTR binding to heparin as an Fc-fusion protein using biolayer interferometry

We showed that the RELN CTR H3447R peptide had 2-fold higher interaction with heparin relative to RELN WT with surface plasmon resonance (Fig. 2c). These kinetics were assessed with a synthetic CTR-RELN peptide (AA3431 to AA3460). We designed Fc-fused CTR-RELN proteins to confirm this finding in a protein produced in mammalian cells. The Fc-fusion RELN WT and H3447R mutant interactions with heparin were assessed using bio-layer interferometry (BLI; Fig. 2e). These proteins are cleaved by Furin during production in mammalian cells, resulting in short CTR-RELN variants lacking the last six amino acids. In this configuration, RELN H3447R also had about 2-fold higher affinity to heparin compared to WT (Figure 2e). We found that that the K_a is the main contributor to the difference in K_d values, which indicated that either the mutant needs less energy to interact with heparin or that this variant saturated the heparin substrate twice as fast in comparison to WT. Therefore, the CTR sequence upstream of Furin cleavage may contribute to CTR-RELN–heparin affinity in the presence of the H3447R mutation.

Mapping the heparin binding site in RELN CTR

We used high performance liquid chromatography (HPLC) to assess the contribution of specific amino acids to the interaction between heparin and the RELN CTR (Fig. 3). We designed a library of peptides including the full-length C-terminus (AA3431 to AA3460, named long) and peptides lacking the 6 amino acids normally removed by Furin (AA3431 to AA3454, named short, Fig. 3a). We replaced the arginine located at position 3446 with a histidine to determine if this amino acid contributes to heparin binding as it is located immediately before the site targeted by the H3447R variant (Fig. 3b). In addition to the H3447R variant discovered in the AD-protected individual, an acidic amino acid (H3447D) and basic amino acid (H3447K) mutation were tested to assess whether the charge of the AA at this site is critical for heparin interaction (Fig.3 c, d).

Increased peptide-heparin interaction results in delayed retention times in isotonic salt conditions (1M KCl) measured by HPLC. Generally, the long RELN peptides had delayed retention time compared to short peptides regardless of any other mutation. This suggested that the last six AAs in the RELN CTR contain a GAG binding site, which we named alpha-GAG binding site. This binding site, which overlaps with the previously described NRP1 binding site, includes two consecutive arginine residues at position 3457 and 3458 and is physiologically removed by Furin. Our data indicated early release of the RELN CTR with the R3446H mutation in both long and short peptide settings (Fig. 3). Therefore, the arginine at position 3446 is also important to heparin interaction and constitutes a second GAG binding site. This second binding site is functional in the absence of the alpha-GAG site and is not removed by Furin. We have called this the beta-GAG binding site. The H3447R mutation clearly increases affinity of the RELN CTR via modification of the beta-GAG binding site by creating two consecutive arginines (alongside 3446), similar to the configuration of the alpha-GAG site.

Surprisingly, changing the histidine for a lysine at position 3447 did not significantly increase binding to heparin. This finding suggests that an arginine at this position, as in H3447R, is critical in creating a higher affinity heparin binding site similar to the alpha-GAG site. It is possible that the planar arrangement of the guanidinium group of arginine, its relative rigidity, and its capacity to engage in cation-pi interactions and bidentate salt bridges, might play a role in optimal interactions with heparin compared to lysine or histidine. In contrast, the aspartic acid mutation at this location significantly reduced binding of heparin (Fig. 3 c, d). Spectrofluorometric analysis of all peptides showed similar absorption and emission wavelengths indicating that the differences observed by HPLC were likely attributed to changes in heparin binding and not to different spectroscopic fingerprints between variants.

H3447R RELN has increased affinity for Neuropilin 1

Recent work shows that the RELN CTR also interacts with NRP1[11]. We use BLI to further assess whether the H3447R mutation affects NRP1-RELN CTR interactions (Supplementary Table 8). Vascular endothelial growth factor (VEGF) is known to interact with NRP1 and was therefore used as a positive control [38]. As previously mentioned, our Fc-fusion proteins were made in mammalian cells thus were expected to be cleaved by Furin. Previously published data concluded that WT CTR did not bind NRP1 after removal of the last six amino acids by Furin. Our data showed the RELN CTR peptide without the alpha-GAG binding site located in the last six amino acids of RELN CTR had 100-fold less affinity for NRP1 than that of VEGF, consistent with the previously published data. However, with the H3447R mutation, the affinity was 10-fold higher in comparison to WT, suggesting that H3447R forms a new binding site or

significantly increase affinity for NRP1 via optimization of the beta-GAG site. In sum, RELN H3447R peptide has ~10-fold increased affinity to NRP1 in comparison to WT RELN peptide, much closer to the affinity range of VEGF.

Structural characterization of the RELN CTR reveals an alpha helix with a flexible C-terminus arm

We characterized the structure of CTR-RELN to better understand its function and interactions. Recently published data showed a high-resolution structure of the CR8 within the C-terminus of Reelin but were unable to define an atomic structure of the CTR-RELN, potentially due to the flexiblity of the domain [39]. We determined CTR-RELN was highly unstructured under native conditions using circular dichroism (CD, Extended Data Fig. 3b). Addition of 50% TFE (2, 2, 2-Trifluoroethanol) stabilized the unstructured CTR and enabled characterization of the domain. With 50% TFE, the peptide secondary structure is more closely an alpha-helix, with characteristic negative minima at 208 nm and 222 nm. We cannot exclude the presence of unstructured regions within the CTR-RELN even in the presence of TFE. The structure of disordered peptides may account for the "antiparallel β-sheet", or "other categories" found as a larger percentage structure in the peptide alone compared to the peptide with 50% TFE [40,41] (Extended Data Fig. 3b and Supplementary Table 7).

Using NMR, we confirmed that the C-terminal domain of RELN in the presence of TFE forms an alpha helix with a highly flexible arm towards the C-terminal region (Fig 2f, and Supplemental Videos 1-3). The vertex of the flexible region spans from the glycine residue at position 3444 to the histidine 3447 mutated in the protected case. We have named this region the flexibility vertex domain (FVD; Supplemental Videos). Consensus motifs for GAG binding include basic amino acids on the same side of the alpha helix and on contiguous turns separated by ~3.5 amino acids to allow for strong bonds [42]. Our structural data in conjunction with our functional analysis indicate that the alpha-GAG binding site is likely to depend upon R3454, R3457, and R3458 (Fig. 3a-b). As we concluded earlier from our functional studies, R3446 also mediates GAG interactions probably in conjunction with R3451 comprising the beta-GAG binding side. It is clear from the structure that the arginine introduced by the mutation at position 3447 found in the protected case is properly orientated for GAG interactions. Importantly, the new beta-GAG binding site is not cleaved by Furin, becoming a constitutively active site for RELN interaction with GAGs, NRP1 and potentially other receptors. Polar contacts between the WT CTR-RELN and heparin suggest that this interaction is likely mediated by basic amino acids in the alpha- and beta- GAG binding sites (Supplementary Table 8).

We acknowledge that the structural data obtained has limitations, as it was obtained in the presence of TFE, which was used to stabilize the native but non-persistent secondary structure of the RELN peptide. However, it should be noted that TFE may also introduce non-native alpha-helical structures in peptides. Despite these limitations, the structural work has provided valuable insights and generated hypotheses regarding interactions. We hope that it will serve as a foundation for future analyses of structure and function under more physiologically relevant conditions.

Genetic analyses

We verified the presence of the H3447R variant via DNA Sanger sequencing (Supplementary Fig. 1b). Single-cell RNA transcriptomics (scRNA-seq) of peripheral blood mononuclear cells (PBMC) showed variant allele expression of the *PSEN1* E280A mutation (Supplementary Table 4). The subject had normal lipid levels in blood, unlike the APOE3 Christchurch carrier, who, as expected[43], had hyperlipoproteinemia type III (Supplementary Table 5). Both coding and non-coding variants were analyzed with Genomiser [44] as part of Exomiser, with whole-genome CADD, (ReMM), and allele frequency data plugins.

RELN-COLBOS was not detected in 117 individuals from the Colombian kindred for which genomic information was available[45]. gnoMAD database[46] lists an allelic frequency of 0.000007963 for the *RELN* H3447R variant driven by identification in Latino populations.

We directly compared genetic data from the *APOE3* Christchurch and the *RELN-COLBOS* cases to identify any shared variants that could potentially explain the observed phenotype. We identified a total of 204 shared variants between the cases including coding and non-coding variations. None of these variants except for the expected PSEN1:ENST00000261970:c.839A>C:p.(Glu280Ala) variant were identified as top picks from the Genomizer analysis for variant prioritization relevant to Alzheimer's disease or matched variants reported by the Human Gene Mutation Database or the ClinVar databases. The number of shared variants is expected for individuals from this extended pedigree descending from a population that was established in the 16th-17th century through the admixture of Amerinds, Europeans and Africans and grew in relative isolation until the late 19th century [47,48].

We identified a total of 10 coding variants in *RELN* in a group of 83 carriers of the *PSEN1* E280A mutation for whom data was available including the *RELN-COLBOS* variant (H3447R, T3430P, G2115S, L2057R, F1944C, V1762I, L997V, S630R, S600F and Q9P). Of these, all of them were common and likely neutral except for *RELN-COLBOS*, T3430P and L2057R. The T3430P variant is extremely rare found in only one additional individual in the gnomAD database (Latino, population frequency: 0.000003981) and not previously associated to any clinical report. We found three carriers of the T3430P variant with disease onset within the expected range (average: 49 years) in our cohort of subjects with the *PSEN1* E280A. We found the L2057R in 23 subjects from the gnomAD database (population frequency: 0.00008141) mostly of European ancestry but also in some Latinos (4 samples). This variant is listed as of uncertain significance because of a single report of a link to "epilepsy, familial temporal lobe, Normal-Roberts syndrome". We found this variant in two subjects with the *PSEN1* E280A mutation with disease onset within the expected range (average: 49 years). In conclusion, we could not demonstrate any other variants in our population linking *RELN* to changes in the age at onset of ADAD.

Molecular analyses

Substitution of histidine at position 3447 with aspartic acid, a highly acidic amino acid (H3447D), reduced heparin interactions whereas substitution for lysine (H3447K), a basic amino acid, had minimal effects, further supporting a critical role for position 3447 in GAG binding. Surface plasmon resonance (SPR) measures of kinetic constants showed that the affinity of CTR-RELN H3447R is about twice that observed in the WT: H3447R>WT>H3447K>>>H3447D (Fig. 2c, & Supplementary Fig. 6a, b). RELN H3447D could not be produced in sufficient quantities for signaling analysis. This limitation is consistent with previous reports of specific mutations of the C-terminus that limit secretion of RELN [49].

We used ELISA to evaluate direct binding of recombinant RELN H3448R to canonical RELN receptors VLDLr and APOER2[50] to further examine the molecular mechanism leading to the observed gain-of-function (Supplementary Fig. 3a,b). We used experimental conditions with RELN H3448R, RELN WT, or with an equimolar mixture of WT and mutant proteins because RELN oligomerization is a critical property modulating binding to receptors [50]. In all conditions, full-length RELN H3448R and RELN WT did not differ in their ability to bind to VLDLr or APOER2. We further determined that the C-terminal region of RELN (CTR-RELN) WT or CTR-RELN H3447R recombinant peptides were unable to bind directly to VLDLr or APOEr2 (Supplementary Fig. 3c, d). This finding confirms previous reports demonstrating that the fifth and sixth reelin repeats of RELN (R5-6) are sufficient to mediate binding to the receptors [50]. We concluded that the gain-of-function of the RELN H3447R variant was not likely explained by changes in binding affinity for VLDLr or APOEr2 receptors.

Development of the *mRELN* H3448R KI mouse model for *in vivo* studies
The *RELN* gene (NCBI Reference Sequence: NM_011261.2) is located on mouse chromosome 5. 65 exons have been identified, with the ATG start codon in exon 1 and TGA stop codon in exon 65. We generated the *RELN* H3448R-Tg knock in (KI) mouse model at TACONIC/Cyagen as fee-for-service by introducing the H3448R (CAC to CGT) mutation into exon 64 in 3' homology arm of the *RELN* gene. In the targeting vector, the Neo cassette was flanked by SDA (self-deletion anchor) sites. Diphtheria toxin (DTA) was used for negative selection. The *RELN* targeting construct was linearized by restriction digestion with AscI followed by phenol/chloroform extraction and ethanol precipitation (refer to supplementary table 9 for the list of primers". The linearized vector was transfected into C57BL/6N Embryonic Stem (ES) cells according to cyagen's standard electroporation procedures. The transfected ES cells were subject to G418 selection (200 µg/mL) 24 hours post electroporation. 538 G418 resistant clones were picked and amplified in 96-well plates (Supplementary Fig. 7). Two copies of 96-well plates were made, one copy was frozen down and stored at -80°C and the other copy of the 96-well plates was used for DNA isolation and subsequence PCR screening for homologous recombination. The PCR screening identified six potential targeted clones, from among which six were expanded and further characterized by Southern blot analysis (Supplementary Fig. 7). Five of the six expanded clones were confirmed to be correctly targeted. Proper targeting was confirmed by Southern blot and PCR. KI mice were generated upon injecting targeted ES cells into the blastocysts that were introduced into the foster mothers for generating the mouse crossings. Targeted ES cell clones 6C10 and 5C10 were injected into C57BL/6 albino embryos, which were then re-implanted into CD-1 pseudo-pregnant females. Founder animals were identified by their coat color, their germline transmission was confirmed by breeding with C57BL/6 females and subsequent genotyping of the offspring. Two female heterozygous targeted mice were generated from clone 5C10, two male and two female heterozygous targeted mice were generated from clone 6C10. Mice from 6C10 were used for the analysis because we had more founders.

Neuropathology studies
The brain of the *RELN-COLBOS* index case, male, presented frontal lobe predominant atrophy. As expected, Aβ pathology (signal intensity/evaluated area) was higher in cortical structures whereas cerebellum and other subcortical structures were less affected. Extensive Aβ signal in cortical and subcortical structures was composed of diffuse plaques, while hippocampus, uncus

and amygdala presented mainly core and mature plaques (Fig. 5a, Extended Data Fig 6). ptau pathology (signal intensity/evaluated area) was also higher in cortical structures with medial and inferior temporal cortices, which presented with the highest signal, as previously described for *PSEN1* E280A cases[51] (Extended Data Fig 7). The morphology of ptau pathology was characterized by dystrophic neurites and diffuse signal with fewer neurofibrillary tangles in all evaluated structures (Extended Data Fig 7). Extended microgliosis and astrocytosis were observed in all evaluated areas with more intense microglia (Iba1 positive cells) relative signal in medulla oblongata, and intense astrocyte (glial fibrillary acidic protein, GFAP, positive cells) relative signal in medial temporal cortex and medulla oblongata (Supplementary Fig. 14, 15 and Extended Data Fig 8), consistent with previous reports in Alzheimer's Disease (AD) [52,53]. Observed high levels of Aβ pathology in all studied structures support further a lack of relevant effects of the non-coding APP variant found in this patient. Also, even though other RELN mutations (loss of function) have been associated to abnormal neuronal migration[54], we were not able to identify pathological changes in the cortex beyond those related to the neurodegenerative process (Supplementary Fig. 16).

Hippocampal structures showed almost exclusively mature and core plaques, while entorhinal cortex presented diffuse plaques and parahippocampal cortex presented with core plaques with diffuse pathology in the fusiform gyrus and inferior temporal gyrus (Fig. 5a & Supplementary Fig. 17). Hippocampal structures presented mixed ptau pathology. Dentate gyrus, *Cornu Ammonis* (CA) 4, CA3, CA2 and rostral CA1 presented predominantly with neurofibrillary tangles, while pyramidal CA1 and subiculum presented mainly with diffuse ptau pathology. All except subiculum presented mainly diffuse ptau pathology (Fig. 5a, Supplementary Fig. 18 & Extended Data Fig. 9).

We evaluated C-terminal RELN and ApoE signal in the hippocampal and parahippocampal subregions. Total signal was not different between the protected cases and familial AD (FAD) and sporadic AD controls, though we noted some differences in signal localization of ApoE and RELN. Intraneuronal distribution of ApoE was prominent in AD cases, as previously reported [55], whereas it was largely absent from this location in the *APOECh* and *RELN-COLBOS* cases (Fig. 5d). RELN signal distribution was not different in the RELN-COLBOS compared to other cases (Supplementary Fig. 18 & 19) except in white matter where it showed intense intracellular signal (Fig. 5d). In fact, out of the *PSEN1* E280A cases, the RELN-COLBOS case showed the strongest evidence of preserved white matter among studied AD cases (Extended Figure 9, 10), that together to a higher number of RELN signal in white matter, indicated a possible association between RELN oligodendrocyte signal and white matter preservation (Extended Figure 10 c, e).

Supplementary Table 1. Test scores and percentiles for age & education. The subject's age and education-adjusted neuropsychological test scores reveal a pattern of significant global and progressive cognitive decline during a 5-year assessment period.

	**Initial visit (67 yo)**	**3-year visit (70 yo)**	**4-year visit (72 yo)**	**5-year visit (73 yo)**	
**Cognitive Tests**	**Raw Scores (Percentiles*)**				**Mean (SD)***
**MMSE/30**	29 (61st)	23 (1st)	16 (<1st)	7 (<1st)	28.33 (2.24)
**Naming/15**	14 (86th)	15 (95th)	13 (70th)	11 (30th)	12 (1.89)
**CERAD Word List Learning/30**	7 (1st)	5 (<1st)	5 (<1st)	1 (<1st)	15.58 (3.62)
**CERAD Word List Delayed Recall/10**	3 (13th)	2 (5th)	2 (5th)	0 (1st)	5.46 (2.13)
**CERAD Word List Recognition/10**	3 (<1st)	6 (<1st)	9 (39th)	2 (<1st)	9.37 (1.3)
**CERAD Constructional Praxis Copy/11**	10 (66th)	4 (<1st)	5 (1st)	4 (<1st)	9.29 (1.78)
**CERAD Constructional Praxis Recall/11**	9 (73rd)	5 (27th)	0 (2nd)	0 (2nd)	6.92 (3.36)
**Semantic Fluency (Animals)**	8 (2nd)	6 (<1st)	4 (<1st)	3 (<1st)	16.79 (4.17)
**Phonemic Fluency (FAS)**	26 (68th)	7 (13th)	14 (30th)		20.39 (12)
**Raven's Matrices Test/12**	9 (73rd)	7 (37th)	5 (12th)		7.71 (2.23)
**GDS/15**	5	4	2		
**EDG/7**	1	3	5	6	

Note. MMSE, Mini Mental Status Examination; CERAD, Consortium to Establish a Registry for Alzheimer's Disease Test Battery; GDS, Geriatric Depression Scale; EDG, Global Deterioration Scale. Percentiles were calculated using age and education-based norms from a Colombian population at age 65 and above and expressed with mean and standard deviation (SD); see Torres, Vila-Castelar et al. 2019 [34] for full description of the neuropsychological measures and normative data. The following guidelines can be used for a qualitative interpretation of his performance: 90-75 percentiles = above average; 74-25 percentiles = average; 24-9 percentiles = below average; 8-2 percentiles = borderline; 1 = extremely low*.

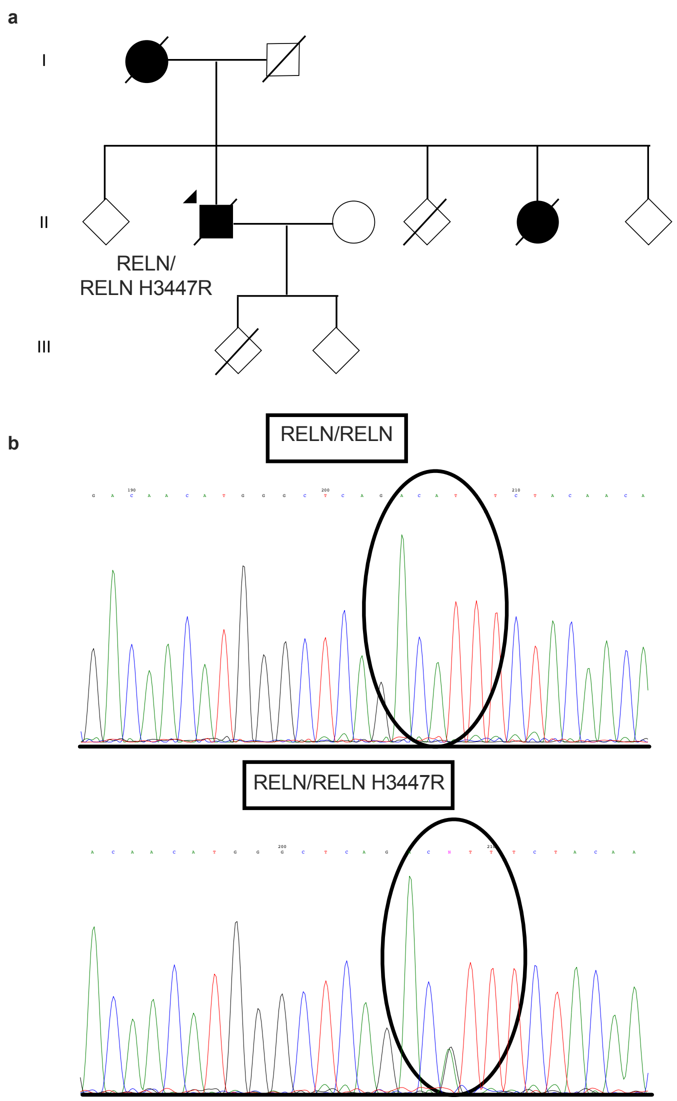

Supplementary Figure 1. Subject's genealogy and Sanger validation of the C-terminus *RELN* H3447R variant. a, Subject's genealogy. Circles representing females, squares representing males, diamonds representing individuals whose gender has been masked for privacy. Arrowhead depicts proband case. Deceased individuals are marked with a crossed bar. Black shapes indicate affected individuals and white shapes indicate unaffected individuals. **b**, Representative DNA Sanger sequencing of amplicon in *RELN* gene from a carrier that does not carry the mutation (*RELN/RELN*, top panel), as compared to the individual carrying the variant (*RELN/RELN* H3447R, bottom panel). Region of the H3447R mutation is highlighted by the black circle.

Supplementary Table 2. Demographic information of Colombia-Boston biomarker research study cohort.

	Age range (SD*)	Number of participants	Education range (SD)	MMSE (SD)
**PSEN1 E280A Impaired Carriers**	44.7 years (2.4)	11	8.5 years (4.8)	22.8/30 (3.8)
**Cognitively unimpaired Carriers**	29-46 years (34.3, 4.3)	18	9.5 years (4.0)	28.2/30 (1.0)

*SD = Standard deviation

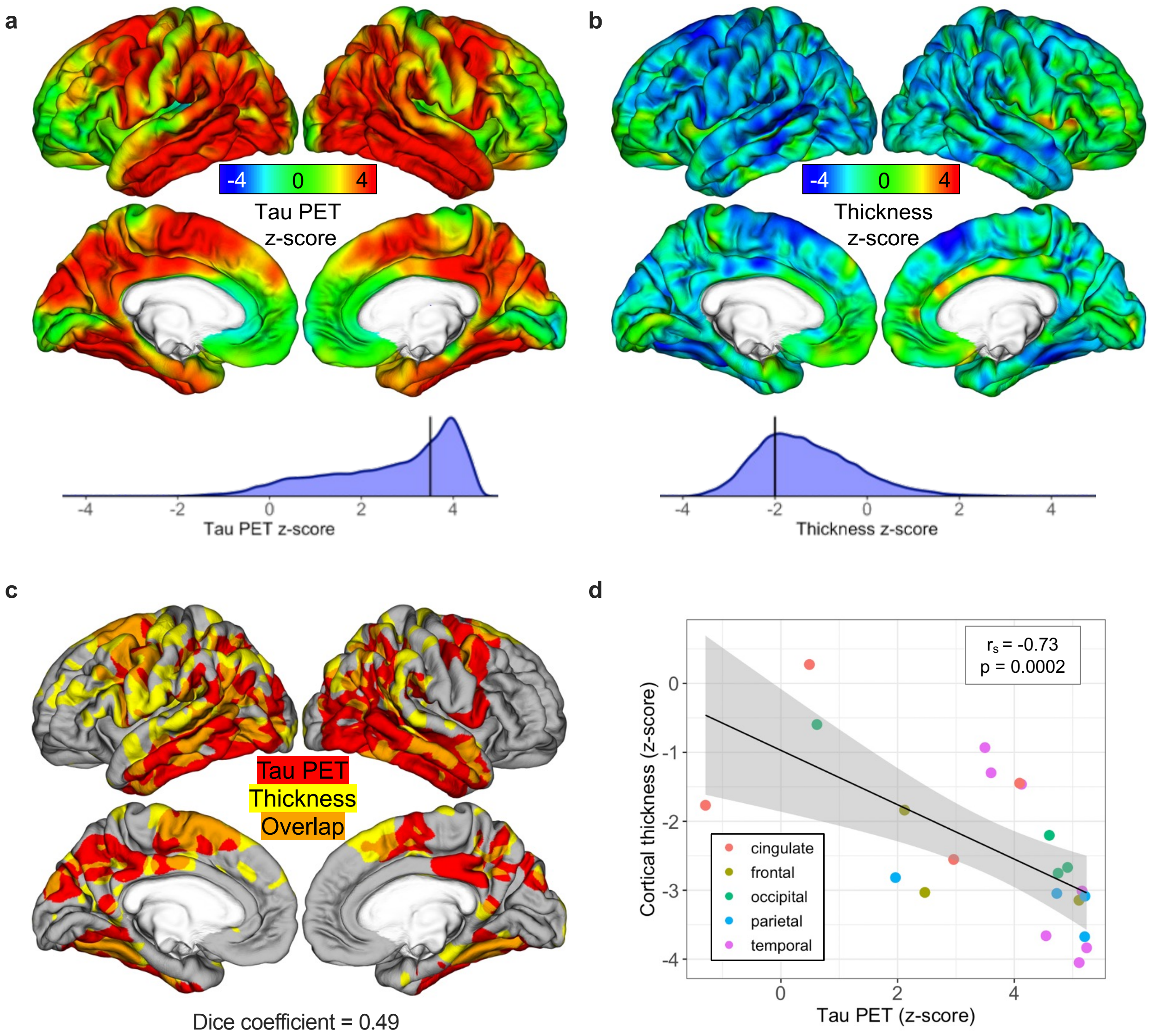

Supplementary Figure 2. Tau PET and thickness measures in *RELN-COLBOS* (H3447R) carrier. a, Representative tau PET images for this individual displayed on a cortical rendering, as vertex-wise z-score relative to COLBOS *PSEN1* E280A non-carriers; histogram below shows the distribution of z-scores. **b**, Cortical thickness surfaces are shown as z-score relative to COLBOS non-carriers; with histogram below as in Panel **a**. **c**, Surface map showing the overlap (orange) of suprathreshold tau PET (red) and subthreshold thickness (yellow); z-score thresholds indicated by vertical lines in (**a**) and (**b**) (tau: z-score > 3.5; thickness: z-score < -2). Dice coefficient for these two binary maps was 0.49, suggesting moderate spatial overlap. **d**, Correlation analysis between tau PET and thickness in regions of interest (dots), color-encoded by lobe according to inset legend. Spearman correlation (r_s) and p-value given in inset text. In grey is represented the 95% confidence interval for predictions from a linear model of cortical thickness as a function of tau PET z-score.

Supplementary Table 3. Variant allelic expression of the *PSEN1* variant 17:G.73664808a>C (E280A) in peripheral blood mononuclear cells

Case	Genotype	Reference allele (reads)	Variant allele	Total allele
***PSEN1* E280A and *RELN* H3447R carrier**	0/1	**17**	**21**	38
PSEN1* E280A carrier	0/1	**11**	**13**	24
**Non carrier***	0/0	**30**	**0**	30

* Data previously published in Arboleda-Velasquez et al., 2019 are reported here as a reference [2].

Supplementary Table 4. Single-cell RNA sequencing.

Characteristic	***RELN*** **H3447R carrier**
**Estimated Number of Cells**	3716
**Mean Reads per Cell**	43470
**Median Genes per Cell**	1715
**Description:**	
**Transcriptome**	hg19
**Chemistry**	Single Cell 3' v3
**Cell Ranger Version**	3.0.2
**Sequencing:**	
**Number of Reads**	161538005
**Valid Barcodes**	0.978
**Sequencing Saturation**	0.651
**Q30 Bases in Barcode**	0.973
**Q30 Bases in RNA Read**	0.901
**Q30 Bases in Sample Index**	0.97
**Q30 Bases in UMI**	0.97
**Mapping:**	
**Reads Mapped to Genome**	0.959
**Reads Mapped Confidently to Genome**	0.916
**Reads Mapped Confidently to Intergenic Regions**	0.063
**Reads Mapped Confidently to Intronic Regions**	0.297
**Reads Mapped Confidently to Exonic Regions**	0.556
**Reads Mapped Confidently to Transcriptome**	0.527
**Reads Mapped Antisense to Gene**	0.011
**Cells:**	
**Estimated Number of Cells**	3716
**Fraction Reads in Cells**	0.94
**Mean Reads per Cell**	43470
**Median Genes per Cell**	1715
**Total Genes Detected**	19761
**Median UMI Counts per Cell**	5329

Supplementary Table 5. Lipid panel of the *RELN* H3447R mutation carrier.

Test: **Blood lipid panel**	**Subject**	**Normal range***
**Triglycerides (mg/dL)**	84	<150
**Total Cholesterol (mg/dL)**	125	<200
**VLDL-C (mg/dL)**	(-)	<30
**LDL-C (mg/dL)**	53.7	≤130
**Direct LDL-C (mg/dL)**	(-)	≤130
**HDL-C (mg/dL)**	54.5	≥40
**Cholesterol/HDL ratio**	2.29	<5

Note: Dyslipidemia workup: VLDL-C, very low-density lipoprotein cholesterol; LDL-C, Low-density lipoprotein cholesterol; HDL-C, High-density lipoprotein cholesterol; (-), Data not available.

*Normal range according to Merck Manual and Laboratory values.

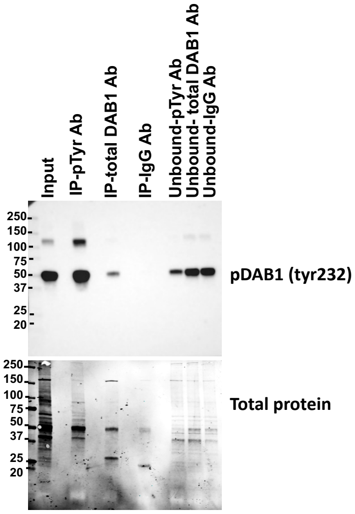

Supplementary Figure 3. Validation of anti pDab1 (tyr232) antibody. Representative western blotting of anti-pDab1 positive bands (top blot) and total protein staining (bottom blot) for the immunoprecipitation of mouse frontal cortex tissue (input) using either anti-phosphorylated tyrosine (IP-pTyr Ab), anti-total Dab1 (IP-total Dab1 Ab) and anti-mouse IgG1 (IP-IgG Ab) conjugated beads, along with unbound fractions for all antibodies tested. Data confirms the presence of pDab1 in both IP-pTyr Ab and IP-total Dab1 Ab and not in the IP-IgG Ab pull down fraction, as compared to input and unbound fractions.

a

Sequence

LIGIDEVSAAR

Predicted Fragmentation Pattern

Seq	#	b: Δ Error	b	y	y: Δ Error	+1
L	1	---	114.091	---	---	11
I	2	-700.057	227.175	1030.553	---	10
G	3	-633.628	284.197	917.469	-69.721	9
I	4	-381.120	397.281	860.447	-56.062	8
D	5	-184.128	512.308	747.363	-64.602	7
E	6	-401.390	641.350	632.336	-140.901	6
V	7	36.603	740.419	503.294	-99.417	5
S	8	81.228	827.451	404.225	333.158	4
A	9	294.341	898.488	317.193	-227.796	3
A	10	---	969.525	246.156	-313.757	2
R	11	---	---	175.119	---	1

b= charge retained in the N-term region
y=charge retained in the C-term region

b

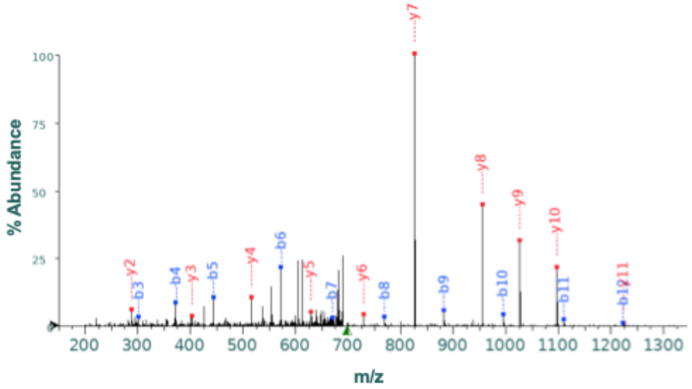

c

Sequence

TAQAAEPVILDLR

Predicted Fragmentation Pattern

Seq	#	b: Δ Error	b	y	y: Δ Error	+1
T	1	---	102.055	---	---	13
A	2	---	173.092	1295.732	---	12
Q	3	-925.386	301.151	1224.695	-243.831	11
A	4	-399.353	372.188	1096.636	-88.148	10
A	5	-485.422	443.225	1025.599	-75.678	9
E	6	-229.059	572.267	954.562	-48.756	8
P	7	99.783	669.320	825.519	-62.675	7
V	8	-83.931	768.389	728.467	274.059	6
I	9	-143.411	881.473	629.398	-301.414	5
L	10	126.702	994.557	516.314	-298.691	4
D	11	-80.294	1109.584	403.230	-380.626	3
L	12	181.730	1222.668	288.203	-922.132	2
R	13	---	---	175.119	---	1

d

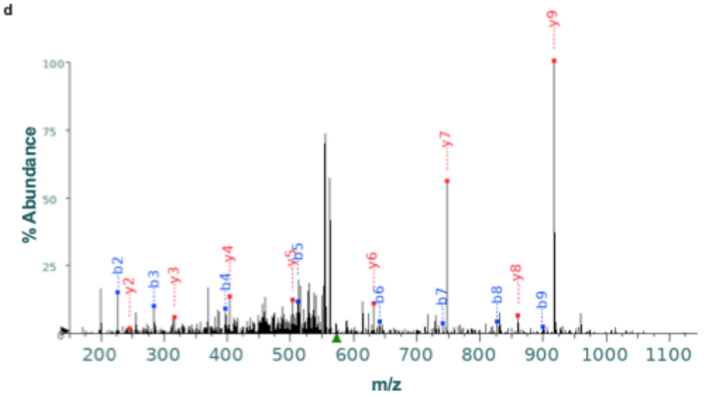

Supplementary Figure 4. Mass spectrometry (MS) validation of anti DAB1 antibody.
a, **c**, Sequences and predicted fragmentation patterns of detected fragments of Dab1 protein detected in protein fractions of mouse brain homogenate immunoprecipitated by the anti-Dab1 conjugated beads presented in Supplementary Figure 3. **b**, **d**, Representative MS plots expressed as percentage of abundance over ratio of mass to charge (m/z).

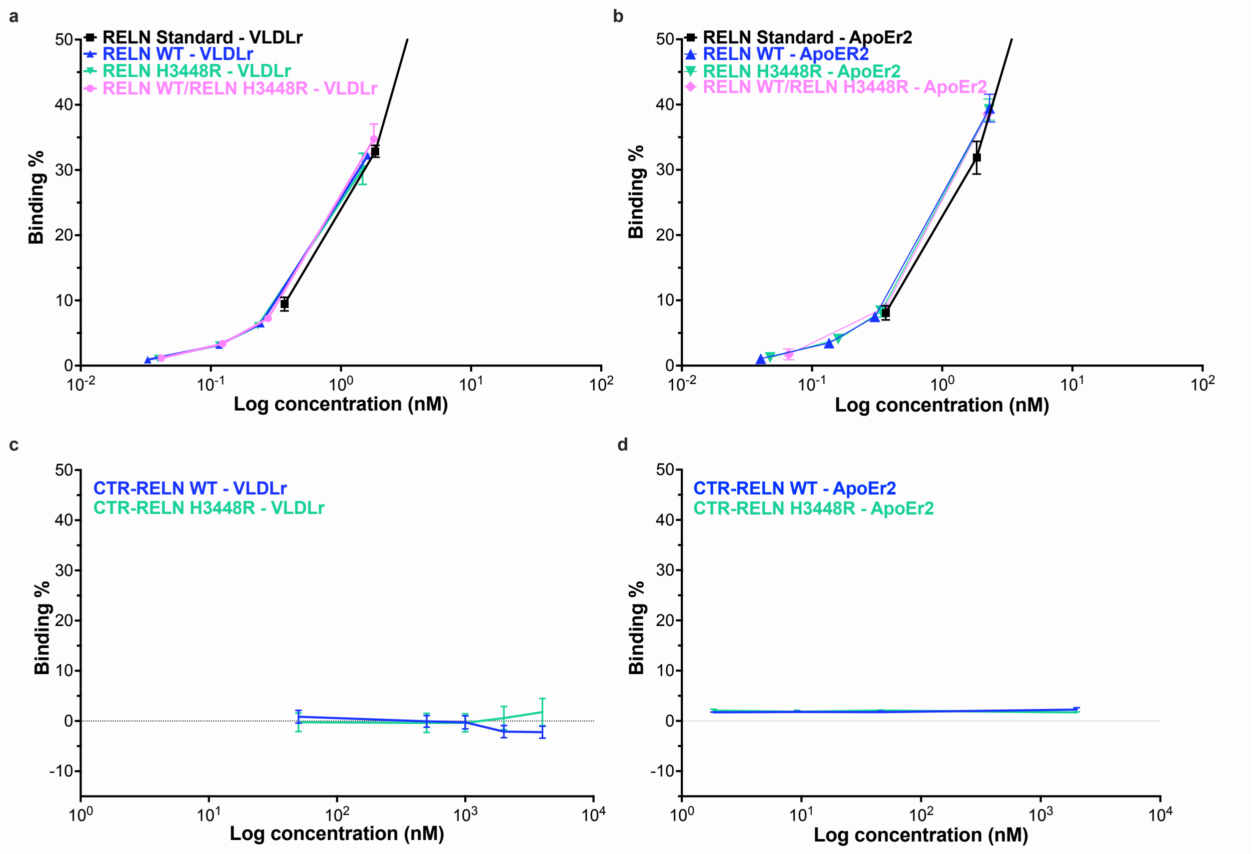

Supplementary Figure 5. The RELN H3448R variant does not affect binding to VLDLr and ApoEr2 receptors. **a**, **b,** Representative plots of ELISA binding profiles between VLDLr (**a**) or ApoEr2 (**b**) and conditioned media containing full-length RELN WT (blue curves) and RELN H3448R (green curves), as compared to the recombinant intermediate domain of RELN used as standard (RELN Std Standard, black curves). Binding to the receptor of a 50:50 mixture containing full-length WT and RELN H3448R was also tested (RELN/RELN H3448, orange curves). Overall, data suggests that the H3448R mutation does not affect the binding of RELN to APOEr2 or VLDLr. Data expressed as averaged binding percentage over logarithmic concentration expressed in nM and each curve is representative of n = 3 independent experiments. Error bars expressed as s. e. m. (**c**, **d**) Representative ELISA of CTR-RELN (WT in blue and H3447R in green) binding to either VLDLr (**c**) or ApoEr2 (**d**) at increasing nanomolar concentrations (nM). Data expressed as averaged binding normalized to the RELN Std ± s. e. m. (n = 2 independent biological experiments of n = 3 technical replicates).

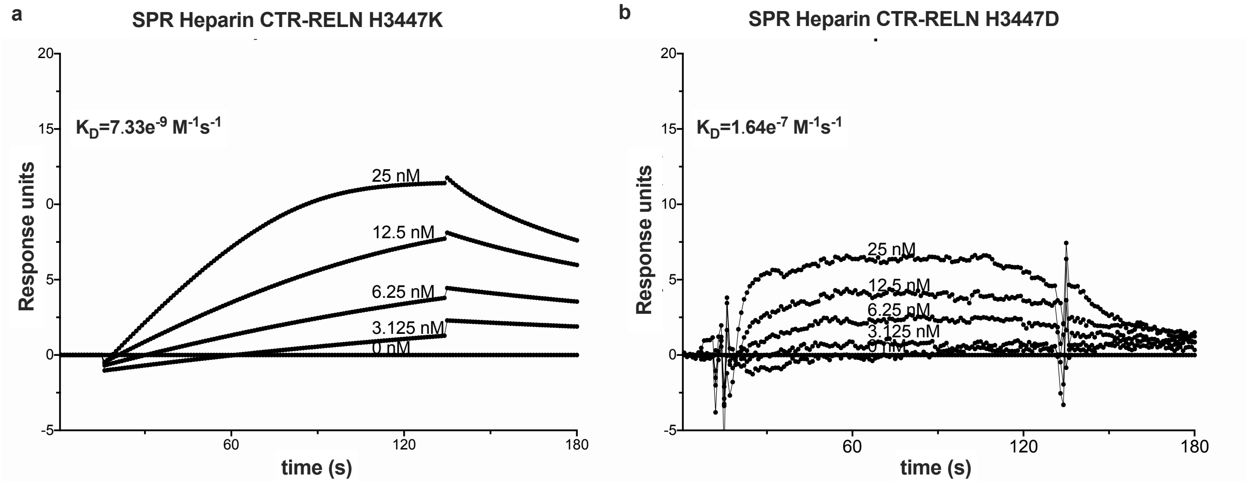

Supplementary Figure 6. Mutations at position 3447 in the CTR domain of RELN impact heparin binding. a, **b**, Representative sensorgrams of the binding analysis between chip sensors coated with heparin and increasing concentrations of CTR-RELN variants ranging from 0 to 25 nM. Sensorgrams for CTR-RELN H3447K (**a**) and CTR-RELN H3447D (**b**). Data expressed as response units over time in seconds. Equilibrium dissociation constants (k_D) for each SPR analysis are shown inside the graph and supports the difference binding between heparin and reelin variants in the order H3447R (Figure 2**c**, K_D = 3.75e-9 M-1s-1) > WT (Figure 2**c**, K_D = 6.53e-9 M-1s-1) > H3447K (**a**, K_D = 7.33e-9 M-1s-1) >>> H3447D (**b**, K_D = 1.64e-7 M-1s-1) when compared to data presented in Figure 2.

Supplementary Table 6. ITC binding parameters.

**Peptide**	**K_a**	**ΔG**
CTR-RELN WT Short	2.95E6 ± 6.49E5M^{-1} (339.0 nM)	5.008 kCal
CTR-RELN H3447R Short	3.65E6 ± 2.20E6M^{-1} (274.4 nM)	-8.952 kCal

Supplementary Table 7. Secondary structure analysis of circular dichroism spectroscopy data of CTR-RELN WT alone or with 50% TFE

Secondary structure	Peptide only	50% 2,2,2 – Trifluoroethanol (TFE)
**Helix (regular)**	0.0%	22.0%
**Helix (structural)**	0.6%	11.9%
**Antiparallel β-sheet**	33.4%	14.6%
**Parallel β-sheet**	0.0%	0.0%
**β-turn**	15.5%	10.4%
**Other**	50.5%	41.0%
**RMSD**	0.1573	0.1008

Supplementary Table 8. Analysis of binding parameter of CTR-RELN-NRP-1 interactions

Analyte	K_D	k_a	k_d
Fc	/	/	/
Fc - WT	6.10E-07 M	9.40E3 $M^{-1} \cdot s^{-1}$	5.73E-3 s^{-1}
Fc - H3447R	7.93E-08 M	1.29E4 $M^{-1} \cdot s^{-1}$	1.02E-3 s^{-1}
VEGF	5.53E-09 M	2.72E5 $M^{-1} \cdot s^{-1}$	1.50E-3 s^{-1}

Supplementary Videos:

Supplementary Video 1. 20 lowest energy structures produced by NMR shown sequentially. Each of the 20 structures were shown for 5 frames each at a rate of 75 frames per second (FPS).

Supplementary Video 2. 20 lowest energy structures produced by NMR shown in rotation. The 20 lowest energy structures shown in X-axis and Y-axis rotation at 40 FPS.

Supplementary Video 3. The 20 lowest energy structures produced by NMR shown layered on top of previously shown structures. Each structure was added to the previous structures for 5 seconds at a rate of 15 FPS.

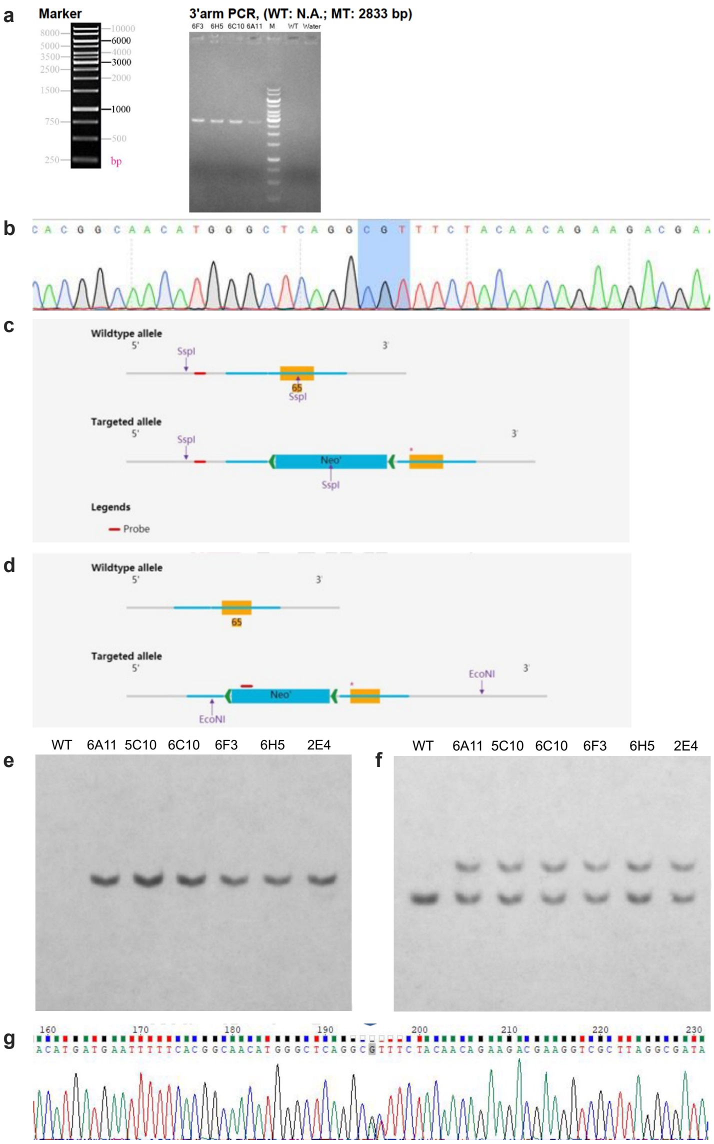

Supplementary Figure 7. Clone screening and sequencing for generating the H3448R *RELN* knock-in model. a, Representative marker ladder (left) and southern blot data (right) for the 538 G418 resistant clones screened to identify homologous recombination using PCR. Primers spanned the 3’arm from the end of the Neo cassette through outside the homology arm. As expected, a band of 2,833 bp was found in positive clones. **b**, Sanger sequencing of the 2,833 bp PCR fragments used to verify the presence of the desired variant. The WT codon for histidine in mouse sequence is different than in human. A two base pair change was made to introduce the arginine in position H3448, homologous to human H3447R: CAC to CGT codon change is highlighted in blue. As expected, only signal from the targeted allele is observed. **c**, Pictographic representation of the digestion of the wild type allele using Sspl enzyme, followed by hybridization using a 5’arm probe. Predicted bands include ~3.71 kb-WT and 4.6 kb-targeted with Sspl digestion. **d**, Schematic representation of the genomic DNA digestion suing EcoNI enzyme, followed by hybridization using a Neo probe. The probe is expected to detect a band of 9.69 kb with EcoNI digestion. **e**, representative southern blot to detect positive clones with Neo 3’arm probe after EcoNI digestion. We considered all clones correct except 2E4, due to an extra

lower molecular weight faint band. **f**, Positive clones detected with 5'arm probe upon Sspl digestion. From left to right: 6A11, 5C10, 6C10, 6F3, 6H5, 2E4. **g**, the presence of the H3448R variant was confirmed in genomic DNA from mice used for the analyses using Sanger sequencing. Double peaks indicate heterozygosity at 3448 codon: CAC to CGT, which is the orthologue of H3447 in human *RELN*.

Supplementary Table 9. List of primers used for screening and PCR sequencing.

**3'arm PCR for ES screening:**
Primers for 3'arm PCR:<br>F1: 5’-GGCTGGTAAGGGATATTTGCCTG-3’<br>R1: 5’-CAGAGTCTTCCTGTTTGGCTTTCT-3’
**Primers for sequencing PCR:**<br>F1: 5’-TTATTCTGTCCCTAGGCGGTG-3’<br>R1: 5’-CAGAGTCTTCCTGTTTGGCTTTCT-3’<br>Expected PCR Product:<br>Wildtype: N.A.<br>Product Size:2833 bp
**Primer for Sequencing:**<br>F3: 5’-TTATTCTGTCCCTAGGCGGTG-3’
**Primers for 5’arm Probe for Southern:**<br>5'arm-Probe-F: 5'-GAGAAGCAAAGGTTTCACTCAGCAGGA-3'<br>5'arm-Probe-R: 5'-GGGCCTCATTCACACACACACAATAG-3'
**Primers for sequencing PCR for mouse genotyping:**<br>F1: 5’-TTATTCTGTCCCTAGGCGGTG-3’<br>R1: 5’-AAGATCTCGAAGGGCTTTCTGTT-3’<br>Expected PCR Product:<br>Wildtype: 400 bp<br>Product Size: 400 bp<br>Primers for Sequencing:<br>F1: 5’-TTATTCTGTCCCTAGGCGGTG-3’

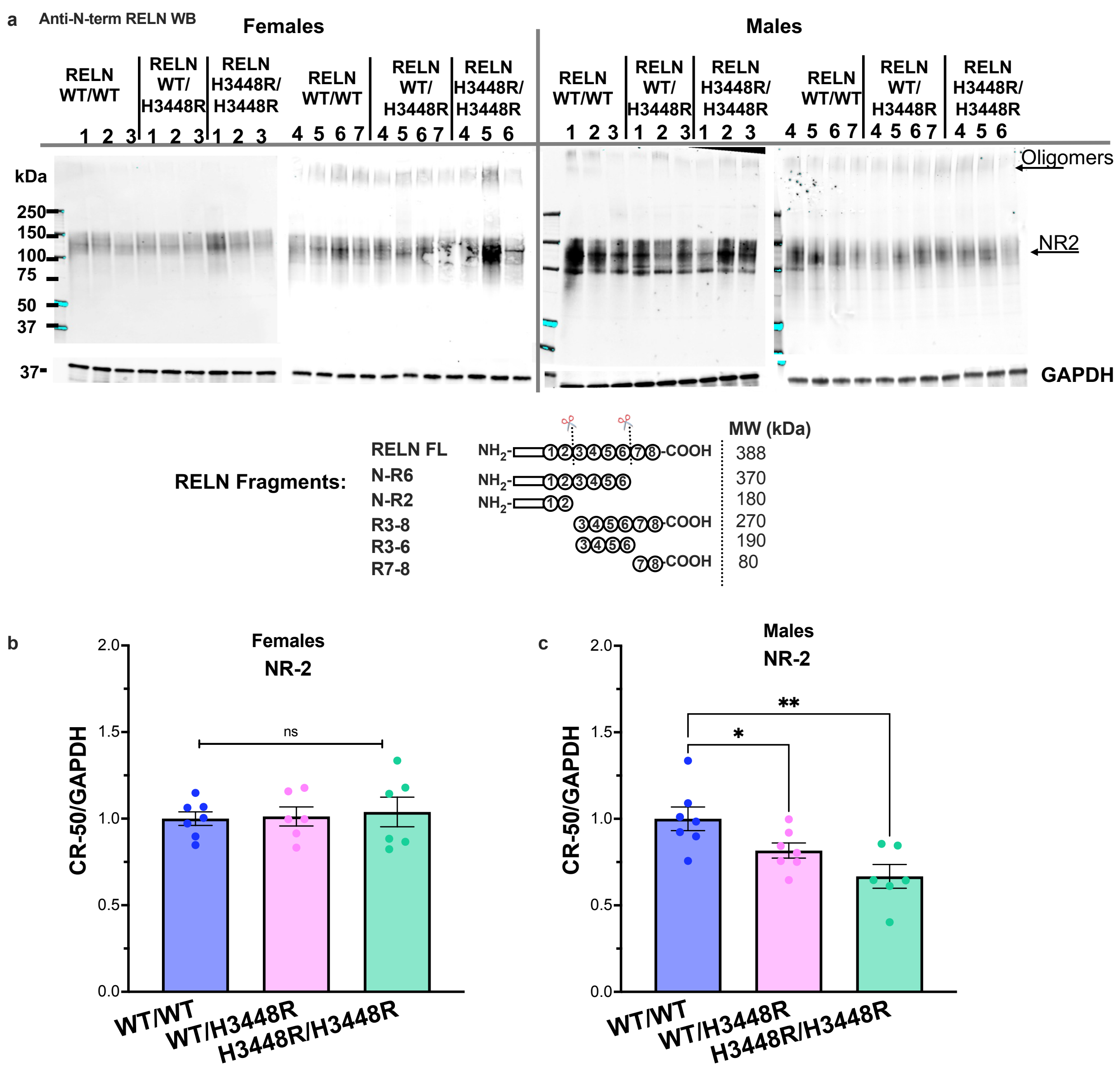

Supplementary Figure 8. The NR-2 RELN fragment is reduced in the male homozygous and heterozygous *mRELN* H3448R Tg mice. a, Representative western blots under not reducing-not denaturing conditions of anti N-terminus RELN positive bands in cerebellum homogenates isolated from females (left blots) and males (right blots) at ≈ 6-12 m. o. **b, c**, Data shows that NR-2 levels are significantly reduced in both heterozygous (*RELN* WT/H3448R, n = 7 mice) and homozygous (*RELN* H3448R/H3448R, n = 6 mice) male mice (**c**, One-way ANOVA, followed by Fisher's LSD test. p = 0.0431 WT vs HET, SE of diff. = 0.083, n1 = n2 = 7, t = 2.185, DF = 17; p = 0.0014 WT, SE of diff. = 0.08733, n1 = 7, n2 = 6, t = 3.809, DF = 17; n = 7 mice, vs. *RELN* H3448R/H3448R, n = 6 mice) and not in the female mice (**b**), as compared to wild type sex-matched littermates. All data is presented as average ± s. e. m.

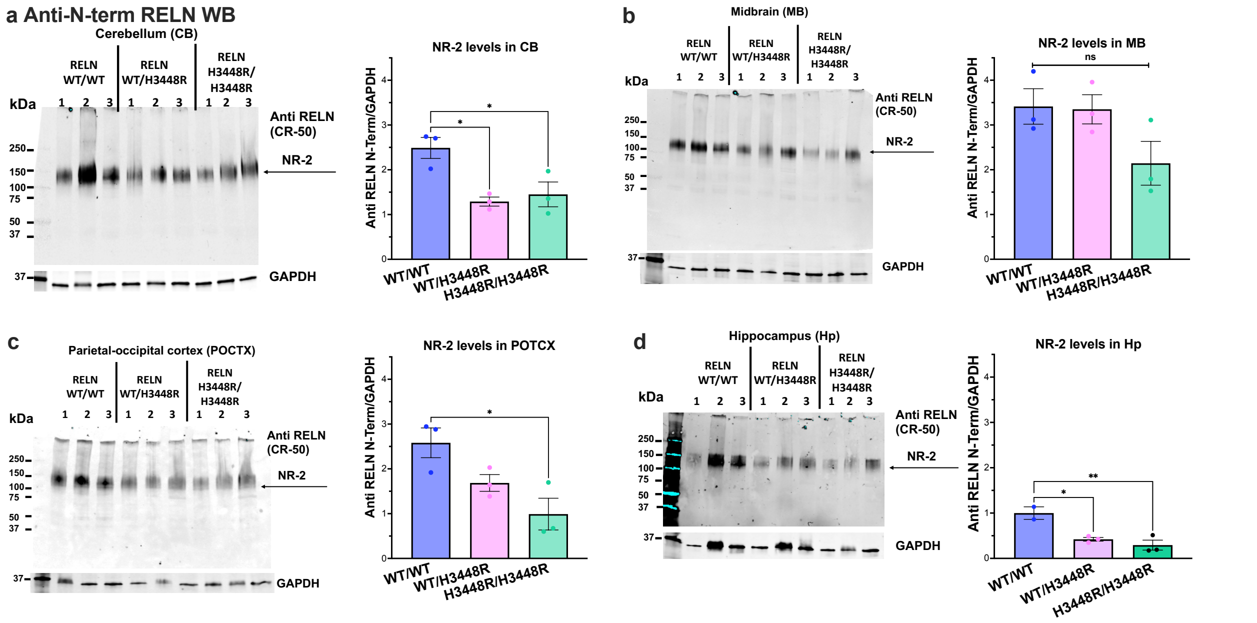

Supplementary Figure 9. RELN NR-2 fragment levels in different brain regions in the *RELN*-H3448R Tg mouse model. **a-d,** Western blotting and quantification of normalized to GAPDH levels of NR-2 fragments detected with the anti-RELN antibody CR-50 in the total homogenates of n = 3 brain sections obtained from cerebellum (**a**), midbrain (**b**), parietal-occipital cortex (**c**), and hippocampus (**d**) from *RELN* WT/WT, *RELN* WT/H3448R and *RELN* H3448R/H3448R male mice at 16 weeks of age. Data is showing that there is a significant reduction of the levels of the NR-2 fragments in the presence of the H3448R mutation in all regions investigated except for the midbrain regions. Panel **a** *p = 0.0141 WT/WT vs. WT/H3448R, n1=n2 = 3, q = 3.904, DF = 6; *p = 0.00262 WT/WT vs. H3448R/H3448R, n1=n2 = 3, q = 3.379, DF = 6; panel **c** *p = 0.0169, n1=n2 = 3, q = 3.748, DF = 6; panel **d** *p = 0.0162, n1=n2 = 3, q = 4.074, DF = 5 **p = 0.0071, n1=n2 = 3, q = 4.976, DF = 5. One-way ANOVA followed by Dunnett's multiple comparisons test has been used for statistical comparisons. All data is presented as average of n = 3 mice ± s. e. m.

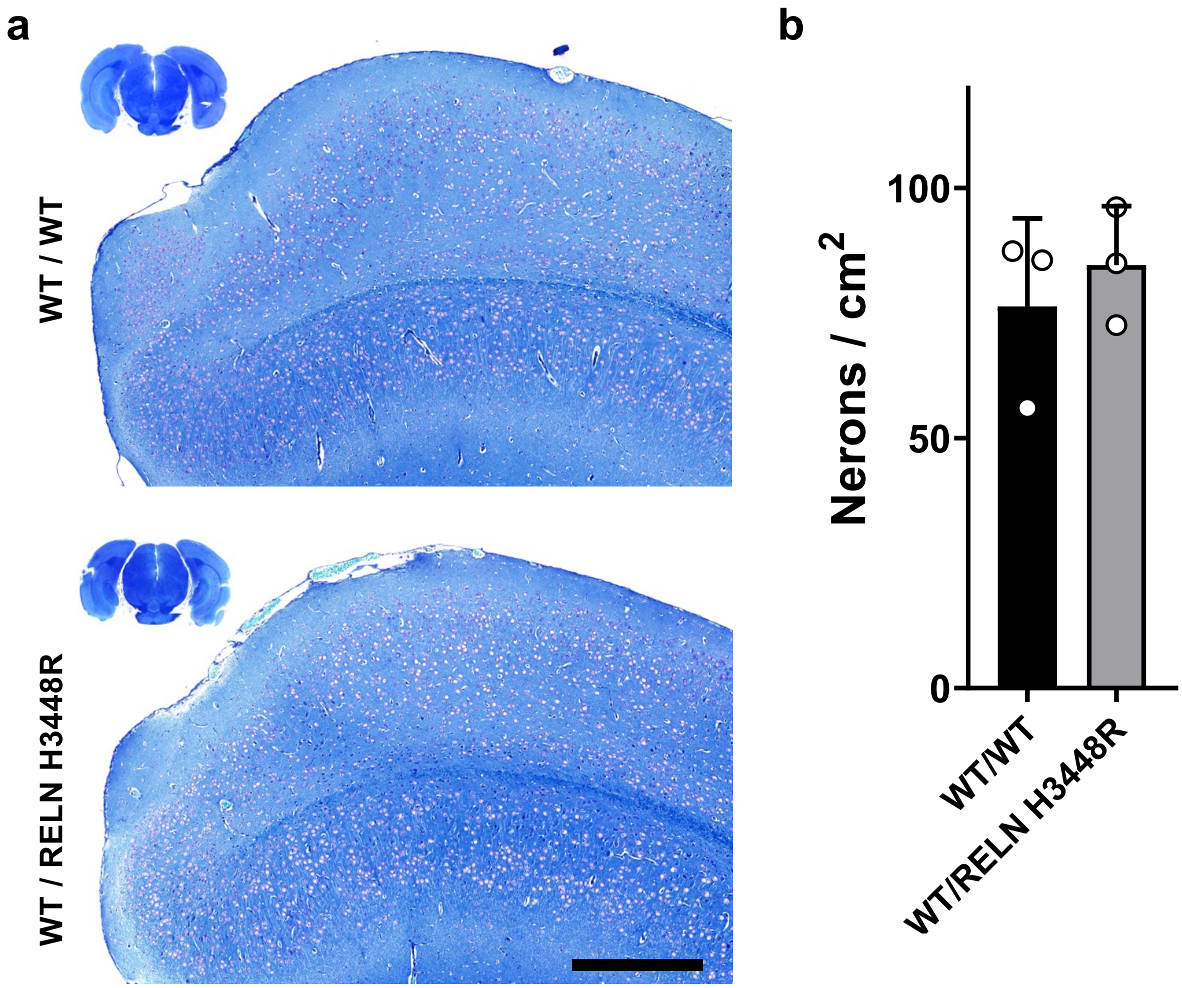

Supplementary Figure 10. Klüver-Barrera evaluation of entorhinal cortex neuronal density in *RELN* H3448R mice. **a**, Representative staining of coronal section (smaller image) and hippocampal regions of *RELN* WT/WT and *RELN* H3448R (homozygous for *RELN* variant, labeled as noted to indicate absence of tau transgene) male mice at 18 months of age, n = 3. There were no obvious morphological differences between the groups. Bar = 1 mm. **b**, Bar graph for neuronal density in WT / WT (n = 3 mice) and WT / *RELN* H3448R (n = 3) hippocampal regions represented in (**a**) showing that there is no genotype effect on neuron number (p = 0.538, two-sided Student's T. Test). Error bars represent standard deviation from mean of n = 2 technical replicates from n = 3 mice experiments

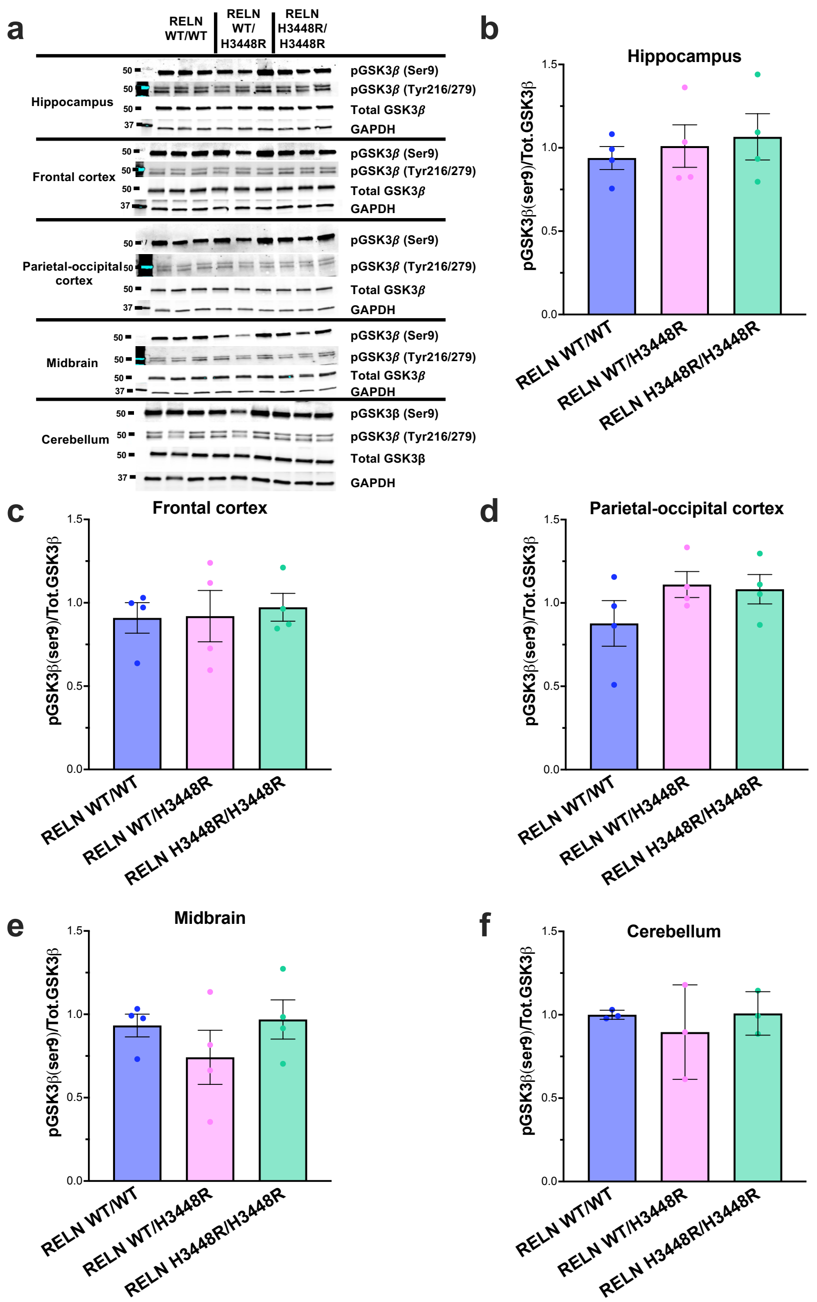

Supplementary Figure 11. pGSK3β (Ser9 and Tyr216/279) levels in different brain regions in the *RELN*-H3448R Tg mouse model. **a**, Representative blots using anti-pGSK3β (Ser9), anti-GSK3β (Tyr216/279), anti-total GSK3β or anti-GAPDH antibodies to detect protein changes in homogenized tissue from (top to bottom) hippocampus, frontal cortex, parietal-occipital cortex, midbrain, and cerebellum of *RELN* WT/WT, *RELN* WT/H3448R and *RELN* H3448R/H3448R mice (n = 3 mice). (**b-f)** Quantifications of pGSK3β(Ser9 and Tyr216/279) normalized to total GSK3β and GAPDH levels of n = 3 homogenized sections obtained from hippocampus (**b**), frontal cortex (**c**), parietal-occipital cortex (**d**), and midbrain (**e**) and cerebellum (**f**) from different mice *RELN* WT/WT, *RELN* WT/H3448R and *RELN*

H3448R/H3448R male mice at 16 weeks of age. Data is showing that there is not significant change in pGSK3β signaling in the presence of the H3448R variant at this age. All data is presented as average of n = 3 mice ± s.e.m.

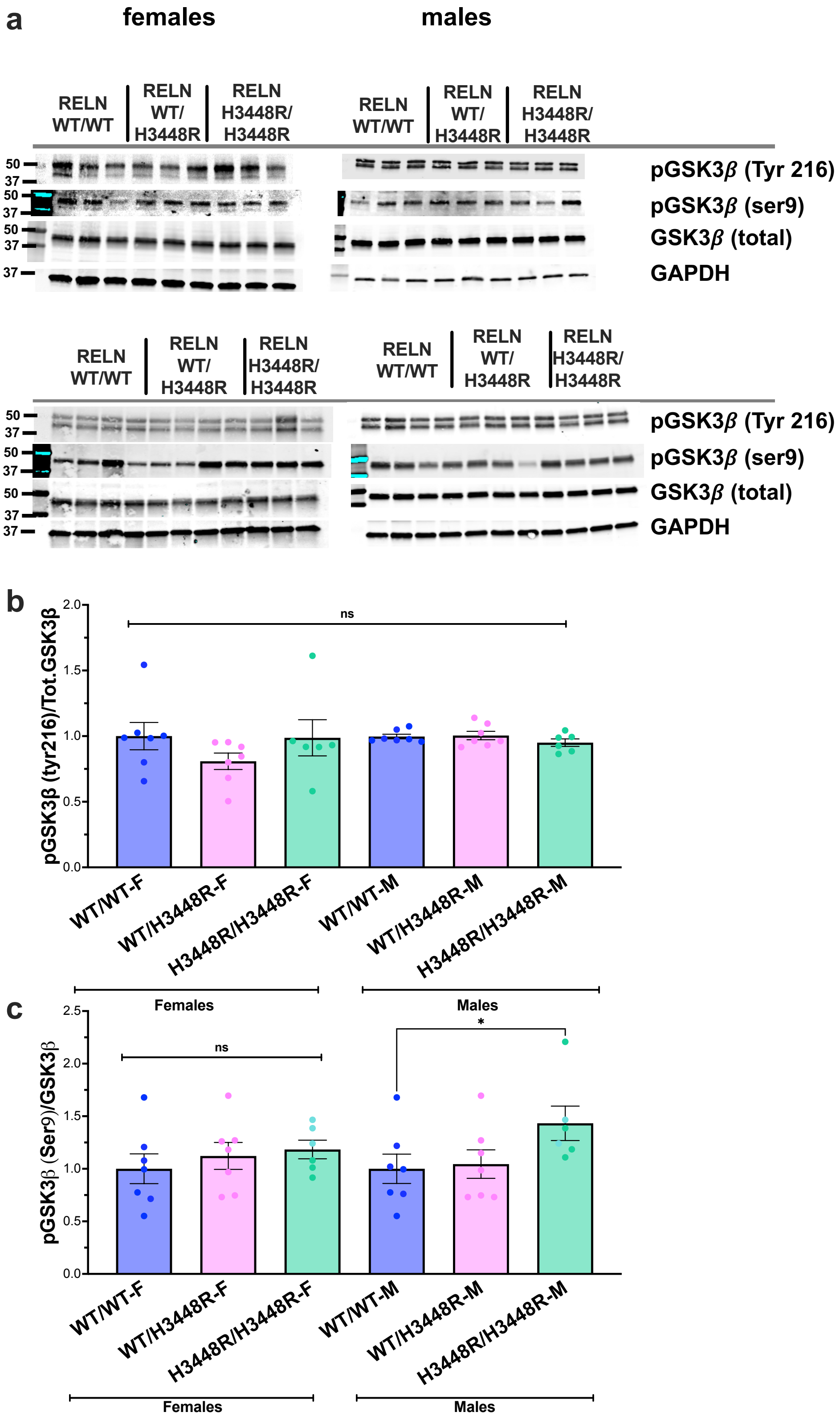

Supplementary Figure 12. Analyses of GSK3β levels in the cerebellum of the H3448R *RELN* Tg mice show increased levels of pGSK3β (Ser9) in male homozygous mice and not in female mice. a, Representative blots of pGSK3β(Ser9), pGSK3β(Tyr216), total GSK3β and

GAPDH levels in the *RELN*-H3448R Tg mouse model of both males and females at 6 (top blots, n = 3 mice) and 12 months of age (bottom blots, n = 4 mice, *RELN* WT/WT and *RELN* WT/H3448R; n = 3 mice, *RELN* H3448R/H3448R). (**b, c**) Quantifications of the western blotting data presented in panel a for GSK3β (Tyr216) and (Ser9) respectively and confirmed a significant increase of pGSK3β (Ser9) in male homozygous (*p = 0.0332, one-way ANOVA, followed by Fisher's LSD test for multiple comparisons), n1 = n2 = 6, DF = 34, t = 2.220). All data is presented as average ± s.e.m.

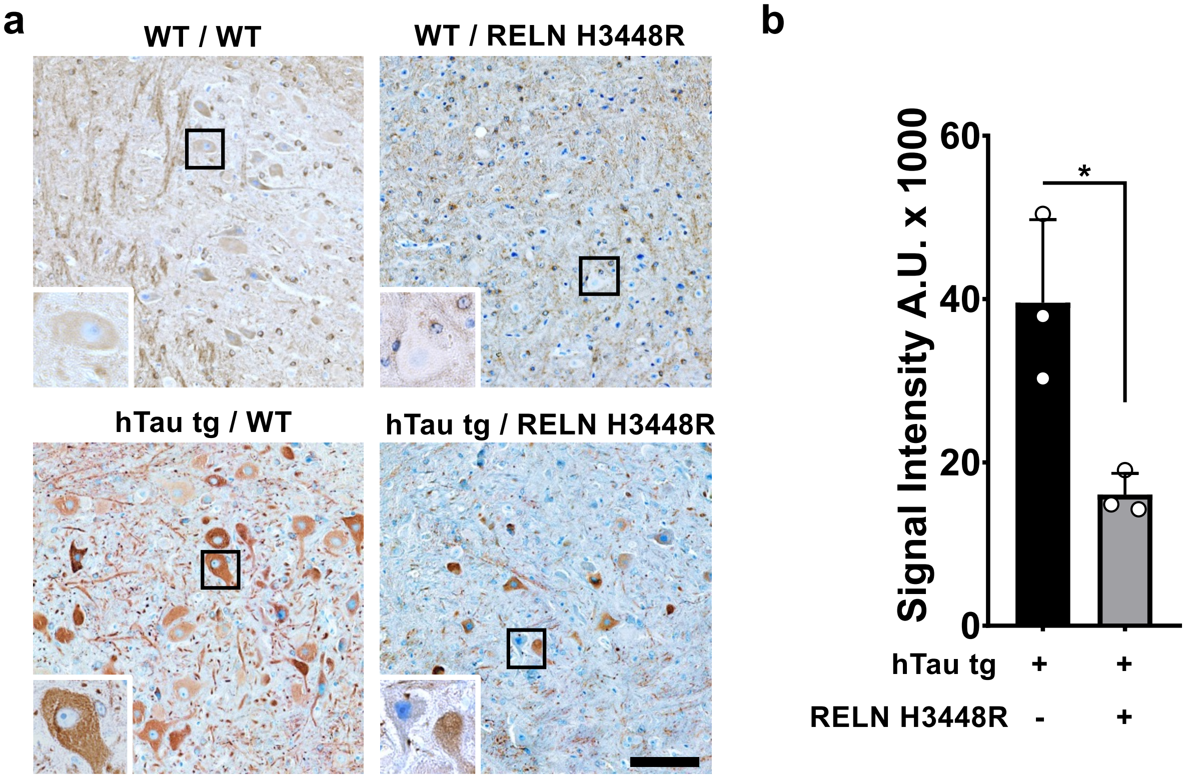

Supplementary Figure 13. Tau pathology characterization in the medulla oblongata of hTau tg / *RELN* H3448R mice. a, Representative IHC images from the hippocampus of WT / WT, WT / *RELN* H3448R, hTau tg / WT and hTau tg / *RELN* H3448R mice stained with pTau T205 antibody. hTau tg / WT mice showed neurofibrillary tangles and neuropil threads in the trigeminal nucleus, while hTau tg / *RELN* H3448R showed Tau pathology to a lesser degree (soma of an affected neuron depicted with a dotted line). Mice with the *RELN* allele are homozygous. Bar = 100 μm. **b,** Bar graph for pTau T205 signal intensity values in hTau tg / WT (n = 3 mice) and hTau tg / *RELN* H3448R (n = 3 mice). The latter showed significantly less signal intensity values. p = 0.018*, two-sided Student's T. Test. Error bars represent standard deviation from mean.

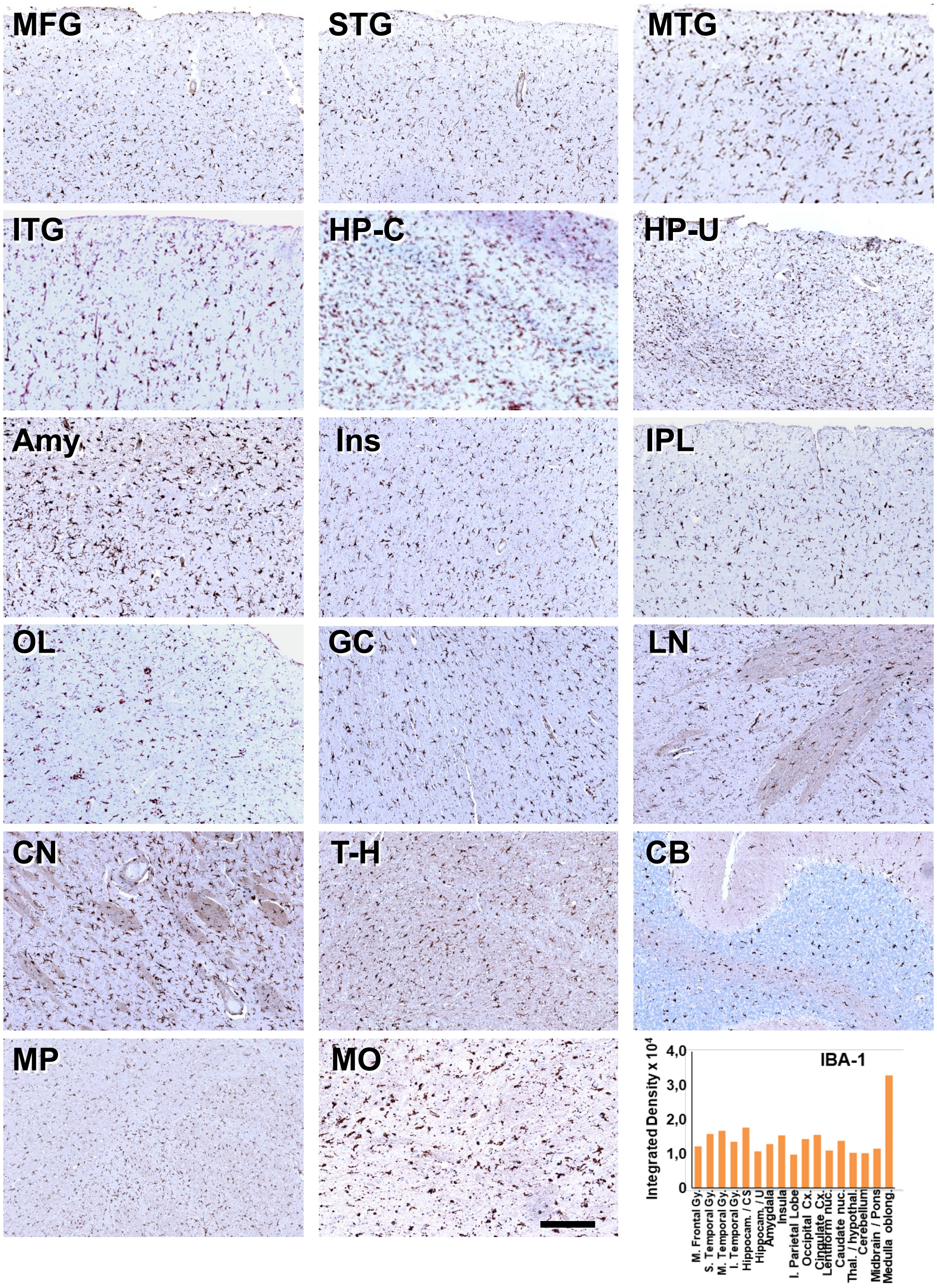

Supplementary Figure 14. IHC staining for Iba1 (microglia). MFG = Medial frontal gyrus, STG = Superior temporal gyrus, MTG = Medial temporal gyrus, ITG = Inferior temporal gyrus, HP-C = Hippocampus/collateral sulcus, HP-Uncus = Hippocampus / uncus, Amy = Amygdala, Ins = Insula, IPL = Inferior parietal lobe, OL = Occipital lobe, GC = Gyrus cinguli, LN = Lentiform nucleus, CN = Caudate nucleus, T-H = Thalamus/Hypothalamus, CB = Cerebellum, MP = Midbrain/pons, MO = Medulla oblongata. Scale bar = 100 μm.

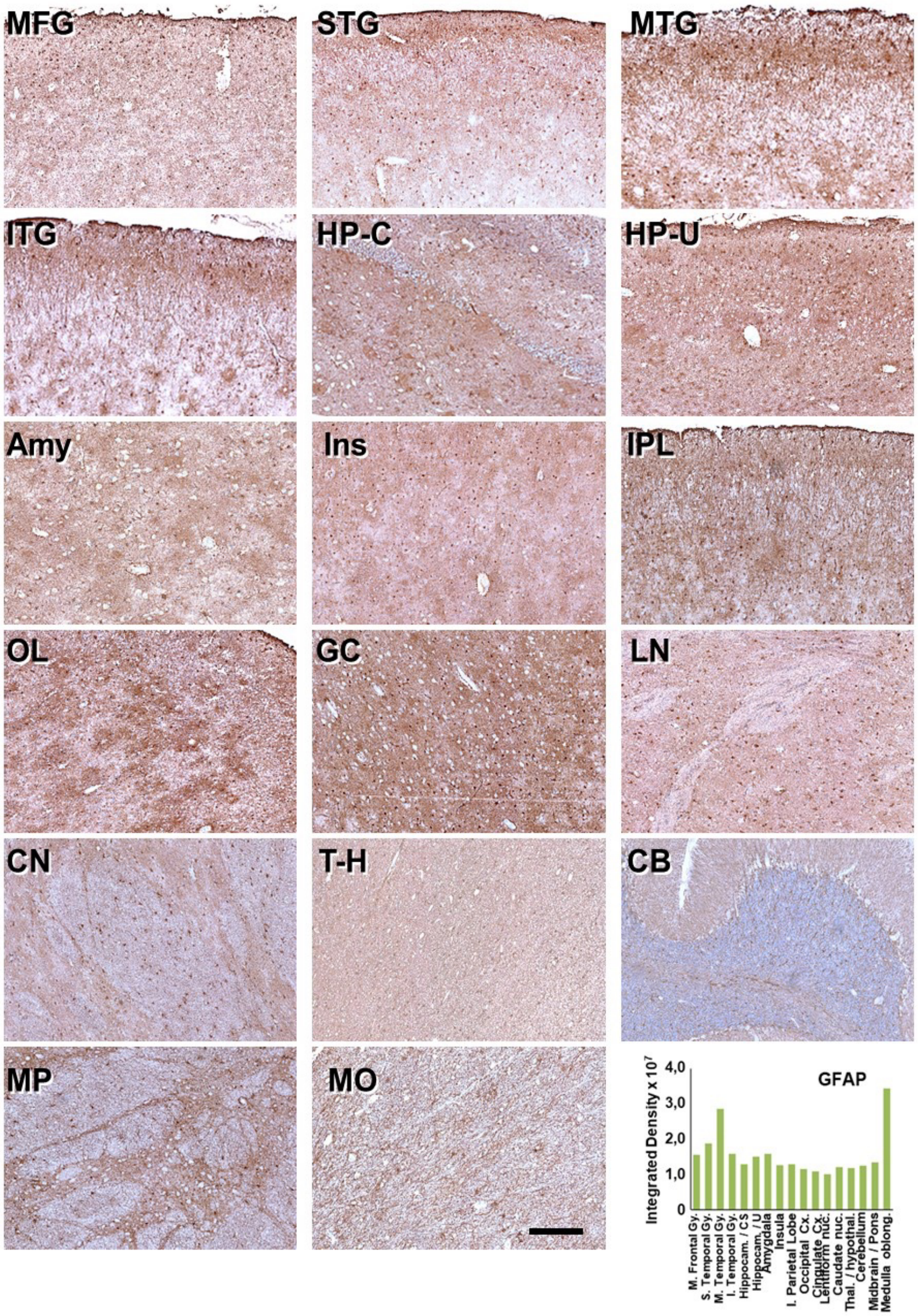

Supplementary Figure 15. IHC staining for GFAP (astrocytes). MFG = Medial frontal gyrus, STG = Superior temporal gyrus, MTG = Medial temporal gyrus, ITG = Inferior temporal gyrus, HP-C = Hippocampus/collateral sulcus, HP-Uncus = Hippocampus/uncus, Amy = Amygdala, Ins = Insula, IPL = Inferior parietal lobe, OL = Occipital lobe, GC = Gyrus cinguli, LN = Lentiform nucleus, CN = Caudate nucleus, T-H = Thalamus/Hypothalamus, CB = Cerebellum, MP = Midbrain/pons, MO = Medulla oblongata. Scale bar = 100 μm.

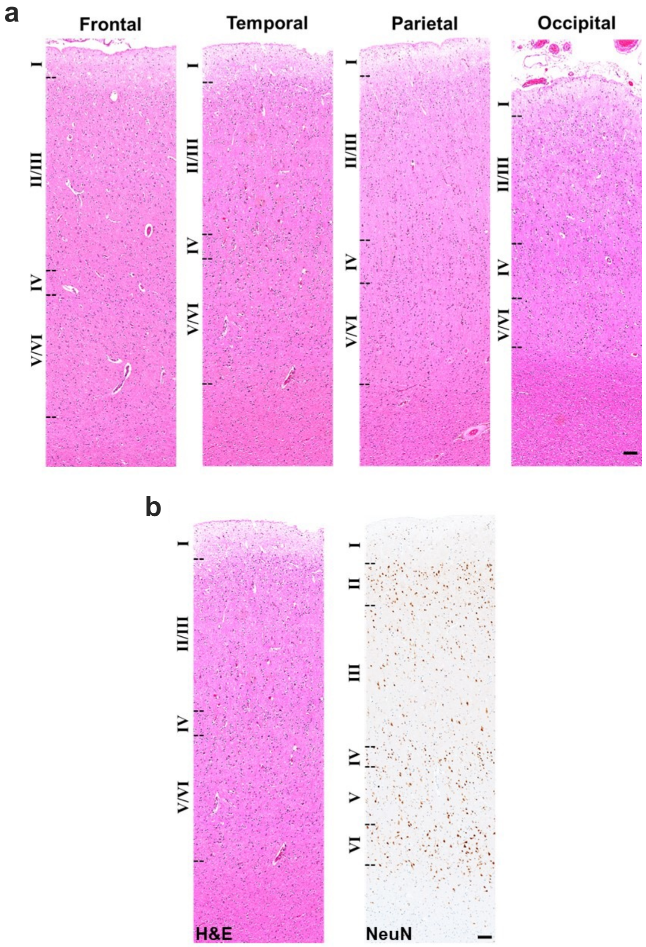

Supplementary Figure 16. HE staining. a, Hematoxylin and Eosin (HE) staining of frontal, temporal, parietal, and occipital cortices depicting neuronal layers architecture. **b**, Comparison between HE and Hexaribonucleotide Binding Protein-3 (NeuN) staining in the temporal cortex allowing further identification of neuronal layers. There was no evidence of defects of neuronal organization in any area investigated. Scale bar = 100μm.

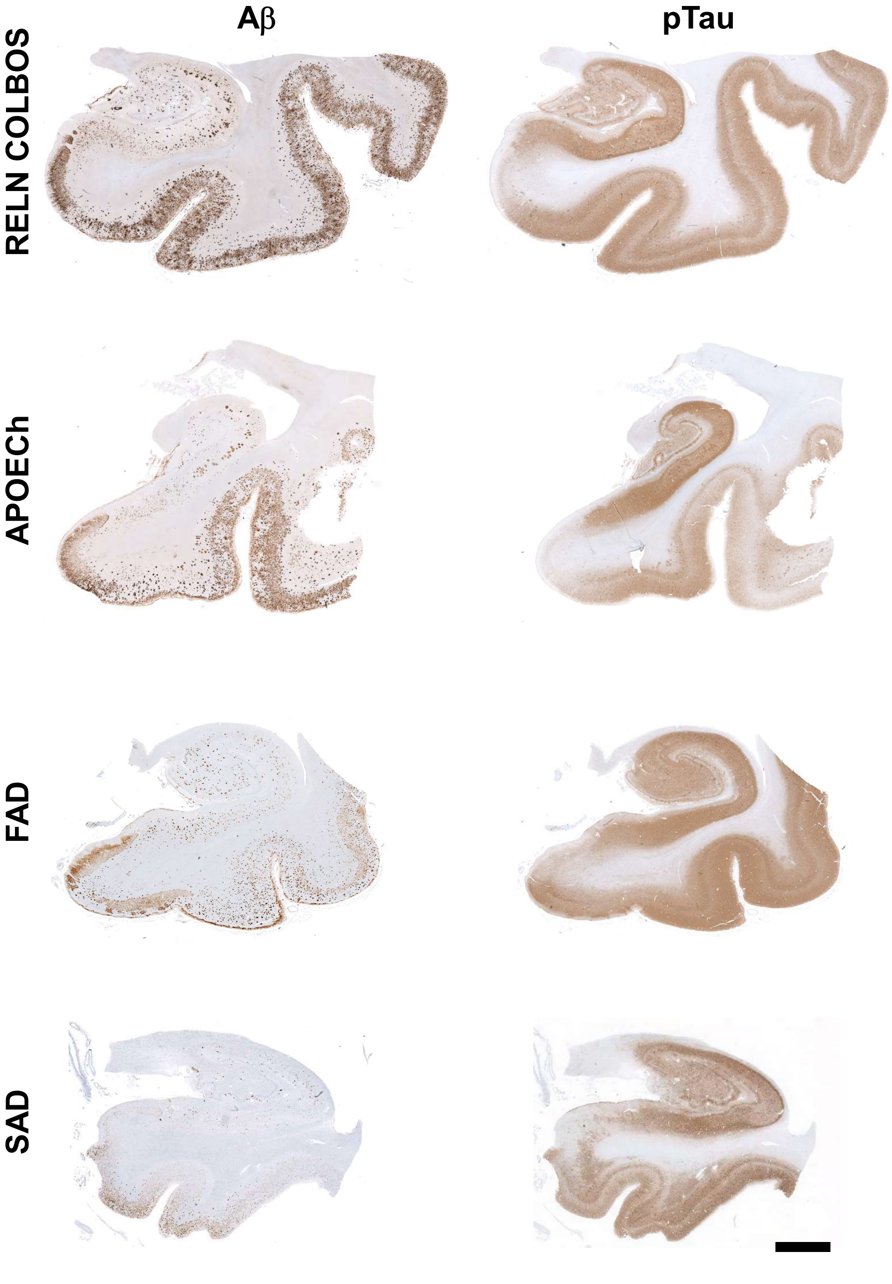

Supplementary Figure 17. Comparisons of Aβ and ptau levels in the limbic structures. Immunohistochemical staining for Aβ and ptau of limbic structures of the temporal cortex, including hippocampus, entorhinal cortex and parahippocampal gyrus of the *RELN-COLBOS* case, the *APOECh* case, a representative *PSEN1 E280A* FAD case and a representative SAD case. Scale bar = 3 mm.

Supplementary table 10. Demographic information of the cases used for neuropathological comparisons.

Case	Group	Genotype	Age at onset of dementia	Age of death	Disease duration	Braak	Thal	CERAD
1	FAD - protected	PSEN1 E280A, RELN H3447R	72	74	2	6	5	C
2	FAD - protected	PSEN1 E280A, APOE R136S	75	77	2	6	5	C
3	FAD	PSEN1 E280A	43	49	6	6	5	C
4	FAD	PSEN1 E280A	58	69	11	6	5	C
5	FAD	PSEN1 E280A	41	56	15	6	5	C
6	FAD	PSEN1 E280A	53	58	5	6	5	C
7	FAD	PSEN1 E280A	49	57	8	6	5	C
8	SAD	N.A.	75	85	10	6	5	C
9	SAD	N.A.	81	94	13	5	5	C
10	SAD	N.A.	79	90	11	6	5	C
11	SAD	N.A.	56	70	14	6	5	C

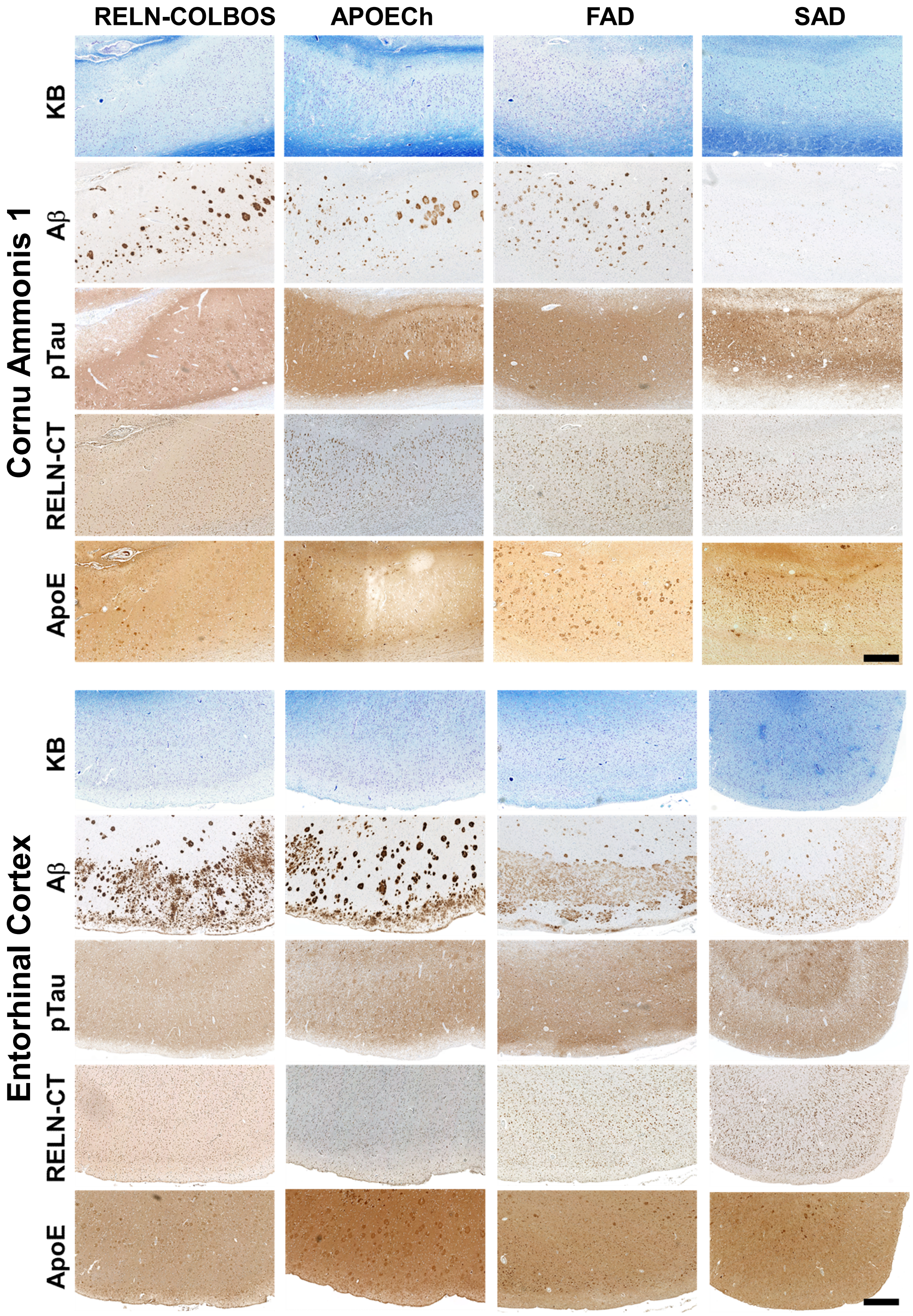

Supplementary Figure 18. Immunohistochemical comparisons of the entorhinal cortex and *Cornu Ammonis*. Representative pictures of Kluver Barrera (KB), Aβ, ptau, RELN-CT, and ApoE staining in the *Cornu Ammonis* 1 and Entorhinal cortex of the *RELN-COLBOS* case, the APOECh case, a representative *PSEN1* E280A FAD case and a representative SAD case. Scale bar = 500 μm.

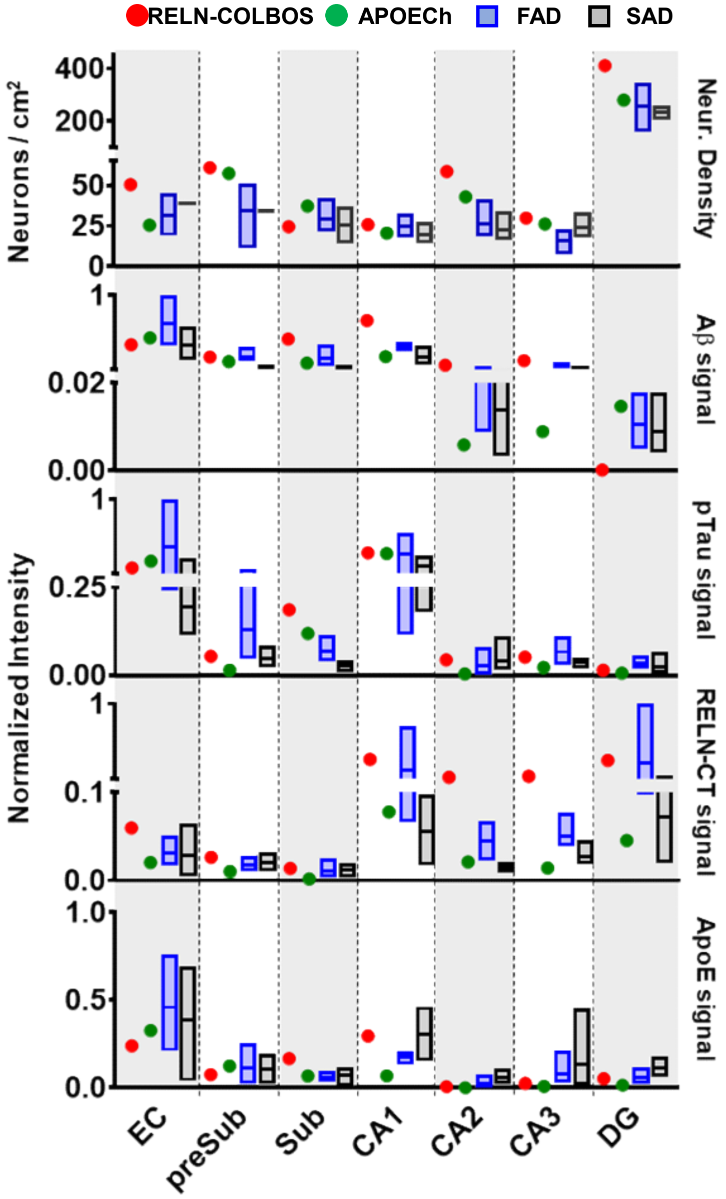

Supplementary Figure 19. Quantitative analyses of the neuronal density. Bar graph representations of neuronal density measurements and signal intensity measurements in Entorhinal cortex (EC), Presubiculum (preSub), Subiculum (Sub), CA1, CA2, CA3 and Dentate Gyrus (DG) for Aβ, ptau, RELN-CT and ApoE staining for the *RELN-COLBOS* case, the *APOECh* case, *PSEN1* E280A FAD cases (n = 5) and SAD cases (n = 4). The *RELN-COLBOS* case shows higher RELN-CT signal intensity than all FAD cases, including the *APOECh* one.

SUPPLEMENTARY METHODS

Plasma Nfl Assay

Plasma neurofilament light (NfL) analysis was conducted by the Clinical Neurochemistry Laboratory at Sahlgrenska University Hospital (Mölndal, Sweden) as previously reported[2]. The experimenter was masked to clinical or genetic data of the proband, to avoid any bias.

Genome sequencing

Whole genome sequencing analysis called 4.4 million variants from the case's genome.
We used the reference genome hs37d5 (ftp://ftp.1000genomes.ebi.ac.uk/vol1/ftp/technical/reference/phase2_reference_assembly_sequence/hs37d5.fa.gz). Subsequent genome analysis was done with Edico Genome Dragen Pipeline (Version - SW: 01.011.231.02.05.01.40152, HW: 01.011.231), bcftools 1.9 (http://samtools.github.io/bcftools/), Ensembl VEP v94 (https://uswest.ensembl.org/info/docs/tools/vep/index.html), Gnomad r2.0.1 (http://gnomad.broadinstitute.org/downloads), bcbio nextgen tool suite v1.1.2 (https://github.com/bcbio/bcbio-nextgen), Exomiser (v10.1.0), Cartagenia version 5.0 (https://www.genomeweb.com/resources/new-product/cartagenia-bench-lab-50), HGMD: Qiagen HGMD® Professional Database 2018.2, OMIM, ExAC: ExAC release 0.3, and GnomAD: latest online version (http://gnomad.broadinstitute.org/). The variants went through multiple filters using Genomiser (Exomiser v11.0.01): population allele frequency < = 2%, variant effect filter that exclude intergenic, untranslated regions (UTR), and non-coding intronic regions, regulatory feature filter removes all non-regulatory, non-coding variants over 20 Kb from a known gene, and hiPhive prioritizer phenotype gene priority score > = 0.401. 6,779 variants passed filtering and were ranked by the composite Exomiser scores from gene-phenotype scores and variant pathogenicity scores, and the compatibility with the mode of inheritance. The top three ranked variants were manually reviewed for pathogenicity. We also manually searched a list of 23 genes previously associated to Alzheimer's including *AAGAB*, *ABCC8*, *AKT2*, *APOE*, *APP*, *BEAN1*, *GATA1*, *GCK*, *HMGA1*, *HNF1B*, *HNF4A*, *LDB3*, *PAX4*, *PSEN1*, *PSEN2*, *ABCA7*, *SORL1 CACNA1G*, *HFE*, *MPO*, *NOS3*, *PLAU*. The genes variants from WGS were extracted and annotated in the Alissa Interpret platform for independent pathogenicity interpretation (Agilent, Santa Clara, CA). We calculated "C scores" using Combined Annotation Dependent Depletion CADD[56,57], to contrast pathogenicity of human-derived vs. simulated variants. The regulatory Mendelian mutation (ReMM) framework uses machine learning techniques to train a classifier to predict the potential of an arbitrary position in the non-coding genome to cause a Mendelian disease if mutated[44].

Single cell RNA sequencing

We used a Ficoll gradient to isolate peripheral blood mononuclear cells and these cells were analyzed by scRNA sequencing using a protocol previously published [2]. We used Cell Ranger software suite v3.0.2 for demultiplexing, barcode processing, transcript counting and clustering analysis. Seurat R-package was used for downstream analysis and feature-barcoding.
For clustering, Cell Ranger v3.0.2 was used and data was viewed using Loupe Cell Browser. The SCTransform algorithm was used for the gene-cell count, and non-linear dimensional reduction was done with RunUMAP with the UMAP algorithm.

RELN genotyping by Sanger DNA sequencing
The DNA was first extracted using Gentra Puregene Kit (Qiagen). 50 μL of the obtained samples (both *RELN* H3447R and *RELN* H3447 carriers) were amplified via polymerase chain reaction (PCR) using one μL of ten μM primers (forward:5'- GTCCCAGCCTTTAGTTCCT-3'; reverse: 3'-CAACTTTCACGGACACATCAA-5') pre-mixed in PCR Master Mix 2X (K0171, Thermo Fisher) using the following protocol: 3 minutes at 94 ºC for initial denaturation, 33 cycles, 30 seconds at 94 ºC, 35 seconds at 62 ºC for annealing, 35 seconds at 72 ºC for the elongation, five minutes at 72 ºC for final extension. Horizontal electrophoresis was done at 100 V using 1.5% Agarose gel in TAE buffer (Tris-acetate EDTA, T8280-1L, Sigma Aldrich) and mixed with GelRed (41003-T, Biotium). Fluorescence of positive bands was detected via Bio-RAD Molecular Imager Gel Doc XR+ and acquired via Image Lab software (ver. 6.0.1, Biorad). Purification of the amplified DNA was done using QIAquick Gel Extraction kit (Qiagen) and sequenced by MGH CCIB DNA core using the 3730xl sequencer (Applied Biosystems) as previously published [2].

ELISA
Enzyme-linked immunosorbent assay (ELISA) was used to quantify changes in binding of RELN variants (media-derived full-length or CTR-RELN) to either VLDLr or ApoEr2 following an optimized version of a previously published protocol [58]. Briefly, ELISA strips (DY008, R&D Systems) were coated with 1 ng/μL (100 μL/well) of VLDLr (8444-VL, R&D) or ApoEr2 (TP320903, OriGene) receptors diluted in 25 mM Tris HCl, 140 mM NaCl, 27 mM KCl, 2 mM $CaCl_2$, pH 7.4 (TBS-C buffer). After 18 h at 4 °C incubation, plates were blocked one h with 3 % BSA (22070008-6, Bioworld), 0.05% Tween-20 (Sigma Aldrich) TBS-C buffer. We assessed binding via incubation with 100 μL/well of serial dilutions of recombinant RELN protein (Ser1221-Gln2666, 8546-MR-050, R&D) or RELN variants in TBS-C buffer for one h at R.T. For detection, we used goat anti-mouse RELN primary antibody (LS-C793521-100, LS-Bio, 1:2,000, 100 μL/well) as primary antibody for 1 h at R.T. and (1:10,000; 100 μL/well, donkey anti-goat IgG H&L, HRP, ab6885, Abcam) as secondary antibody for 30 min. at R.T. Between each of the previous steps, plates were washed 4 times with TBS-C buffer (200 μL/well). Plates were washed three times prior to initiating the colorimetric reaction (DY008, R&D Systems). Stop solution (50 μL/well, DY008, R&D Systems) was added upon 5 min. incubation and the absorbance of the samples was measured at 450 nm using the Synergy 2 microplate reader (BioTek Instruments). Processing and analysis of the data was done using Gen5 1.11 software and GraphPad Prism 8 v8.1.1, respectively.

A plasmid encoding for the full-length murine recombinant RELN that was a gift from T. Curran via Addgene (plasmid no. 122444 [14]. The plasmid was subsequently mutagenized to obtain the H3448R mutation homologous of human RELN-H3447R as fee-for-service by Custom DNA Constructs. For ELISA, we produced WT and *RELN-COLBOS* in Flp-In T-REx 293 mammalian cells (catalog no. R78007, Thermo Fisher Scientific) via transient transfection and used them for receptor binding assays via ELISA. All constructs were overexpressed in Flp-In T-REx 293 mammalian cells via transient transfection using Lipofectamine 2000 according to the manufacturer's instructions (catalog no. 11668030, Thermo Fisher Scientific). Five hours after transfection, Opti-MEM was used to collect conditioned medium. Cells were incubated for 24 h and supernatant was collected and cleared from cell debris by centrifugation for 3 min, 1,800 g at room temperature.

Circular Dichroism

For CD and NMR measurements, recombinant CTR-RELN WT peptides were produced as a fee-for service (Creative Biostructure) by cloning the peptide sequence into a pET28a expression vector and subsequently produced by transformation of the plasmid in *E. coli* and overnight (ON) expansion in 10 mL Luria-Bertani (LB) medium at 37°C. To label peptides for NMR, the cell culture was inoculated ON in 1000 mL of 15N-labelled M9 medium (1:100 dilution). The CD signal was recorded at the wavelengths ranging from 190 to 260 nm at 0.1 nm intervals using a 1 mm pathlength quartz cuvette (Chirascan-Plus CD Spectrometer; Applied Photophysics, UK). The samples were scanned at 50 nm/min with a 1 nm bandwidth and a 2 second integration time. Data was plotted as the average of four spectra, Data was deconvoluted using the online deconvolution software BeStSel (https://bestsel.elte.hu/).

2D Nuclear Magnetic Resonance (NMR) structures and in silico analyses

The final NMR sample consisted of 50% (V/V) H2O, 50% (V/V) of 98% pure 2,2,2-Trifluoroethanol-d2 (CF3CD2OH), 0.25 mM DSS (4,4-dimethyl-4-silapentane-1-sulfonic acid, dissolved in H2O) as the internal standard, and ~3.3 mM reelin peptide. 1H TOCSY (80ms spin lock time), NOESY (200 ms mixing time), 1H-15N HSQC, 1H-13C HSQC experiments were performed at 25°C on an Agilent 600 MHz NMR Spectrometer (DD2). Data was analyzed with NMRPipe and CCPNmr 2.4.2; structures were calculated with Cns 1.2.1 and aria 2.3.2. In total 200 structures were calculated, and the 20 lowest energy structures were selected. NMR structure data, the orientation of basic amino acids were assessed in PyMol Version 2.3.3 (https://pymol.org/). NMR structure has deposited to PDB.org (DOI: 10.2210/pdb8g21/pdb). For CTR-RELN H3447R, the model was determined by a homology-based model of WT CTR-RELN that was calculated by Swiss-Model. To assess RELN peptide docking with heparin, the WT RELN NMR structure was uploaded to ClusPro 2.0 (https://cluspro.org/), and "Heparin Mode" was used to model interactions between the WT Reelin peptide and heparin. Polar contacts of the lowest energy docking output were defined by the PyMol software as contacts within 3 Å through the "Measurement Mode" function. The movies included in supplement shows the 20 lowest energy NMR structures shown sequentially using the PyMol film editor.

SPR Assay

Surface plasmon resonance assay (SPR) was used for performing binding kinetics using a Biacore 3000 Instrument (GE Healthcare) at 25 °C following previously published protocols [59 24] as fee-for-service by Precision Antibody (Maryland, USA). Biotin-labeled heparin (B9806, Sigma Aldrich) was covalently linked to streptavidin-coated chip and unoccupied sites were blocked with biocytin. Antigen was flowed over the chip using a range of single analyte concentrations prepared in DPBS buffer and using a flow rate of 30 µL/min. Binding of antigen to the ligand was monitored in real time to obtain on (ka) and off (kd) rates. The equilibrium constant (K_D) was calculated from the observed ka and kd. Accuracy of the SPR analysis was determined via Chi square ($\chi 2$) analysis as described in the statistical analysis section. We included in the analyses the WT and H3447R peptides and peptides with the hypothetical H3447D change (RKQNYMMNFSRQHGLRDFYNRRRRSLRRYP) and the H3447K change (RKQNYMMNFSRQHGLRKFYNRRRRSLRRYP), also produced, and purified as fee-for-service by Innovagen (Sweden).

Cresyl Violet staining of mouse brain

We collected brains from five to six months old male mice wild type, heterozygous, homozygous for the H3448R *RELN* variant. Brains were fixed for 48 h in 10% buffered neutral formalin solution (HT501128-4L, Sigma-Aldrich, MO), and 0.6 mm tick sagittal sections embedded in paraffin. 15 mm tick sections from each genotype were deparaffinized by incubating the slides in an oven at 59 °C for at least two hours. Slides were incubated twice for three minutes each in 100% pure xylene (1.94600, Sigma-Aldrich, MO). Subsequently, the tissue was hydrated by immersion in serial dilutions of ethanol (459836, Sigma-Aldrich, MO) starting at 95% v/v ending with 50% v/v. Neuronal staining was obtained using 0.1% Cresyl violet acetate solution (26089-01, Electron Microcopy Science, PA) at pH 3.0 for three minutes, rinsed with distilled water, and dehydrated in pure ethanol for on minute. Samples were rinsed in xylene and mounted with Permount® mounting media (SP15-100, Fisher Scientific, NH). Brightfield images were obtained using a NanoZoomer®-SQ slide scanner (C13140, Hamamatsu, Japan) with a 40x objective. We recorded image fields of a 0.05 mm^2 area in the granular layer of cerebellum. At least three slides per specimen and three images per slice were used for image analysis and quantification. All statistical comparisons were conducted on Graphpad Prism 9 as indicated in the dedicated section.

Immunoprecipitation Details

Mouse frontal cortex tissue was homogenized by two 15 second pulses in ice cold M-PER protein extraction reagent (78503, Thermo Fisher) supplemented with phosphatases and proteases inhibitors using a homogenizer as described in the previous paragraph. 100 µg of total proteins was pulled down using either anti-phosphotyrosine magnetic beads (clone 4G10, 16-282, Millipore), anti-total Dab1 agarose beads (clone G-5, sc-271136 AC, Santa Cruz) and anti-normal mouse IgG isotype control agarose beads (SC-2343, Santa Cruz). Prior immunoprecipitating the sample over night at 4 °C, 10 % was removed and analyzed as input. Beads were washed in PBS upon collecting the unbound fraction and subsequently boiled in 4X reducing sample buffer for six minutes to allow the release of the immune precipitated proteins.

Sequencing analysis via mass spectrometry

Immunoprecipitated fractions obtained using the anti-Dab1 conjugated beads were electrophoretically separated on a 10 % acrylamide precast gel (Biorad) and stained using blue Coomassie (Thermo Fisher). Excised gel bands were analyzed as a fee-for-service at the Taplin Biological Mass Spectrometry Facility (Harvard Medical School, Boston, MA). Gel bands were subsequently dehydrated in acetonitrile followed to speed-vac. Rehydration of the bands were obtained in a solution of 50 mM ammonium bicarbonate supplemented with 12.5 ng/µL modified sequencing-grade trypsin (Promega, Madison, WI) at 4 ºC. Samples was washed with 50 mM ammonium bicarbonate solution. Upon overnight incubation a 37ºC, protein extraction was obtained by removing the ammonium bicarbonate solution. Proteins were washed in a of 50% acetonitrile and 1 % formic acid [60]. Reconstituted samples sequenced using nano-scale reverse-phase HPLC capillary column upon gradient elution using acetonitrile and formic acid via an electrospray ionization-LTQ Orbitrap Velos Pro ion-trap mass spectrometer (Thermo Fisher Scientific, Waltham, MA). Protein-specific fragment ions sequence was analyzed using the know peptide sequences using Sequest[61] (Thermo Fisher Scientific, Waltham, MA).

References

34. Torres, V.L., *et al.* Normative data stratified by age and education for a Spanish neuropsychological test battery: Results from the Colombian Alzheimer's prevention initiative registry. *Appl Neuropsychol Adult*, 1-15 (2019).
35. Hong, S.E., *et al.* Autosomal recessive lissencephaly with cerebellar hypoplasia is associated with human RELN mutations. *Nat Genet* **26**, 93-96 (2000).
36. Eriksson, S.H., *et al.* Persistent reelin-expressing Cajal-Retzius cells in polymicrogyria. *Brain* **124**, 1350-1361 (2001).
37. Quiroz, Y.T., *et al.* Plasma neurofilament light chain in the presenilin 1 E280A autosomal dominant Alzheimer's disease kindred: a cross-sectional and longitudinal cohort study. *The Lancet Neurology* **19**, 513-521 (2020).
38. Neufeld, G., Kessler, O. & Herzog, Y. The interaction of Neuropilin-1 and Neuropilin-2 with tyrosine-kinase receptors for VEGF. *Adv Exp Med Biol* **515**, 81-90 (2002).
39. Turk, L.S., Currie, M.J., Dobson, R.C.J. & Comoletti, D. Structure of Reelin repeat 8 and the adjacent C-terminal region. *Biophys J* **121**, 2526-2537 (2022).
40. Nogi, T., Yasui, N., Hattori, M., Iwasaki, K. & Takagi, J. Structure of a signaling-competent reelin fragment revealed by X-ray crystallography and electron tomography. *Embo j* **25**, 3675-3683 (2006).
41. Yasui, N., *et al.* Structure of a receptor-binding fragment of reelin and mutational analysis reveal a recognition mechanism similar to endocytic receptors. *Proc Natl Acad Sci U S A* **104**, 9988-9993 (2007).
42. Lortat-Jacob, H., Grosdidier, A. & Imberty, A. Structural diversity of heparan sulfate binding domains in chemokines. *Proc Natl Acad Sci U S A* **99**, 1229-1234 (2002).
43. Wardell, M.R., Brennan, S.O., Janus, E.D., Fraser, R. & Carrell, R.W. Apolipoprotein E2-Christchurch (136 Arg----Ser). New variant of human apolipoprotein E in a patient with type III hyperlipoproteinemia. *J Clin Invest* **80**, 483-490 (1987).
44. Smedley, D., *et al.* A Whole-Genome Analysis Framework for Effective Identification of Pathogenic Regulatory Variants in Mendelian Disease. *Am J Hum Genet* **99**, 595-606 (2016).
45. Lalli, M.A., *et al.* Whole-genome sequencing suggests a chemokine gene cluster that modifies age at onset in familial Alzheimer's disease. *Mol Psychiatry* **20**, 1294-1300 (2015).
46. Karczewski, K.J., *et al.* The mutational constraint spectrum quantified from variation in 141,456 humans. *Nature* **581**, 434-443 (2020).
47. Carvajal-Carmona, L.G., *et al.* Strong Amerind/white sex bias and a possible Sephardic contribution among the founders of a population in northwest Colombia. *Am J Hum Genet* **67**, 1287-1295 (2000).
48. Lalli, M.A., *et al.* Origin of the PSEN1 E280A mutation causing early-onset Alzheimer's disease. *Alzheimers Dement* **10**, S277-S283.e210 (2014).
49. de Bergeyck, V., *et al.* A truncated Reelin protein is produced but not secreted in the 'Orleans' reeler mutation (Reln[rl-Orl]). *Brain Res Mol Brain Res* **50**, 85-90 (1997).
50. Yasui, N., Nogi, T. & Takagi, J. Structural basis for specific recognition of reelin by its receptors. *Structure* **18**, 320-331 (2010).

51. Dinkel, F., *et al.* Decreased Deposition of Beta-Amyloid 1-38 and Increased Deposition of Beta-Amyloid 1-42 in Brain Tissue of Presenilin-1 E280A Familial Alzheimer's Disease Patients. *Front Aging Neurosci* **12**, 220 (2020).
52. Heppner, F.L., Ransohoff, R.M. & Becher, B. Immune attack: the role of inflammation in Alzheimer disease. *Nat Rev Neurosci* **16**, 358-372 (2015).
53. Krbot, K., *et al.* Distinct microglia profile in Creutzfeldt–Jakob disease and Alzheimer's disease is independent of disease kinetics. *Neuropathology* **38**, 591-600 (2018).
54. Guerrini, R. & Parrini, E. Neuronal migration disorders. *Neurobiol Dis* **38**, 154-166 (2010).
55. Einstein, G., *et al.* Intraneuronal ApoE in human visual cortical areas reflects the staging of Alzheimer disease pathology. *J Neuropathol Exp Neurol* **57**, 1190-1201 (1998).
56. Kircher, M., *et al.* A general framework for estimating the relative pathogenicity of human genetic variants. *Nature Genetics* **46**, 310-315 (2014).
57. Rentzsch, P., Witten, D., Cooper, G.M., Shendure, J. & Kircher, M. CADD: predicting the deleteriousness of variants throughout the human genome. *Nucleic Acids Res* **47**, D886-d894 (2019).
58. Bajari, T.M., Strasser, V., Nimpf, J. & Schneider, W.J. LDL receptor family: isolation, production, and ligand binding analysis. *Methods* **36**, 109-116 (2005).
59. Day, E.S., Capili, A.D., Borysenko, C.W., Zafari, M. & Whitty, A. Determining the affinity and stoichiometry of interactions between unmodified proteins in solution using Biacore. *Anal Biochem* **440**, 96-107 (2013).
60. Shevchenko, A., Wilm, M., Vorm, O. & Mann, M. Mass spectrometric sequencing of proteins silver-stained polyacrylamide gels. *Anal Chem* **68**, 850-858 (1996).
61. Peng, J. & Gygi, S.P. Proteomics: the move to mixtures. *J Mass Spectrom* **36**, 1083-1091 (2001).

Source data:
Full unprocessed scans for Supplementary Figure 8a (GPDH uncropped blots).

Females (left)

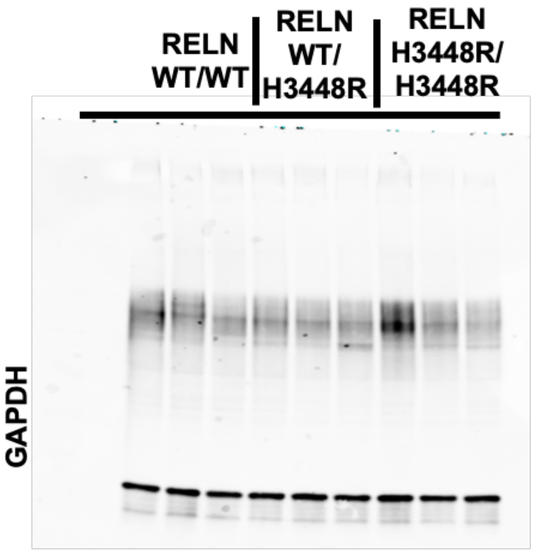

Females (right)

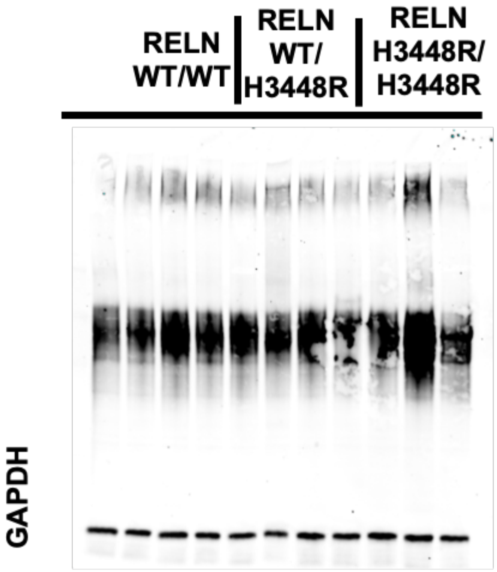

Males (left)

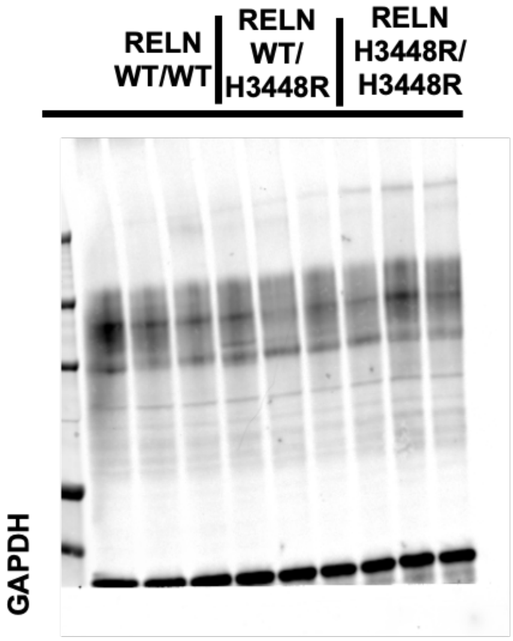

Males (right)

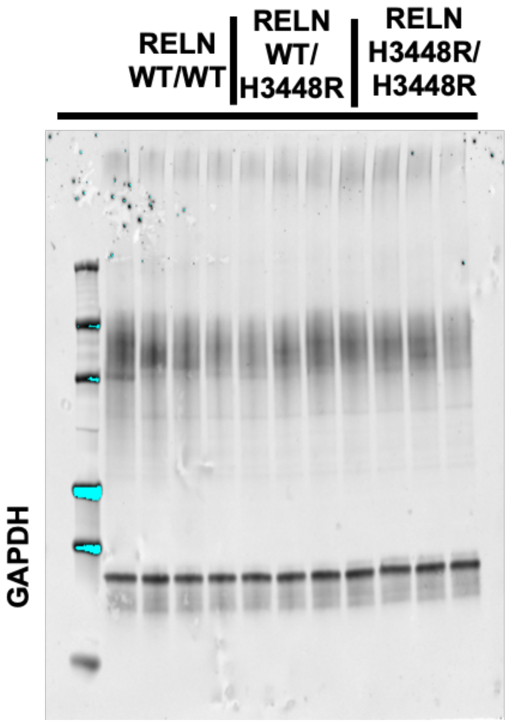

Full unprocessed scans for Supplementary Figure 9 (GAPDH uncropped blots).

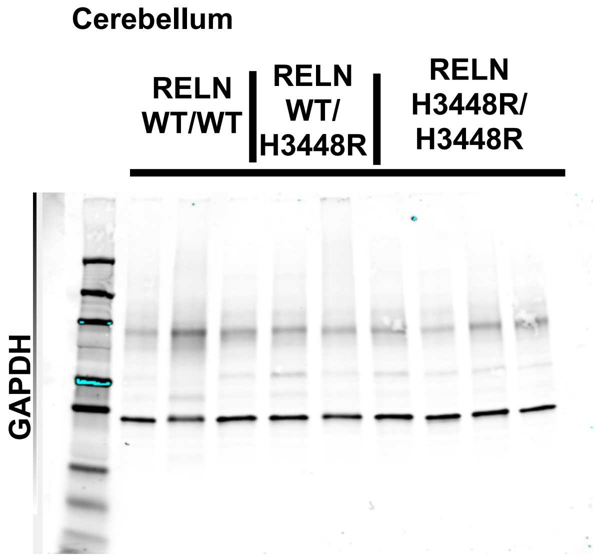

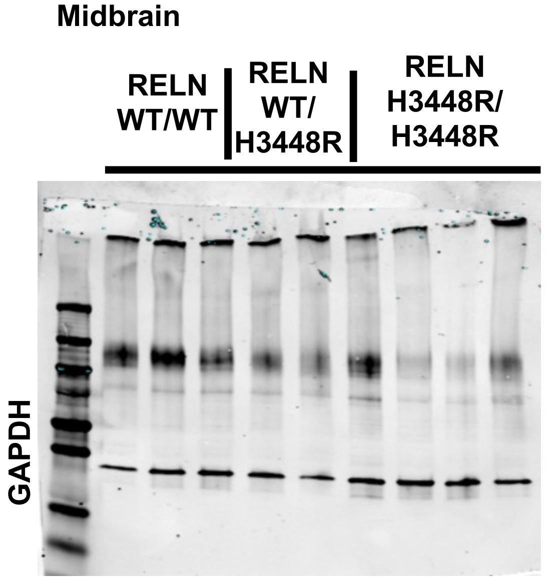

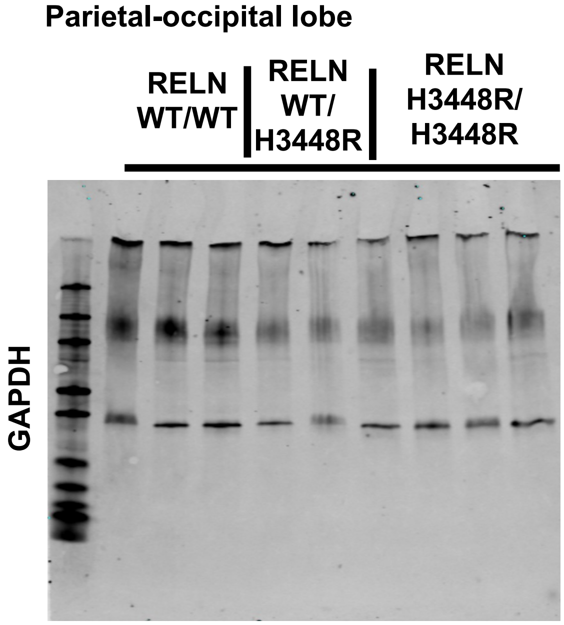

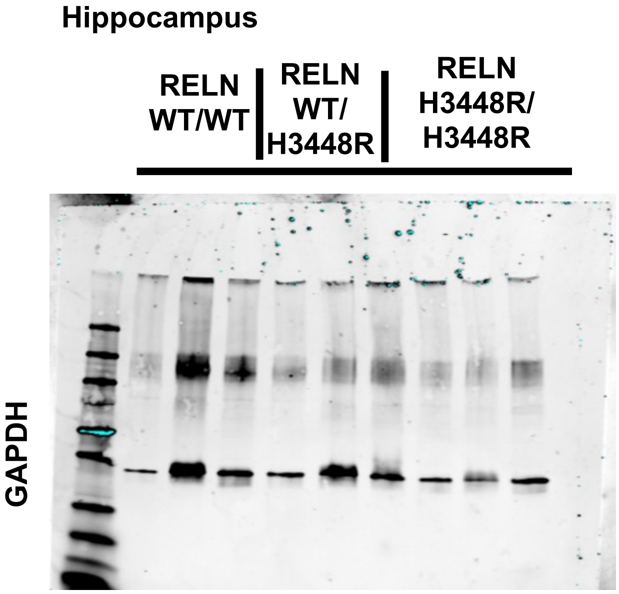

Full unprocessed scans for supplementary figure 11a.

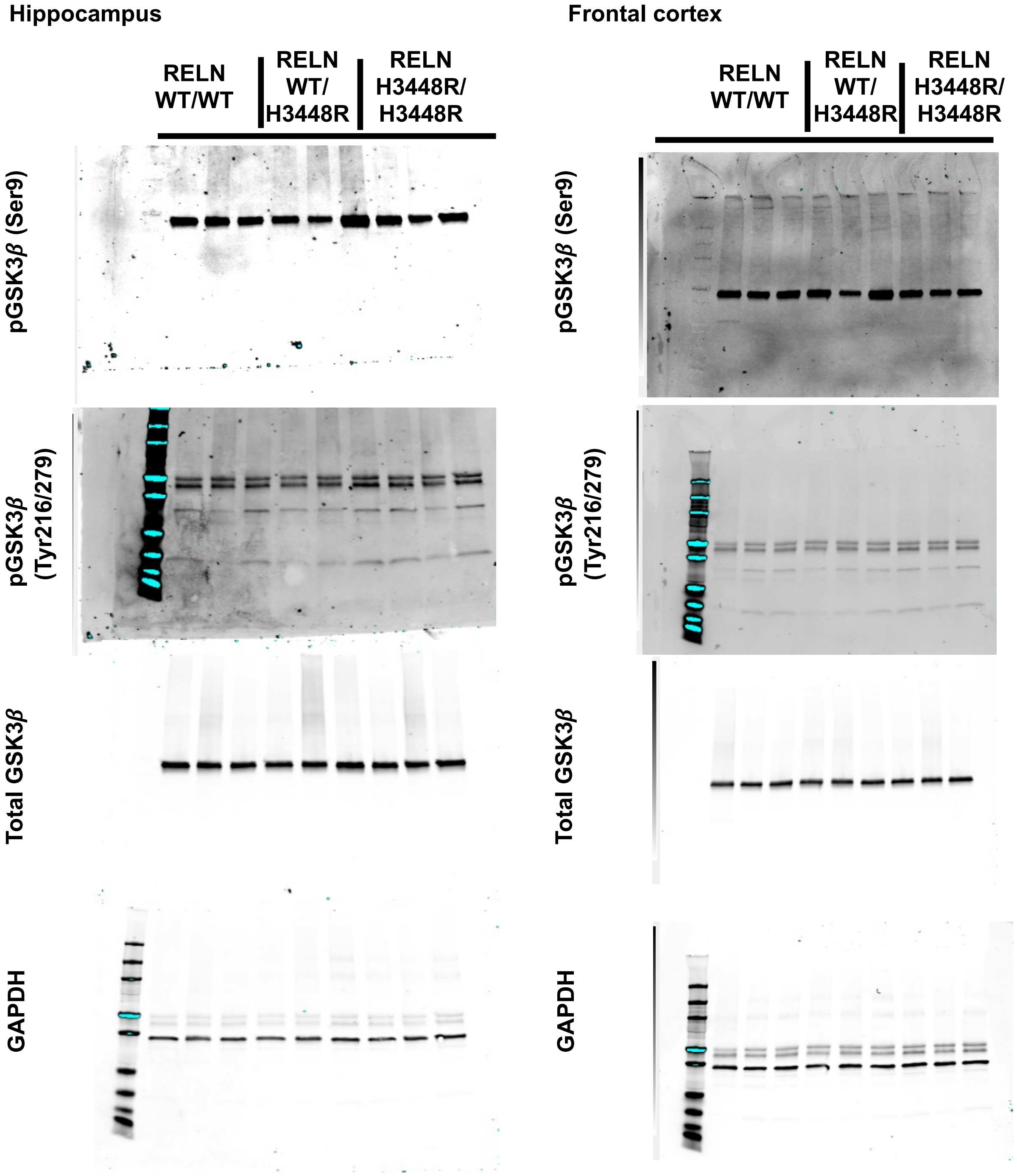

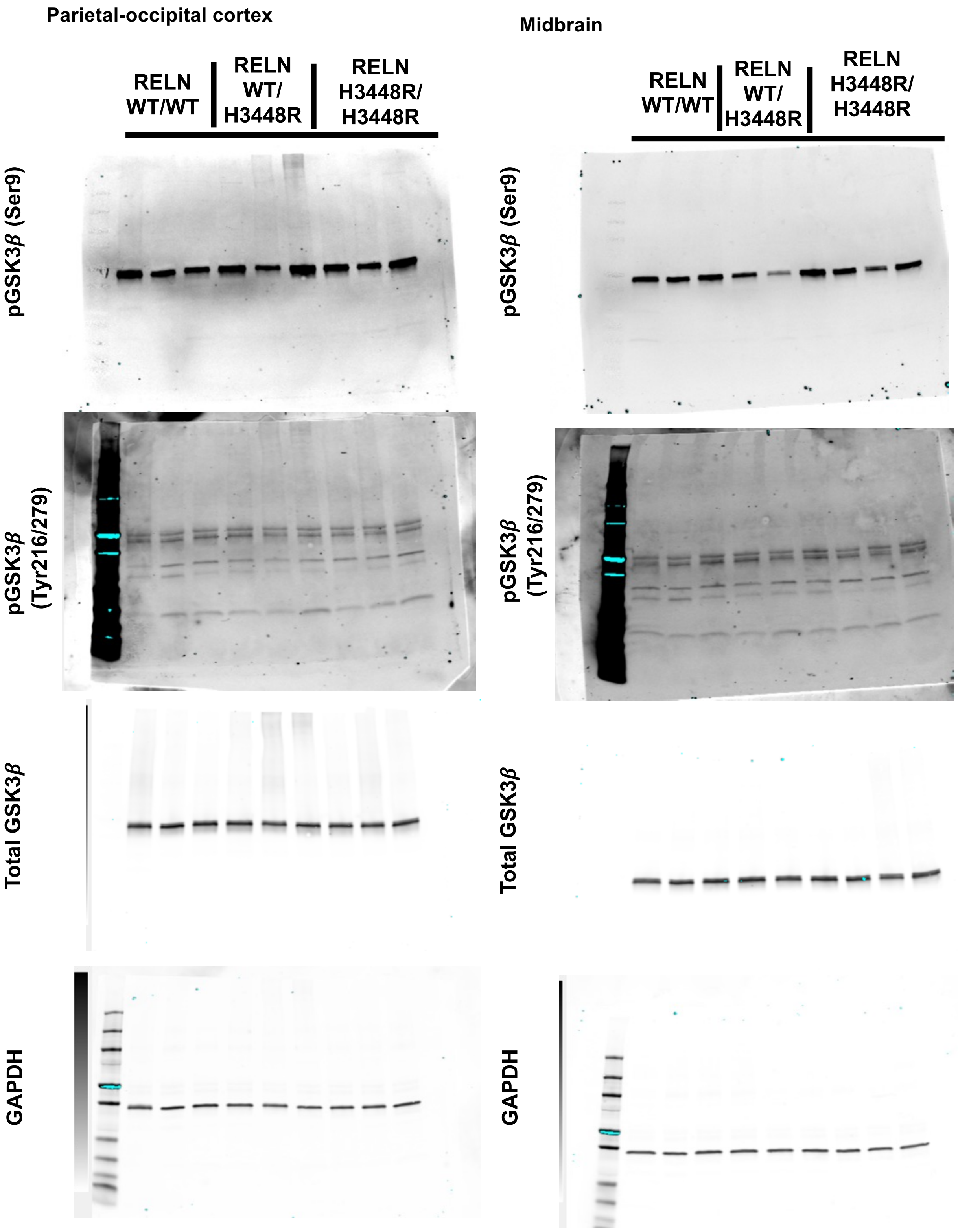
Parietal-occipital cortex
Midbrain
RELN WT/WT
RELN WT/ H3448R
RELN H3448R/ H3448R
pGSK3β (Ser9)
pGSK3β (Tyr216/279)
Total GSK3β
GAPDH

Cerebellum

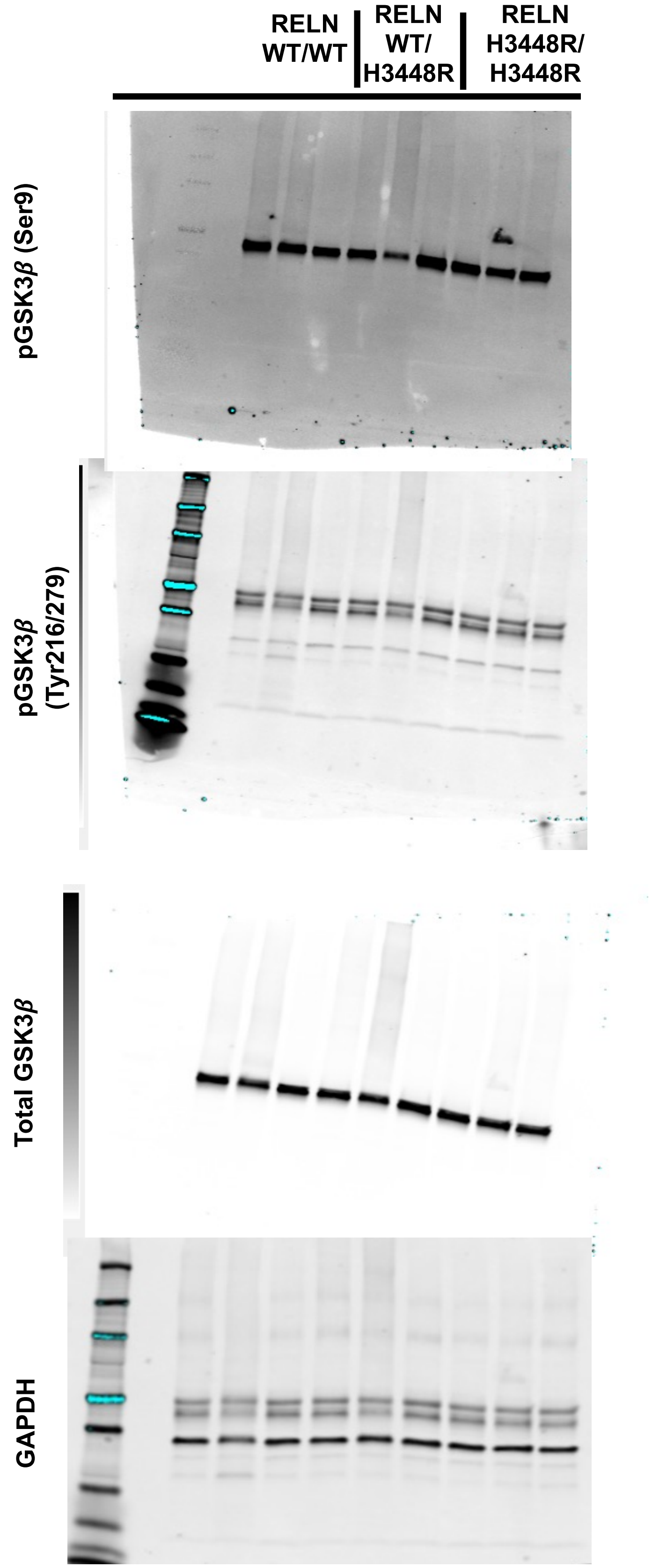
RELN WT/WT
RELN WT/ H3448R
RELN H3448R/ H3448R
pGSK3β (Ser9)
pGSK3β (Tyr216/279)
Total GSK3β
GAPDH

Full unprocessed scans for supplementary figures 12a.

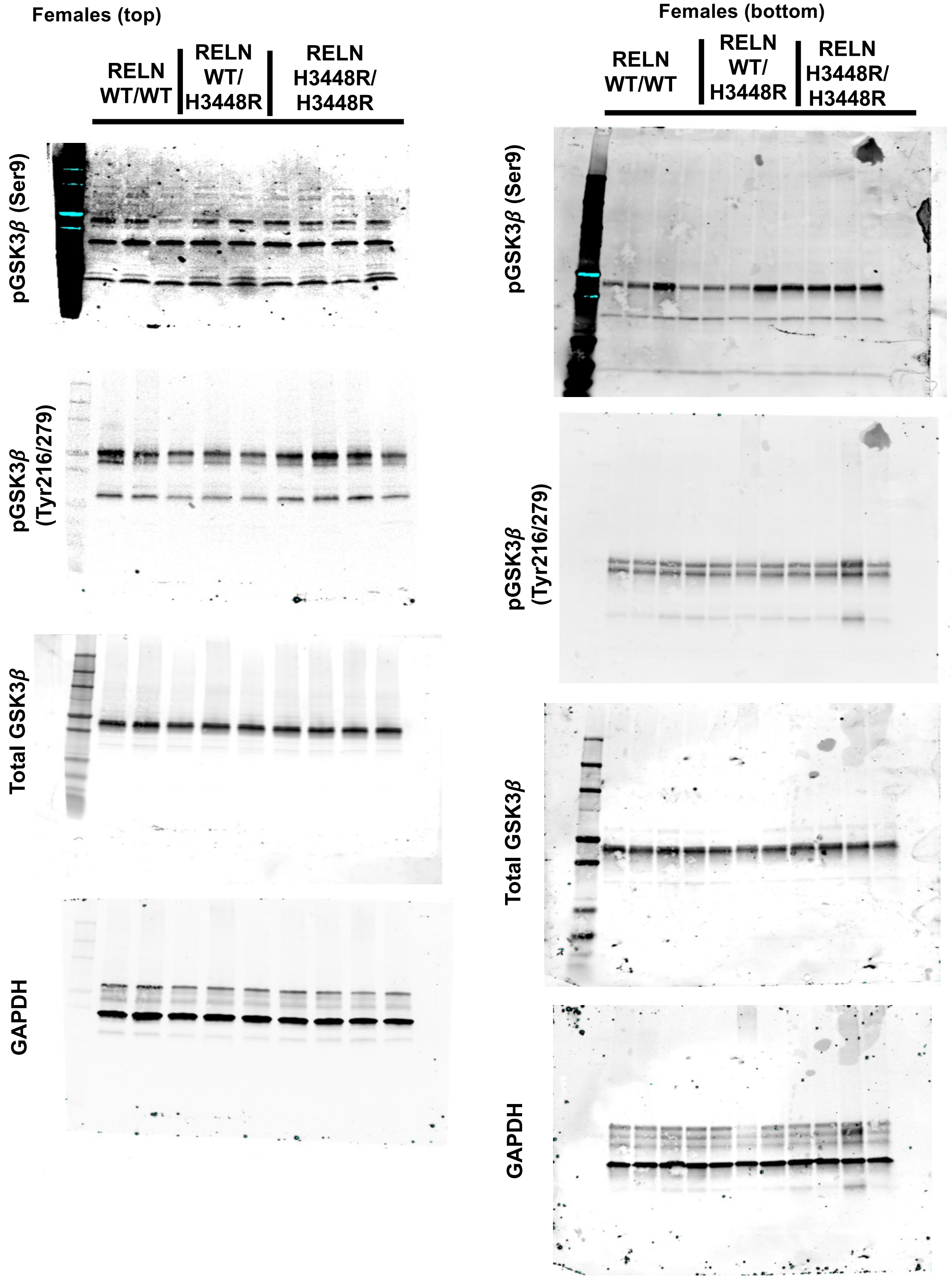

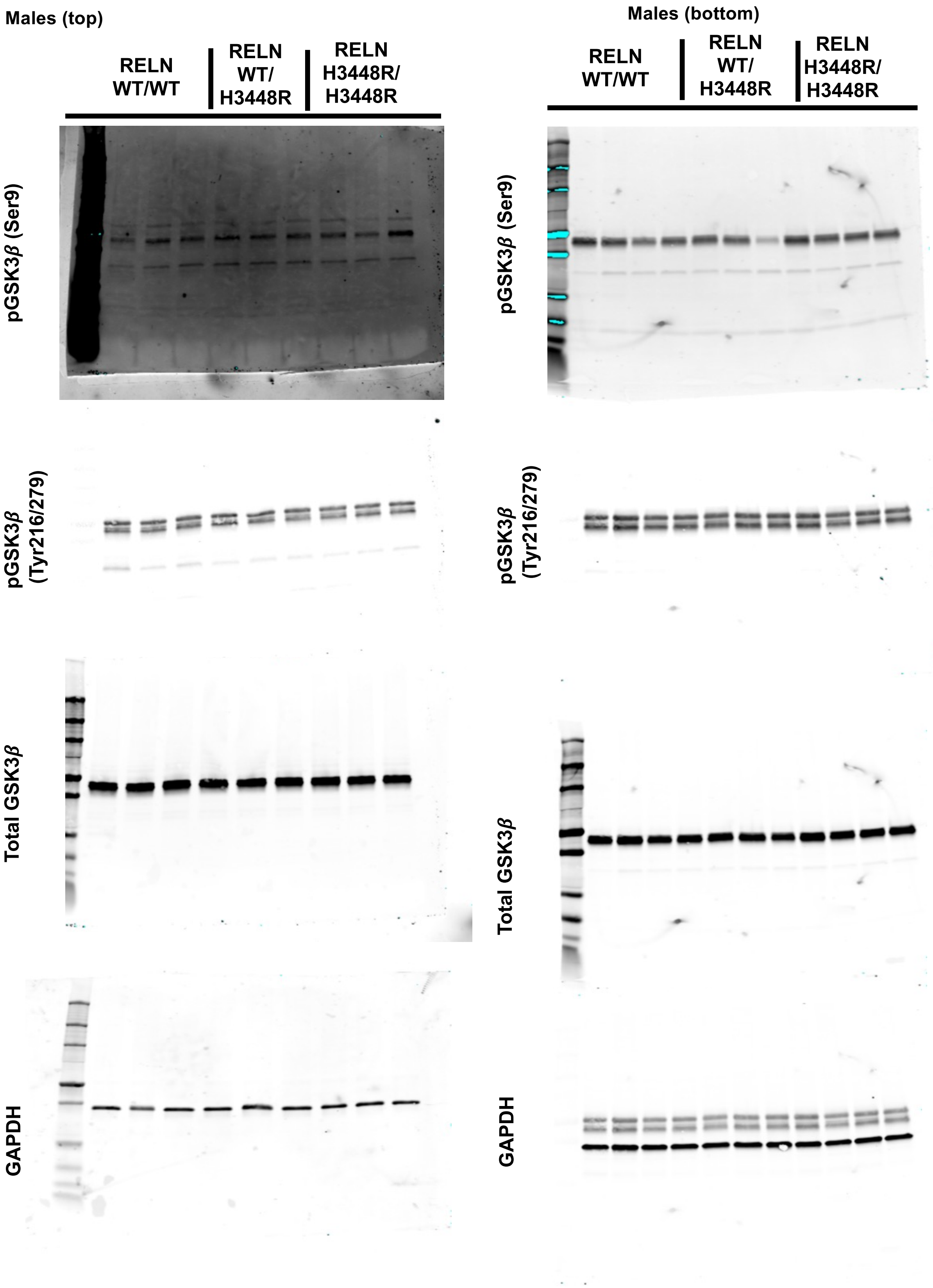
Males (top)
RELN WT/WT
RELN WT/H3448R
RELN H3448R/H3448R
pGSK3β (Ser9)
pGSK3β (Tyr216/279)
Total GSK3β
GAPDH
Males (bottom)
RELN WT/WT
RELN WT/H3448R
RELN H3448R/H3448R
pGSK3β (Ser9)
pGSK3β (Tyr216/279)
Total GSK3β
GAPDH
