# Supplementary material for: Resilience to autosomal dominant Alzheimer’s disease in a Reelin-COLBOS heterozygous man
Source: Nat Med. 2023 May 15;29(5):1243–52. doi: 10.1038/s41591-023-02318-3 (PMC10202812; doi:10.1038/s41591-023-02318-3)
Supplement: Supplementary file 2 — Reporting Summary [file 41591_2023_2318_MOESM2_ESM.pdf]

nature portfolio

Corresponding author(s): Joseph Arboleda-Velasquez
Yakeel T. Quiroz
Diego Sepulveda-Falla

Last updated by author(s): Apr 20, 2023

Reporting Summary

Nature Portfolio wishes to improve the reproducibility of the work that we publish. This form provides structure for consistency and transparency in reporting. For further information on Nature Portfolio policies, see our Editorial Policies and the Editorial Policy Checklist.

Statistics

For all statistical analyses, confirm that the following items are present in the figure legend, table legend, main text, or Methods section.

n/a	Confirmed	
☐	☒	The exact sample size (*n*) for each experimental group/condition, given as a discrete number and unit of measurement
☐	☒	A statement on whether measurements were taken from distinct samples or whether the same sample was measured repeatedly
☐	☒	The statistical test(s) used AND whether they are one- or two-sided<br>*Only common tests should be described solely by name; describe more complex techniques in the Methods section.*
☒	☐	A description of all covariates tested
☒	☐	A description of any assumptions or corrections, such as tests of normality and adjustment for multiple comparisons
☒	☐	A full description of the statistical parameters including central tendency (e.g. means) or other basic estimates (e.g. regression coefficient) AND variation (e.g. standard deviation) or associated estimates of uncertainty (e.g. confidence intervals)
☐	☒	For null hypothesis testing, the test statistic (e.g. *F*, *t*, *r*) with confidence intervals, effect sizes, degrees of freedom and *P* value noted<br>*Give P values as exact values whenever suitable.*
☒	☐	For Bayesian analysis, information on the choice of priors and Markov chain Monte Carlo settings
☒	☐	For hierarchical and complex designs, identification of the appropriate level for tests and full reporting of outcomes
☒	☐	Estimates of effect sizes (e.g. Cohen's *d*, Pearson's *r*), indicating how they were calculated

Our web collection on statistics for biologists contains articles on many of the points above.

Software and code

Policy information about availability of computer code

Data collection

High performance liquid chromatography (HPLC) data was collected on Shimadzu LabSolutions Version 5.101.; Whole exome and genome sequence data was collected at the Broad Institute, Cambridge, Massachusetts. Single cell RNA sequencing data was collected at Boston University Microarray Facility, Boston, Massachusetts. Western blotting data was collected the Odyssey Infrared imaging system connected to Image Studio Version 2.1 (LI-COR Biosciences),or GeneSys V1.5.3.0. softwares (blot probed with anti Dab1).
ELISA data acquisition and analysis: Gen 5 1.11 .Ink (Version 1.11.5).
Mass Spectrometry Data was collected as a fee-for-service at the Taplin Biological Mass Spectrometry Facility (Harvard Medical School, Boston, MA) using nano-scale reverse-phase HPLC capillary column upon gradient elution using acetonitrile and formic acid via an electrospray ionization-LTQ Orbitrap Velos Pro ion-trap mass spectrometer (Thermo Fisher Scientific, Waltham, MA).
Surface plasmon resonance data was collected using Biacore 3000 Control Software v4.1. Bio-layer interferometry data was collected using Octet Data Acquisition Software v9.0.

Data analysis

Whole exome and genome sequence data was analyzed using the following tools:
- Reference Genome – hs37d5 (ftp://ftp.1000genomes.ebi.ac.uk/vol1/ftp/technical/reference/phase2_reference_assembly_sequence/hs37d5.fa.gz)
- Edico Genome Dragen Pipeline Version - SW: 01.011.231.02.05.01.40152, HW: 01.011.231
- bcftools 1.9 (http://samtools.github.io/bcftools/)
- Ensembl VEP v94 (https://uswest.ensembl.org/info/docs/tools/vep/index.html)
- Gnomad r2.0.1 (http://gnomad.broadinstitute.org/downloads)
- bcbio nextgen tool suite v1.1.2 (https://github.com/bcbio/bcbio-nextgen)
- Exomiser v 10.1.0
- Cartagenia version 5.0 (https://www.genomeweb.com/resources/new-product/cartagenia-bench-lab-50)

- HGMD: Qiagen HGMD® Professional Database 2018.2
- OMIM: OMIM latest version at the time of analysis
- ExAC: ExAC release 0.3
- GnomAD: latest online version http://gnomad.broadinstitute.org/

The Genomizer, available as part of the Java application Exomiser (version 11.0.1), was used for the interpretation of non-coding variants including the splicing or regulatory variants, along with the Exomiser's variant prioritization on the coding variants. Inheritance mode: Genes filtered for compatibility with AUTOSOMAL_DOMINANT,AUTOSOMAL_RECESSIVE,X_RECESSIVE,X_DOMINANT,MITOCHONDRIAL inheritance. Explicitly removed variants with variant effects of type: [NON_CODING_TRANSCRIPT_INTRON_VARIANT, FIVE_PRIME_UTR_INTRON_VARIANT, THREE_PRIME_UTR_INTRON_VARIANT, INTERGENIC_VARIANT]

Single-cell RNA sequencing analysis:
- Raw sequencing data: Cell Ranger software suite v3.0.2 (demultiplexing, barcode processing, transcript counting and clustering analysis).
- Downstream analysis, feature-barcoding: Seurat R-package V3
- Clustering: Cell Ranger and viewed using Loupe Cell Browser.
- The gene-cell count: SCTransform algorithm V3
- Non-linear dimensional reduction: RunUMAP with the UMAP algorithm.

Precuneus to whole-brain cerebral metabolic rate for glucose (CMRgl) ratio analysis:
F-fludeoxyglucose PET image using an automated brain mapping algorithm (SPM8; http://www.fil.ion.ucl.ac.uk/spm/software/spm8).

Hippocampal to total intracranial volume ratio analysis:
FreeSurfer (http://surfer.nmr.mgh.harvard.edu)

Western Blotting data processing: Images were visualized using Image Studio Version 2.1 (LI-COR Biosciences), Microsoft PowerPoint for MacOS(Version 16.69.1), GraphPad Prism 9 (Version 9.4.1, La Jolla California), Image J 2.3.0/1.53q. (Blots presented in figure 2A, 3A Supplementary Figure 7, 8, 10, 11, and extended data Fig. 5)

ELISA data analysis: Gen 5 1.11 .Ink (Version 1.11.5); GraphPad Prism 8 (Version 8.1.1)

Circular dichroism (CD) data was deconvoluted using the online deconvolution software BeStSel (https://bestsel.elte.hu/).

Nuclear magnetic resonance (NMR) data Data was analyzed with NMRPipe and CCPNmr 2.4.2; structures were calculated with Cns 1.2.1 and aria 2.3.2.
NMR data were assessed in PyMol Version 2.3.3 (https://pymol.org/).

Bio-layer interferometry (BLI) experimental data were fit with the 1:1 binding model and analyzed with global fitting using Octet Data Analysis software to calculate KD.

Isothermal titration calorimetry (ITC) data was collected and evaluated using the MicroCal iTC200 Evaluation software (GE).

Surface plasmon resonance data was collected using BIAevaluation software v4.2.

Bio-layer interferometry data was collected using Octet Data Analysis Software v9.0

Raw data was collected in GraphPad Prism 8 or 9 (8.1.1, or Version 9.4.1, La Jolla California). The same software was also used to generate figures.

Mass Spectrometry data was analyzed using Sequest (Thermo Fisher Scientific, Waltham, MA).
Histopathology data analysis was performed using GraphPad 8 (8.1.1) and R Software (ver. 3.6.3).

For manuscripts utilizing custom algorithms or software that are central to the research but not yet described in published literature, software must be made available to editors and reviewers. We strongly encourage code deposition in a community repository (e.g. GitHub). See the Nature Portfolio guidelines for submitting code & software for further information.

Data

Policy information about availability of data

All manuscripts must include a data availability statement. This statement should provide the following information, where applicable:

- Accession codes, unique identifiers, or web links for publicly available datasets
- A description of any restrictions on data availability
- For clinical datasets or third party data, please ensure that the statement adheres to our policy

Anonymized clinical, genetic, and imaging data are available upon request during working hours, subject to an internal review by F.L., J.F.A.-V., and Y.T.Q. to ensure that the participants’ confidentiality, and PSEN1 E280A carrier or non-carrier status are protected, completion of a data sharing agreement, and in accordance with University of Antioquia’s and Massachusetts General Hospital’s IRB and institutional guidelines. Experimental data is available upon request, subject to Massachusetts General Hospital and Schepens Eye Research Institute of Mass Eye and Ear institutional guidelines. Material requests and data requests will be considered based on a proposal review, completion of a material transfer agreement and/or a data use agreement, and in accordance with the Massachusetts General Hospital and Schepens Eye Research Institute of Mass Eye and Ear institutional guidelines. Please submit requests for participant-related clinical and imaging data and samples to Y.T.Q. (yquiroz@mgh.harvard.edu); and, requests for experimental data and materials, genetic and single- cell RNA sequencing data to J.F.A.-V. (joseph_arboleda@meei.harvard.edu); and, requests for neuropathology specimens to F.L. (francisco.lopera@gna.org.co). The RELN COLBOS mouse model will be made freely available to the community via the Mutant Mouse Resource and Research Centers repository (MMRRC). NMR structure is available via PDB.org platform (DOI). Whole exome and genome sequence data was analyzed using the following tools and databases: Genome – hs37d5 (ftp://ftp.1000genomes.ebi.ac.uk/vol1/ftp/technical/reference/phase2_reference_assembly_sequence/hs37d5.fa.gz); Edico Genome Dragen Pipeline Version - SW: 01.011.231.02.05.01.40152, HW: 01.011.231; bcftools 1.9 (http://samtools.github.io/bcftools/); Ensembl VEP v94 (https://uswest.ensembl.org/info/docs/tools/vep/

index.html); Gnomad r2.0.1 (http://gnomad.broadinstitute.org/downloads); bcbio nextgen tool suite v1.1.2 (https://github.com/bcbio/bcbio-nextgen); Exomiser v 10.1.0; Cartagenia version 5.0: https://chla.ngs.cartagenia.com/; HGMD: Qiagen HGMD® Professional Database 2018.2; OMIM: OMIM latest version at the time of analysis; ExAC: ExAC release 0.3 http://exac.broadinstitute.org/; GnomAD: latest online version http://gnomad.broadinstitute.org/

Human research participants

Policy information about studies involving human research participants and Sex and Gender in Research.

Reporting on sex and gender	We reported data for men and women according to their reported sex. We reported potential sexual dimorphism for the protective phenotype.
Population characteristics	All research participants belonged to an extended family with autosomal dominant Alzheimer's disease from Colombia. Only individuals older than 28 years old were invited to participate, given previous findings of evidence of amyloid pathology in their brains starting at that age. Both women and men were invited to participate.
Recruitment	All participants were recruited from the Colombia Alzheimer's Prevention Registry, which currently has more than 6,000 members. This registry is maintained by the Group of Neurosciences of the University of Antioquia (PI: Francisco Lopera). Recruited participants were compensated.<br><br>Informed consent from subjects include their agreement to not know their genetic status while asymptomatic. Local researchers minimize the possibility of self-selection bias by facilitating access to participation by traveling to the locations where the subjects reside.
Ethics oversight	Inclusion & ethics in global research<br>The study has IRB approval from Massachusetts general hospital, the Mass Eye and Ear of Boston, MA, and the local Ethics Committee at the Universidad the Antioquia.<br>This work involves a collaboration between scientist in multiple countries including Colombia, USA, and Germany. Contributors from all sites are included as co-authors or in acknowledgments according to their contributions. Researchers residing in Colombia have been involved in study design, study implementation, data ownership and intellectual property as appropriate. The research is locally relevant due to high prevalence of ADAD. Roles and responsibilities were agreed amongst collaborators ahead of the research. Local ethics committees approved all research involving human subjects. To prevent any stigmatization any and all identifying information has been removed to preserve subjects privacy. The Colombian team has retained ownership of any and all human biological materials shared for research purposes.

Note that full information on the approval of the study protocol must also be provided in the manuscript.

Field-specific reporting

Please select the one below that is the best fit for your research. If you are not sure, read the appropriate sections before making your selection.

☒ Life sciences ☐ Behavioural & social sciences ☐ Ecological, evolutionary & environmental sciences

For a reference copy of the document with all sections, see nature.com/documents/nr-reporting-summary-flat.pdf

Life sciences study design

All studies must disclose on these points even when the disclosure is negative.

Sample size	For clinical data: Sample size of subjects for comparison studies was defined solely by the availability of subjects with relevant information studied under similar conditions. For data presented in Figure 1: According to IRB regulation, PET imaging could not involve repeated measurements.<br>For all other data: sample size was stated in both methods and figure legends for each biological experiments. Sample size calculations are not applicable for the functional assays and structural determination we used. For western blotting analyses we conducted a power analysis to determine the minimum statistically significant sample size. For cell-free experiments we arbitrarily determine sample size.
Data exclusions	No data was excluded.
Replication	For Clinical data, 11 impaired and 18 unimpaired carriers were analyzed as reported in Supplementary Table 2.<br>Brain imaging for the male subject was only conducted once because of feasibility and availability of the patient. Data on amyloid and tau pathology was validated postmortem via immunohistology. Accordingly, all attempts for replication were confirmed.<br>We thoroughly explored independent experimental designs to determine RELN biology and functional interactions with heparin.<br>Data presented in Fig. 2A is expressed as mean ± s. e. m of n = 4 independent biological experiments tested on individual blots.<br>For SPR and BLI data, a range of untagged (SPR) or Fc-fusion peptides (BLI) were used to determine the KD. The determined rate constants are independent of sample concentration.<br>Data presented in Fig. 4A, Supplementary Fig. 8, 9, 11, 12, and extended data Fig. 5 is expressed as the average of brain homogenates obtained from different mice as showed in the blots. N = 3 mice were use for 3 m.o., 6 m.o., 12 m.o. (H3448R/H3448R only) mice. n = 4 mice were used for 12 m.o. WT or WT/H3448R as indicated in the figure legend. When available both male and females were used.<br><br>Gravity heparin- affinity chromatography data (Fig. 2 B) was expressed as average -/+ SEM of n = 3 independent sample runs from different

sample preparation, HPLC data (Fig 3) was replicated with an n = 2 independent sample runs from the same sample preparation.

CD data (Extended Data Fig. 3) was not replicated, but the data was confirmed with NMR (Fig 2F, 3A, Extended Data Fig. 3). For NMR, in total 200 structures were calculated, and the 20 lowest energy structures were selected.

Calorimetry data (ITC, Fig. 2) data was plotted as the average of four spectra.

ELISA data presented in Supplementary Figure 5 is replicated by n = 2 independent experiments of n = 3 technical replicates.

Data presented in Extended Data Fig. 4 was replicated by analyzing at least n = 5 image field from n = 4 (WT) of n = 3 (WT/H3448R, H3448R/H3448R) specimens obtained from different mice.

Data presented in Figure 4 D, Supplementary Figures 10 and 13 were replicated by analyzing images from n = 3 specimens per genotype. Data was replicated twice.

Data presented in Figure 4G was replicated by scoring behavior on n = 13 RELNWT/Tau-P301L mice and n = 11 RLN-H3448R H3448R/Tau-P301L crossed male mice.

Randomization

Subjects are randomized to ensure inclusion of mutation carriers. Mice were also randomized by giving a masked numerical ID to perform unbiased behavioral paradigms and postmortem analyses. Biophysical and in vitro analyses were randomized using alphanumerical coding that was decoded post analysis. Cell culture experiments were randomized by masking treatments using alphanumerical coding.

Blinding

Clinicians were blinded regarding the mutation status of the carriers. Investigators were blinded for data collection and analysis of circular dichroism, NMR structural determination, isothermal titration calorimetry, bio-layer interfermetry, histological analyses, western blotting and behavioral studies.

Reporting for specific materials, systems and methods

We require information from authors about some types of materials, experimental systems and methods used in many studies. Here, indicate whether each material, system or method listed is relevant to your study. If you are not sure if a list item applies to your research, read the appropriate section before selecting a response.

Materials & experimental systems

n/a	Involved in the study	
☐	☒	Antibodies
☐	☒	Eukaryotic cell lines
☒	☐	Palaeontology and archaeology
☐	☒	Animals and other organisms
☒	☐	Clinical data
☒	☐	Dual use research of concern

Methods

n/a	Involved in the study	
☒	☐	ChIP-seq
☒	☐	Flow cytometry
☐	☒	MRI-based neuroimaging

Antibodies

Antibodies used

Primary antibodies for western blotting: β-Tubulin (ms; 1:2,000; 86298S, Cell Signaling, lot. 3), anti-phospho-Dab1 (Rb; 1:7,500; MBS8511213, MyBiorsorce, lot. B14D06), anti-phospho-GSK3β-Ser9 (1:1,000, D85E12, Cell Signaling, lot. 55585), anti-phospho-GSK3β-Tyr216/279 (1:1,000, 05-413, Millipore, lot. 8337766), anti-GSK3β (1,1000, 5558, Cell Signaling, lot.8), anti-GAPDH (1:5,000, ab8245, Abcam, lot GR3428701-1), anti RELN antibody (ms, 1:1,000, clone CR-50, D223-3, MBL, lot. 017). Secondary antibodies for WB: IRDye 800CW donkey anti-mouse IgG secondary antibody (1:10,000, cat. 925-32212, Licor); IRDye 680CW donkey anti-rabbit IgG secondary antibody(1:10,000, cat. 925-68073, Li-COR). Rabbit IgG-HRP conjugated antibody (1:2000, HAF008, R&D Systems) and Super Signal™ West Pico PLUS Chemiluminescent Substrate (undiluted, cat.34580, Thermo Fisher).
Antibodies used for staining of fixed tissue: for amyloid beta (BAM-10, 1:100; Mob410, lot. Q319; DBS Emergo Europe, The Hague, The Netherlands), hyperphosphorylated tau (AT8, 1:100; MN1020, lot. PA198065; Thermo Fisher, Dreieich, DE), phosphorylated tau (T205, 1:10000; EPR23505-13, lot. GR3355978-1; Abcam, Hilversum, Netherlands), ionized calcium-binding adaptor molecule 1 (Iba1, 1:500; 019-19741, lot. WTF4691; Wako, Neuss, Germany), glial fibrillary acidic protein (GFAP, 1:200; M0761, lot. 41327882; DAKO GmbH, Jena, DE), anti-neuronal nuclei antibody (NeuN Clone A60, 1:100; MAB377, lot. 3604693, Merck/Millipore, Darmstadt, Germany), and specific secondary antibodies anti-mouse and anti- rabbit (P0260, lot. H2211 and P0447, lot. H221U respectively, DAKO GmbH, Jena, DE), Histofine Simple Stain MAX PO ® Anti-Goat, Immuno-peroxidase Polymer (414161F, lot. H2204A Nichirei Bioscience Inc., Tokyo, Japan,). Visualization was achieved with 3,3’-Diaminobenzidine (DAB, Ventana, 92 760-500, lot. 10 J19566 Roche AG, Basel, Switzerland) and the Ultraview Universal Detection Kit (92 760-500, lot. 10 J19566, Roche AG, Basel, Switzerland) according to manufacturer instruction.

Validation

We only used commercially available antibodies that were previously validated by the relative companies. For validation, on supplier websites are shown either ELISA or WB data using positive and or negative controls to validate each target.

Eukaryotic cell lines

Policy information about cell lines and Sex and Gender in Research

Cell line source(s)	The Fc-fusion peptides were produced in HEK293 cells as a fee-for-service by Innovagen AB (Lund, Sweden). HEK293 cells were purchased by Innovagen from Invitrogen (cat. 51-0029). Primary CD1 brain cortex mouse neurons obtained from day 14/15 embryos of mixed sex (M-CX-400, Lonza) and Flp-In T Rex 293 mammalian cells (R78007, Thermo Fisher Scientific) cell line.
Authentication	All cell lines were authenticated by resistance/susceptibility to the antibodies listed by the providers Innovagen AB (HEK293) and Lonza (M-CX-400, cell type further confirmed via IHC and ELISA using GFAP, MAP2, and Tuj, IHC only) and Thermo Fischer (R78007)
Mycoplasma contamination	Reported by the providers to be negative via PCR
Commonly misidentified lines (See ICLAC register)	The cell line we used is not listed as misidentified lines according to the "ICLAC Register of misidentified cell lines V11".

Animals and other research organisms

Policy information about studies involving animals; ARRIVE guidelines recommended for reporting animal research, and Sex and Gender in Research

Laboratory animals	RELNH3448R-Tg knock in (KI) mouse generated at Taconic, Tg(Prnp-MAPT*P301L)JNPL3Hlmc mouse model from Taconic. Both stains were crossed and different offspring genotypes analyzed as indicated. For all experiments, animals were housed at the Animal Care Facility of the Schepens Eye Research Institute, Boston, MA according to IACUC approved protocols. Animals were housed with regular light/dark cycle, controlled air flow and regular diet with ad libitum access. When specified, both males and females were tested. For post mortem analyses 3 m.o., 6 m.o. 12 m.o. and 18 m.o. mice were tested.
Wild animals	No wild animals were used in the study.
Reporting on sex	When possible we reported data for both, male and female sex. Findings were statistically significant in males when indicated by the statistical analysis.
Field-collected samples	No field collected samples were used in the study.
Ethics oversight	Animal studies were approved by The Schepens Eye Research Institute and the Institutional Animal Care and Use Committee (IACUC).

Note that full information on the approval of the study protocol must also be provided in the manuscript.

Magnetic resonance imaging

Experimental design

Design type	Structural MRI.
Design specifications	none
Behavioral performance measures	none

Acquisition

Imaging type(s)	Structural MRI, T1 images.
Field strength	3 Tesla MRI scanner.
Sequence & imaging parameters	T1 3-D images.
Area of acquisition	Whole brain was acquired. Volumetric analysis focused on the hippocampi.

Diffusion MRI ☐ Used ☒ Not used

Preprocessing

Preprocessing software	Freesurfer (http://surfer.nmr.mgh.harvard.edu).
Normalization	n/a
Normalization template	n/a

Noise and artifact removal | n/a

Volume censoring | n/a

Statistical modeling & inference

Model type and settings | Volumetric analyses.

Effect(s) tested | n/a

Specify type of analysis: ☐ Whole brain ☒ ROI-based ☐ Both

Anatomical location(s) | Hippocampal volume

Statistic type for inference (See Eklund et al. 2016) | n/a

Correction | n/a

Models & analysis

n/a	Involved in the study
☒	☐ Functional and/or effective connectivity
☒	☐ Graph analysis
☒	☐ Multivariate modeling or predictive analysis
