# Supplementary figures and images for: Resilience to autosomal dominant Alzheimer’s disease in a Reelin-COLBOS heterozygous man
Source: Nat Med. 2023 May 15;29(5):1243–52. doi: 10.1038/s41591-023-02318-3 (PMC10202812; doi:10.1038/s41591-023-02318-3)

Source data for Figure 2: Uncropped western blotting

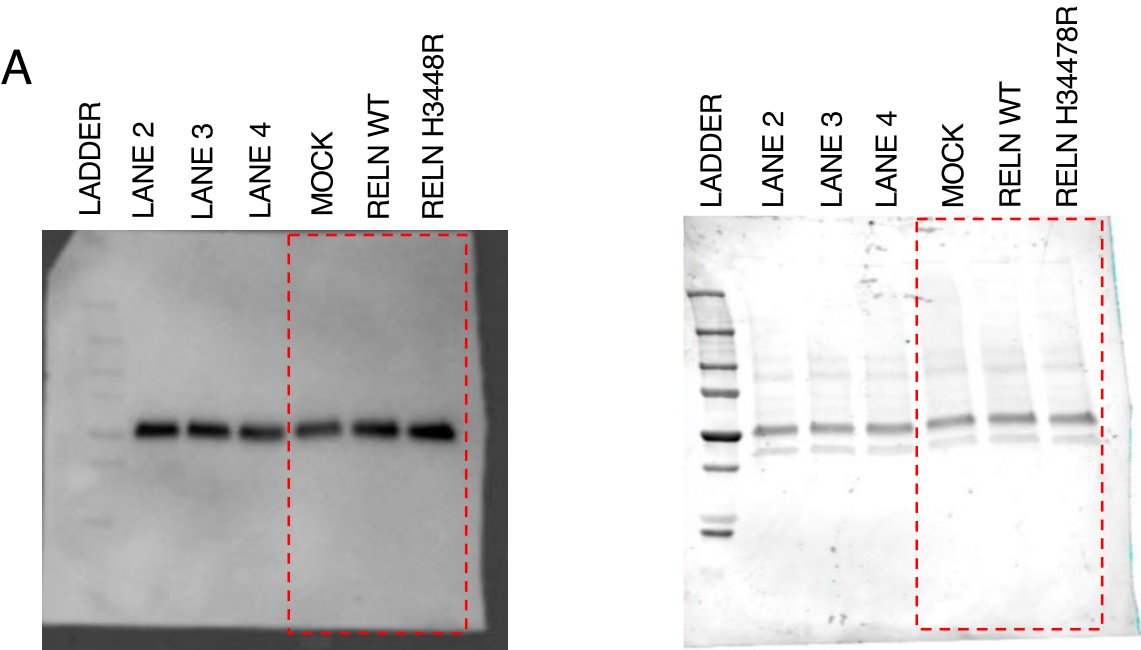

Supplement: Source Data Fig. 2 — Unprocessed western blots. [file 41591_2023_2318_MOESM6_ESM.pdf]

Source data for Figure 4a: Uncropped western blotting

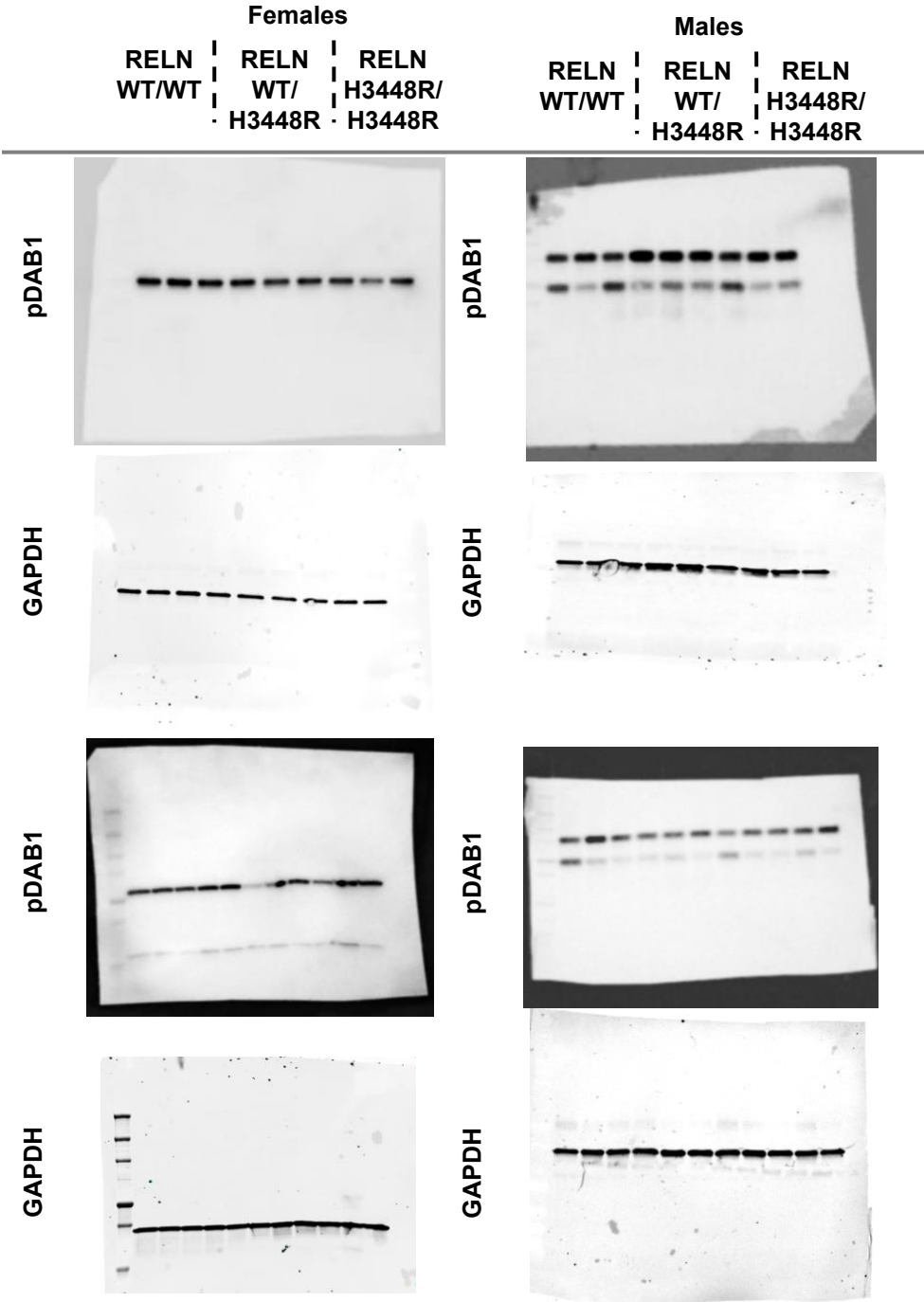

Supplement: Source Data Fig. 4 — Unprocessed western blots. [file 41591_2023_2318_MOESM7_ESM.pdf]
