# Supplementary material for: Resilience to autosomal dominant Alzheimer’s disease in a Reelin-COLBOS heterozygous man
Source: Nat Med. 2023 May 15;29(5):1243–52. doi: 10.1038/s41591-023-02318-3 (PMC10202812; doi:10.1038/s41591-023-02318-3)

Full unprocessed scans for Extended Data Figure 5a.

Hippocampus

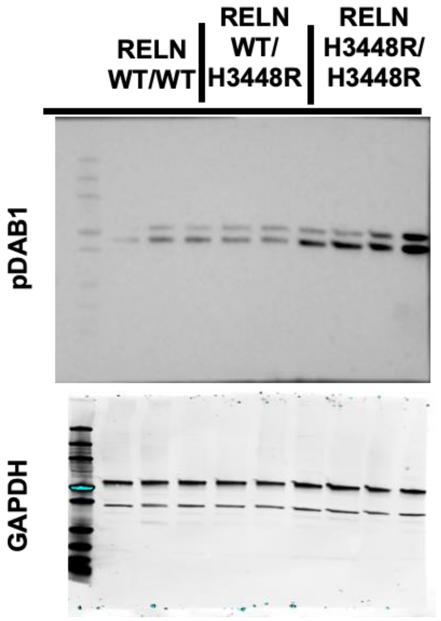

Frontal cortex

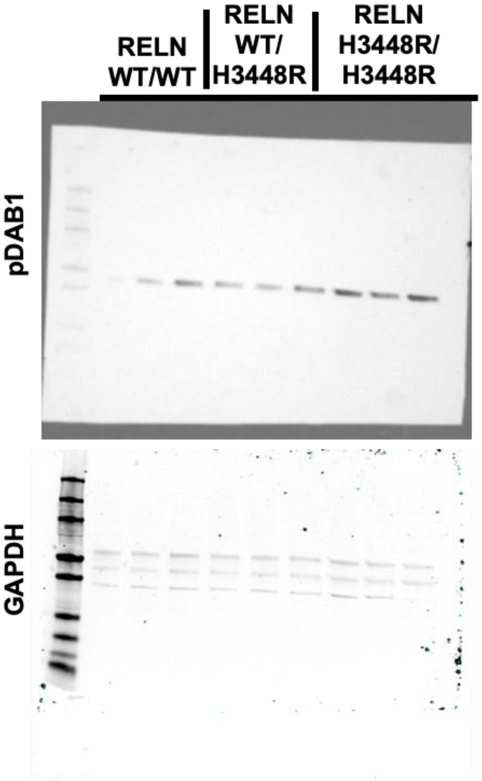

Parietal-occipital cortex

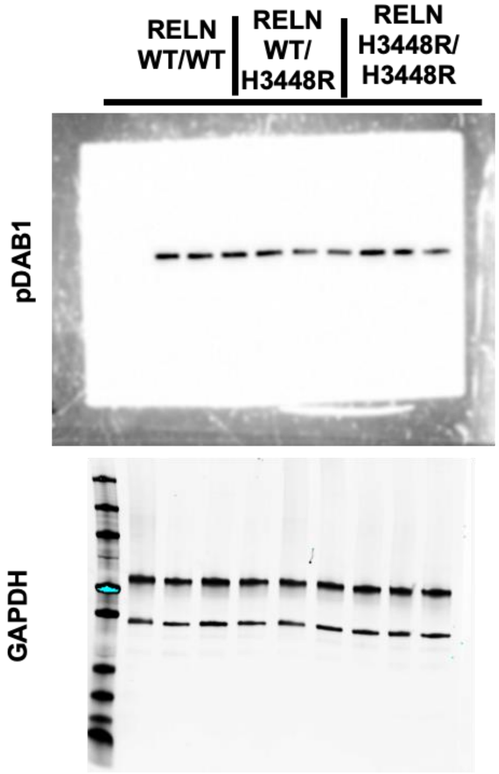

Supplement: Source Data Extended Data Fig. 5 — Unprocessed western blots. [file 41591_2023_2318_MOESM8_ESM.pdf]
